# Phase stability of the nanolaminates V_2_Ga_2_C and (Mo_1–*x*_V_*x*_)_2_Ga_2_C from first-principles calculations†
†Electronic supplementary information (ESI) available. See DOI: 10.1039/c6cp00802j



**DOI:** 10.1039/c6cp00802j

**Published:** 2016-04-20

**Authors:** A. Thore, M. Dahlqvist, B. Alling, J. Rosen

**Affiliations:** a Department of Physics, Chemistry, and Biology , Thin Film Physics Division , Linköping University , SE-581 83 Linköping , Sweden . Email: andth@ifm.liu.se ; Email: madah@ifm.liu.se ; Email: bjoal@ifm.liu.se ; Email: johro@ifm.liu.se ; Tel: +46 70 3212109; b Max-Planck-Institut für Eisenforschung GmbH , D-402 37 Düsseldorf , Germany

## Abstract

We here use first-principles calculations to investigate the phase stability of the hypothetical laminated material V_2_Ga_2_C and the related alloy (Mo_1–*x*_V_*x*_)_2_Ga_2_C, the latter for a potential parent material for synthesis of (Mo_1–*x*_V_*x*_)_2_C, a new two-dimensional material in the family of so called MXenes. We predict that V_2_Ga_2_C is thermodynamically stable with respect to all identified competing phases in the ternary V–Ga–C phase diagram. We further calculate the stability of ordered and disordered configurations of Mo and V in (Mo_1–*x*_V_*x*_)_2_Ga_2_C and predict that ordered (Mo_1–*x*_V_*x*_)_2_Ga_2_C for *x* ≤ 0.25 is stable, with an order–disorder transition temperature of ∼1000 K. Furthermore, (Mo_1–*x*_V_*x*_)_2_Ga_2_C for *x* = 0.5 and *x* ≥ 0.75 is suggested to be stable, but only for disordered Mo–V configurations, and only at elevated temperatures. We have also investigated the electronic and elastic properties of V_2_Ga_2_C; the calculated bulk, shear, and Young's modulus are 141, 94, and 230 GPa, respectively.

## Abstract 2

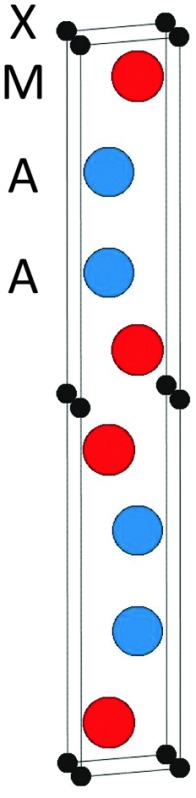
We here use first-principles calculations to investigate the phase stability of the hypothetical laminated material V_2_Ga_2_C and the related alloy (Mo_1–*x*_V_*x*_)_2_Ga_2_C, the latter for a potential parent material for synthesis of (Mo_1–*x*_V_*x*_)_2_C, a new two-dimensional material in the family of so called MXenes.

## I. Introduction

In a recent paper by Hu *et al.*,^1^ the discovery of a new crystalline phase, Mo_2_Ga_2_C, was reported, suggested to be the first member of a family of hitherto unknown materials closely related to the M_*n*+1_AX_*n*_ (MAX) phase family. The latter consists of a large number of hexagonal, layered materials based on a transition metal (M), an A-group element (A) and C or N (X), which display a combination of metallic and ceramic properties.^2^ The structure of Mo_2_Ga_2_C has been further investigated by Lai *et al.*, who used a combination of theoretical calculations and experimental measurements to determine the structure, showing that the phase is indeed very similar to the MAX phase Mo_2_GaC, with C atoms residing between the Mo layers in an octahedral position and the Ga atoms being stacked in a simple hexagonal arrangement.^3^

Mo_2_GaC and Mo_2_Ga_2_C have been synthesized in bulk as well as thin film form;^1,3–5^ however, only the latter material has been converted to Mo_2_C, a so called MXene.^6^ Experimental results strongly suggest that Mo_2_GaC, to date the only known Mo-containing ternary MAX phase, cannot be used for this purpose,^6,7^ even though MXenes, which comprise a class of two-dimensional materials, are typically obtained from MAX phases through selective etching of the A layer. The properties of Mo_2_C remains to be explored, although the first report on the topic suggests that it exhibits 2D superconductivity, based on the observation that the critical magnetic field is significantly increased when the field direction with respect to the plane of the film is changed from perpendicular to parallel.^8^

To form a solid solution of two elements on a sublattice – in particular elements with different numbers of valence electrons – opens up for the possibility of tailoring the physical properties, something which has previously been demonstrated for quaternary MAX phases. For example, incorporation of 20 at% of V on the M sublattice of Ti_2_AlC leads to improvements in Vicker's hardness, flexural strength, and shear strength.^9^ Motivated by this we here investigate and predict the phase stability as well as the electronic and elastic properties of a new M_2_A_2_X phase (or 221 phase) with a single element on the M sublattice, V_2_Ga_2_C. We further investigate the stability of a 221 alloy (Mo_1–*x*_V_*x*_)_2_Ga_2_C for different Mo_1–*x*_V_*x*_ concentrations.

Apart from the possibility of tuning of properties in (Mo_1–*x*_V_*x*_)_2_Ga_2_C for different stoichiometries, synthesis of this phase would provide a promising experimental pathway for realizing 2D (Mo_1–*x*_V_*x*_)_2_C, a hypothetical new MXene alloy. While it is possible to obtain V_2_C by etching away the Al layer in the MAX phase V_2_AlC, the requirement of Mo_2_Ga_2_C for synthesis of Mo_2_C suggests that the parent phase for (Mo_1–*x*_V_*x*_)_2_C is (Mo_1–*x*_V_*x*_)_2_Ga_2_C, and not the corresponding MAX phase alloy. The here presented results predict that the new phases V_2_Ga_2_C as well as (Mo_1–*x*_V_*x*_)_2_Ga_2_C are stable.

## II. Calculation details

All calculations were carried out within the framework of density functional theory (DFT),^10^ as implemented in the Vienna *ab initio* simulation package (VASP),^11–14^ using the Perdew–Burke–Ernzerhof generalized gradient approximation (PBE-GGA) as the exchange–correlation energy functional.^15^ The plane wave cutoff energy for the structural relaxations was set to 400 eV, and Monkhorst–Pack grids were used for the Brillouin zone samplings. The free energy convergence criterion for all pure phases was 0.1 meV per atom, whereas for the alloy it was chosen to be 0.5 meV per atom due to the requirement of large supercells (4 × 4 × 1, containing 160 atoms). The dynamical matrix of V_2_Ga_2_C was calculated using *Γ*-centered density functional perturbation theory (DFPT), and the phonon dispersion was extracted with the help of the PHONOPY software package.^16,17^

All phase stability predictions in this work are based on a method for convex hull construction developed by Dahlqvist *et al.* which combines first-principles calculations with a linear optimization procedure (the simplex algorithm).^18^ This method has previously been successfully used to predict the existence of several MAX phases,^19–21^ and it also reproduces the stability of several already synthesized MAX phases, as well as the stability of Mo_2_Ga_2_C.^3,22^

For disordered (Mo_1–*x*_V_*x*_)_2_Ga_2_C, the supercells were created using the special quasirandom structure (SQS) methodology developed by Zunger *et al.* ^23^ The same approach was used for competing phases that were alloyed on one or more sublattices, and/or contained vacancies. For ordered (Mo_1–*x*_V_*x*_)_2_Ga_2_C, the enthalpies of a number of different, manually constructed M sublattice configurations were calculated and compared, all of which are listed in Table S2 in ESI.†

The enthalpy of formation of V_2_Ga_2_C and ordered (Mo_1–*x*_V_*x*_)_2_Ga_2_C, which was calculated through the linear optimization procedure described in ref. 22, can be expressed as

1 Δ*H*_cp_ = *H*_221_ – *H*_cp_,

where *H*_221_ is the enthalpy of the 221 phase and *H*_cp_ is the total enthalpy of the set of most competing phases. Formally, *H*_cp_ is defined as2
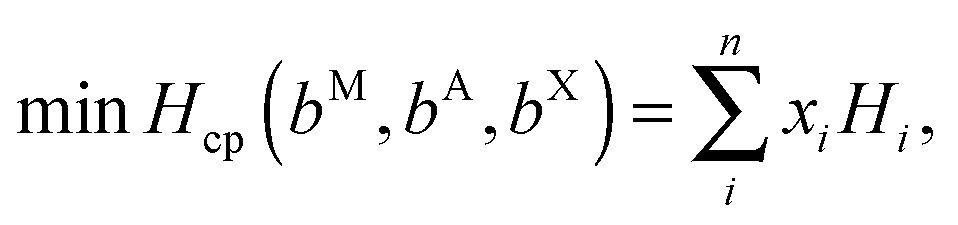
where *b* ^M,A,X^ is the amount of M, A, and X atoms in the 221 phase, *x*_*i*_ is the amount of the competing phase *i*, and *H*_*i*_ its enthalpy.^22^ The weighting factors *x*_*i*_ must be chosen so that the total amount of each atomic species in the set of competing phases is the same as in the 221 phase, which means that the *x*_*i*_'s are constrained in the following way:
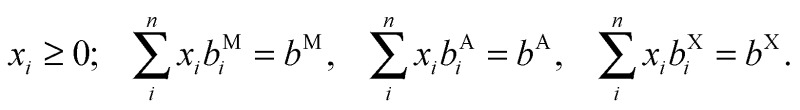
For the disordered 221 alloy and phases with vacancies, the enthalpy is replaced at finite temperature *T* by the Gibbs free energy,

3 *G* = *H* – *TS*_c_,

where *S*_c_ is the configurational entropy, given by4
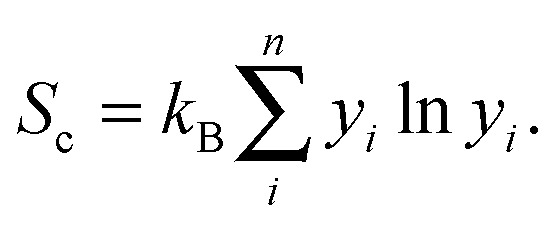
Here, *y*_*i*_ is the concentration of species *i*. A necessary criterion for thermodynamic phase stability is that Δ*H*_cp_ < 0 (or Δ*G*_cp_ < 0).

Lastly, the elastic properties of V_2_Ga_2_C were obtained using the method described by Fast *et al.*,^24^ where five elastic constants *C*_11_, *C*_12_, *C*_13_, *C*_33_, and *C*_44_ are obtained by first distorting the lattice in a stepwise fashion, then calculating the energy of each distortion step, given by5
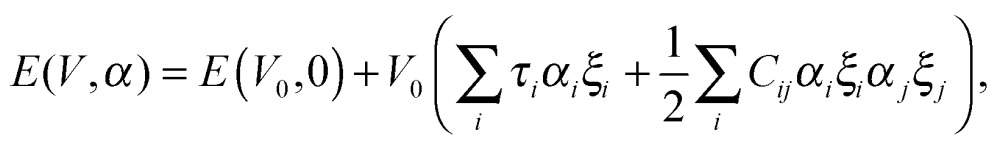
where *E*(*V*_0_,0) is the energy of the unstrained lattice and *α*_*ij*_ are strain parameters, and finally carrying out a quadratic fit on the resulting energy-strain data. Each distortion step here corresponded to a strain of 1% in each direction, with a maximum strain of 2%, *i.e.*, the strain parameters *α*_*i*,*j*_ were allowed to assume the values 0, ±0.01, ±0.02.

## III. Results and discussion

### A. Phase stability

**Fig. 1:**
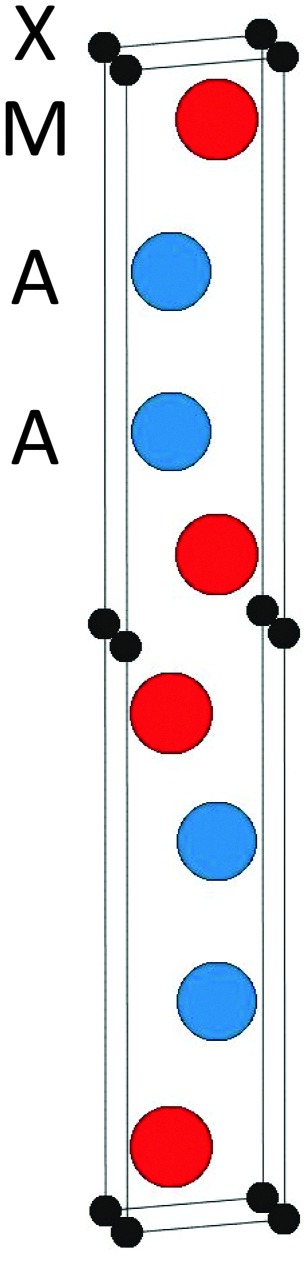
The 221 crystal structure.

The topmost and bottom row of Table 1 contain the formation enthalpies, the lattice parameters, and the respective sets of most competing phases at 0 K for the hypothetical phase V_2_Ga_2_C and the recently synthesized nanolaminate Mo_2_Ga_2_C.^1^ The crystal structure of these phases is shown in Fig. 1. Out of a large number of considered competing phases (see Table S1 in ESI†), the set of most competing phases with respect to V_2_Ga_2_C is found to consist of two experimentally verified phases, the MAX phase V_2_GaC and the rhombohedral binary phase V_8_Ga_41_, and the hypothetical phase V_3_Ga_2_C_2_. The latter belongs to the same space group as V_2_Ga_2_C (*P*6_3_/*mmc*), but contains two V_6_C octahedra between each Ga bilayer instead of one, similar to a MAX phase with a 312 stoichiometry.

**Table 1:** The respective sets of most competing phases, lattice parameters, and formation enthalpies for Mo_2_Ga_2_C, V_2_Ga_2_C, and for ordered and disordered (SQS) (Mo_1–*x*_V_*x*_)_2_Ga_2_C, where *x* = 0.25, 0.5, and 0.75

Phase	Set of most competing phases (0 K)	Lattice parameter (0 K)				Δ*H*_cp_ (meV per atom)	
		Ordered		SQS		Ordered	SQS
		*a* (Å)	*c* (Å)	*a* (Å)	*c* (Å)		
Mo_2_Ga_2_C	Mo_3_Ga, MoGa_4_, MoC	3.064^*a*^	18.153^*a*^			–9^*a*^	
(Mo_0.75_V_0.25_)_2_Ga_2_C	(Mo_0.5_V_0.5_)_2_GaC, Mo_2_Ga_2_C, MoGa_4_, C	3.028	18.256	3.034	18.134	–3.7	13.3
(Mo_0.5_V_0.5_)_2_Ga_2_C	(Mo_0.5_V_0.5_)_2_GaC, (Mo_0.75_V_0.25_)_2_Ga_2_C, V_6_C_5_, MoGa_4_	3.003	18.062	3.001	18.077	18.3	22.4
(Mo_0.25_V_0.75_)_2_Ga_2_C	(Mo_0.25_V_0.75_)_2_GaC, V_2_GaC, V_6_C_5_, MoGa_4_	2.957	18.092	2.975	17.978	13.3	23.8
V_2_Ga_2_C	V_3_Ga_2_C_2_, V_2_GaC, V_8_Ga_41_	2.946	17.861			–0.6	

^*a*^ Ref. 3.

**Fig. 2:**
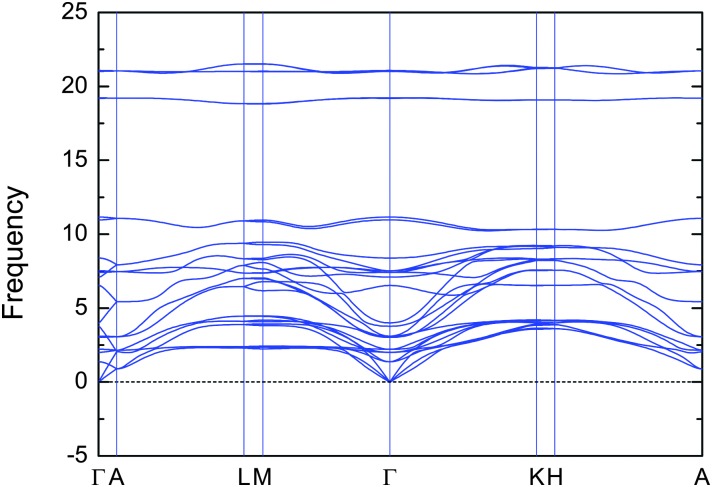
Phonon dispersion in V_2_Ga_2_C for a 4 × 4 × 1 supercell.

Both V_2_Ga_2_C and Mo_2_Ga_2_C fulfill the criterion that Δ*H*_cp_ < 0, although the formation enthalpy of –0.6 meV per atom for V_2_Ga_2_C is close to zero and could therefore hypothetically become positive if temperature dependent effects such as lattice vibrations are also included in the calculations. However, in a recent study we showed that, for the structurally closely related MAX phase Ti_2_AlC, the temperature dependent effects cancel each other out.^25^ Further, as shown in Fig. 2, the phonon dispersion indicates that V_2_Ga_2_C is dynamically stable as there are no imaginary frequencies and thus no imaginary phonon modes present. The phase also fulfills the three criteria for mechanical stability,^26^ which for a hexagonal phase are the inequalities *C*_44_ > 0, *C*_11_ > |*C*_21_|, and (*C*_11_ + *C*_12_)*C*_33_ > 2*C*_13_^2^. The values of the elastic constants can be found in Section IIIC, Table 3.

**Fig. 3:**
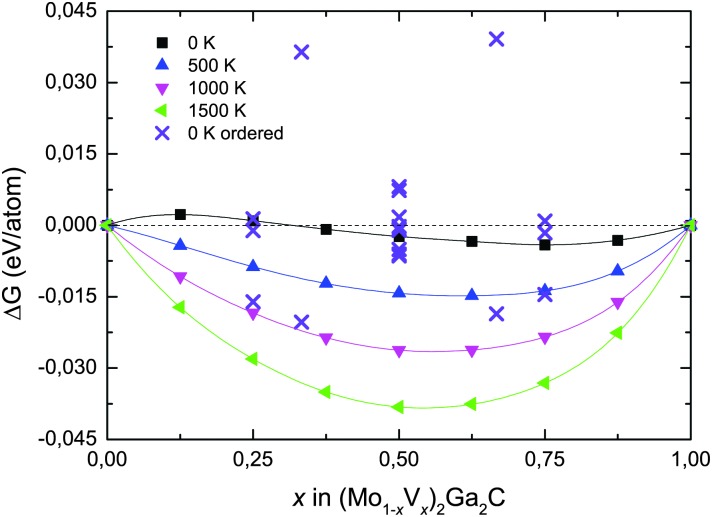
Isostructural Gibbs free energy of formation of (Mo_1–*x*_V_*x*_)_2_Ga_2_C at different temperatures as a function of vanadium concentration *x* with respect to Mo_2_Ga_2_C and V_2_Ga_2_C. Free energies for ordered M sublattice configurations are indicated by crosses.

**Fig. 4:**
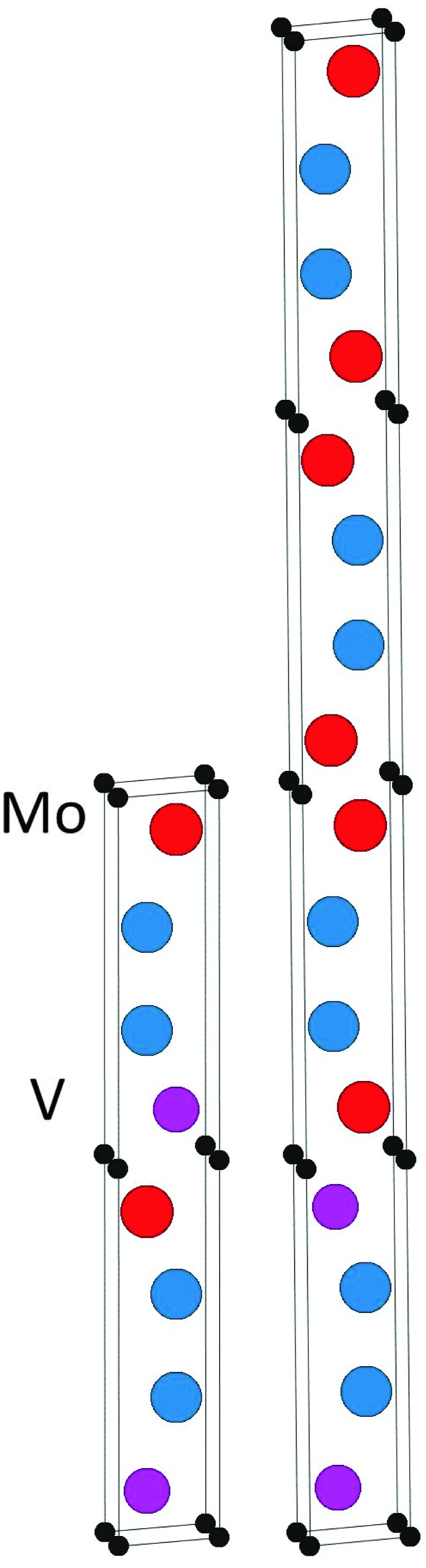
The identified lowest-energy configurations of ordered (Mo_1–*x*_V_*x*_)_2_Ga_2_C for *x* = 0.5 (left) and *x* = 0.25 (right).

We have also investigated the phase stability of the quaternary alloy (Mo_1–*x*_V_*x*_)_2_Ga_2_C. Fig. 3 shows the isostructural Gibbs free energy of formation (as defined in eqn (1), with *G*_221_ substituted for *H*_221_) for chemically disordered (Mo_1–*x*_V_*x*_)_2_Ga_2_C with respect to the two the end members Mo_2_Ga_2_C and V_2_Ga_2_C, for temperatures ranging from 0 to 1500 K. Also included in the figure are the formation enthalpies for chemically ordered (Mo_1–*x*_V_*x*_)_2_Ga_2_C, for a number of different V concentrations *x* on the M sublattice. A common feature of all identified lowest-energy ordered configurations of (Mo_1–*x*_V_*x*_)_2_Ga_2_C is that the Mo and V atoms segregate into single element M layers. As shown in Fig. 4, the preferred M layer sequence for *x* = 0.25 and *x* = 0.5 is 6Mo2V and MoVMoV, respectively. The sequences for the other considered concentrations are 2Mo4V2Mo4V (*x* = 0.33), 4Mo2V4Mo2V (*x* = 0.67), and 2Mo6V (*x* = 0.75). Ordered (Mo_1–*x*_V_*x*_)_2_Ga_2_C outcompetes both a linear combination of Mo_2_Ga_2_C and V_2_Ga_2_C and the disordered alloy for temperatures up to 1000 K. Above these temperatures, disordered (Mo_1–*x*_V_*x*_)_2_Ga_2_C is thermodynamically favored due to a significant configurational entropy contribution to the free energy.

The picture changes significantly when all known competing phases (∼70, see Table S1 in ESI†) in the quaternary Mo–V–Ga–C phase diagram, together with hypothetical phases such as the binary phase MoGa_4_ (based on the existing, related cubic phase CrGa_4_), are included in the calculations. As seen in Table 1 for V concentrations *x* = 0.25, 0.5, and 0.75, the respective sets of most competing phases at 0 K are comprised of ternaries, binaries, and, for *x* = 0.25, pure carbon (graphite), which increases the formation enthalpies for both the ordered and disordered alloy. At 0 K only ordered (Mo_0.75_V_0.25_)_2_Ga_2_C is now stable, with Δ*H*_cp_ = –3.7 meV per atom.

**Fig. 5:**
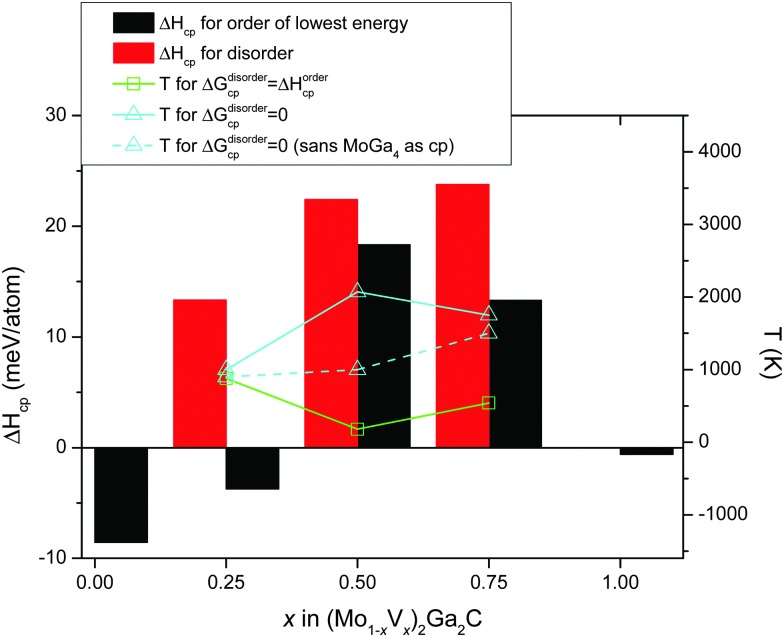
Formation enthalpies of Mo_2_Ga_2_C, V_2_Ga_2_C and of ordered and disordered (Mo_1–*x*_V_*x*_)_2_Ga_2_C (black and red bars, respectively), as a function V concentration with respect to their respective sets of most competing phases. The disordered alloy is stabilized with respect to the ordered one at *T*_disorder_ (green open squares), and with respect to its set of most competing phases at *T*_Δ*G*_cp_=0_ (teal solid line with open triangles). The teal dashed line with open triangles gives *T*_Δ*G*_cp_=0_ if the binary phase MoGa_4_ is excluded as a possible competing phase.

Fig. 5 combines all 0 K data in Table 1 with a superimposed line indicating *T*_disorder_ (green open squares, right hand axis), *i.e.*, the temperature at which the condition Δ*G*disordercp = Δ*H*ordercp is fulfilled and a disordered Mo/V solid solution on the M sublattice becomes energetically favorable (this notation was first introduced in ref. 27). Also indicated in the figure is *T*_Δ*G*_cp_=0_ (solid line, teal open triangles), where Δ*G*disordercp = 0, the temperature at which the disordered alloy first becomes stable with respect to its set of most competing phases. For *x* = 0.5 and 0.75, only disordered (Mo_1–*x*_V_*x*_)_2_Ga_2_C is indicated as potentially stable at elevated temperatures, with *T*_Δ*G*_cp_=0_ ≈ 2100 and 1750 K, respectively. For *x* = 0.25, on the other hand, both ordered and disordered (Mo_1–*x*_V_*x*_)_2_Ga_2_C is stable, with *T*_disorder_ ≈ 880 K, and *T*_Δ*G*_cp_=0_ ≈ 1000 K. Since common bulk synthesis temperatures are 1200–1600 °C (1473–1873 K), these results thus suggest that synthesis of the disordered quaternary alloy (Mo_1–*x*_V_*x*_)_2_Ga_2_C should at least be possible for *x* ≤ 0.25 and *x* ≥ 0.75. For the former concentration range, it might be possible to synthesize an ordered configuration at temperatures below 880 K.

It should be noted that for all three V concentrations, MoGa_4_ (a phase not to be confused with Mo_6_Ga_31_, which has almost the same stoichiometry, but a different structure) is one of the most competing phases not only at 0 K, but also at and above *T*_Δ*G*_cp_=0_. This is a binary phase which has, to the best of our knowledge, not yet been experimentally verified. This might be because of a lack of trying, or because it does not form readily. If the latter is the case, the experimental window for synthesizing disordered (Mo_1–*x*_V_*x*_)_2_Ga_2_C (which would then be a metastable phase) expands significantly, with possible synthesis temperatures for *x* = 0.25, 0.5, and 0.75 starting at around 900, 1000, and 1500 K, respectively. These temperatures are indicated by the teal dashed line in Fig. 5.

Table 1 shows that the *a* and *c* lattice parameters decrease more or less linearly with a decreasing amount of Mo. The *a* parameter obeys Vegard's law quite closely, while this is not apparent for the *c* parameter. Finally, the differences between corresponding parameters for the ordered and the disordered alloy are quite small.

### B. Electronic properties of V_2_Ga_2_C

**Fig. 6:**
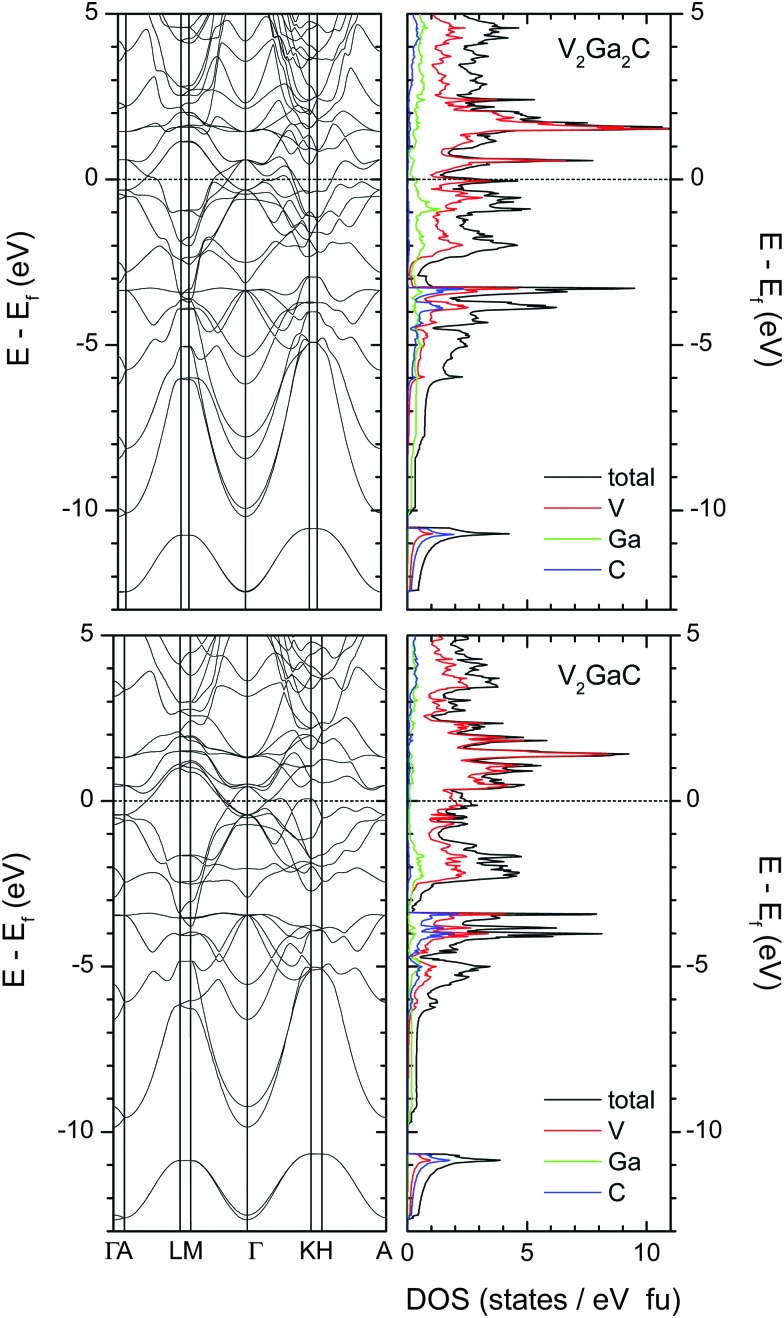
Electronic band structure and total and atomic densities of states for V_2_Ga_2_C (top left and right panels) and V_2_GaC (bottom panels). The dashed lines indicate the location of the Fermi level.

Fig. 6 shows the electronic band structure and the total and atomic densities of states (tDOS and aDOS, respectively) for V_2_Ga_2_C and V_2_GaC. The band structure of V_2_Ga_2_C exhibits considerable anisotropy around the Fermi level, with several band crossings in the *A*–*L*, *M*–*Γ*, *Γ*–*K*, and *H*–*A* reciprocal space directions, but none in the *Γ*–*A*, *L*–*M*, and *K*–*H* directions. Band structure anisotropy, which together with electron–phonon coupling anisotropy^28^ is often an indicator of anisotropic conductive properties, is a common feature of MAX phases, including V_2_GaC and the 221 phase Mo_2_Ga_2_C.^3,29,30^

Qualitatively, the tDOS and aDOS for V_2_Ga_2_C are very similar compared to V_2_GaC. The states around *E*_F_ in both phases are mostly dominated by V electrons (more specifically V 3d electrons). Evidence of V–C and weak V–Ga bonding is seen at around –3.5 eV, where there is overlap between the V, Ga, and C peaks; slightly stronger V–Ga bonds should be expected in the intervals from –2 eV to –1 eV, where more Ga states are present in both V_2_Ga_2_C and V_2_GaC.

A Bader charge analysis has been carried out in order to investigate the charge transfer in V_2_Ga_2_C, which is a consequence of the differences in electronegativity between the constituting elements (1.6 for V and Ga; 2.5 for C).^31^ As seen in Table 2, a charge of 0.5 *e* is transferred from the [V_2_C] blocks to the Ga layers, whereas within the [V_2_C] blocks, 1.73 *e* is transferred from V_2_ to C.

**Table 2:** Partial charge of the M, A, and X atoms in V_2_Ga_2_C, V_2_GaC, Mo_2_Ga_2_C, and Mo_2_GaC

Phase	Partial charge			
	V	Mo	Ga	C
V_2_Ga_2_C	+1.11		–0.25	–1.73
V_2_GaC	+1.13		–0.55	–1.72
Mo_2_Ga_2_C		+0.74	–0.04	–1.38
Mo_2_GaC		+0.78	–0.17	–1.39

Also listed in Table 2 are the partial charges of the M, A, and X atoms in V_2_GaC, Mo_2_Ga_2_C, and Mo_2_GaC. The [M_2_C] → Ga charge transfer is smaller in the 221 phases than in the corresponding MAX phases, as the former contain two Ga atoms per [M_2_C] block instead of one. However, this fact alone cannot account for the observed difference, since the transferred charge per Ga atom in the 221 phases is less than half the amount of charge transferred in the MAX phases. For V_2_Ga_2_C, the discrepancy is 0.05 *e* (0.5 *e vs.* 0.55 *e*), while it is 0.09 *e* for Mo_2_Ga_2_C (0.08 *e vs.* 0.17 *e*). These discrepancies are likely caused by differences in the electronic structure, and they may suggest that the ionic components of the bonds between the [M_2_C] blocks and the Ga layers are slightly weaker in the 221 phases than in the corresponding MAX phases.

### C. Elastic properties of V_2_Ga_2_C

The Voigt bulk (*B*_V_) and Voigt shear modulus (*G*_V_) can be calculated from the five elastic constants *C*_11_, *C*_12_, *C*_13_, *C*_33_, and *C*_44_ discussed in Section II. The moduli are related to these constants as6
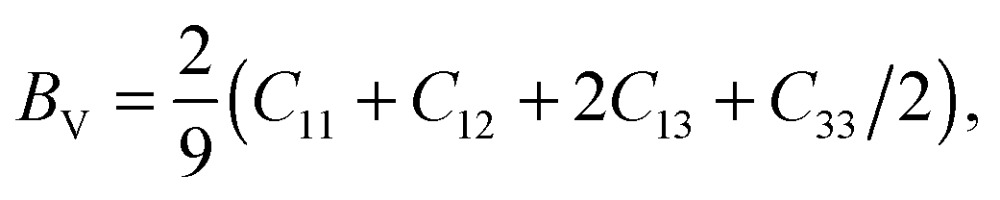
and

7 *G*_V_ = 115(2*C*_11_ + *C*_33_ – *C*_12_ – 2*C*_13_) + 15(2*C*_44_ + 12(*C*_11_ – *C*_12_)).

From *B*_V_ and *G*_V_, Young's modulus (*E*) and Poisson's ratio (*ν*) can be calculated:8
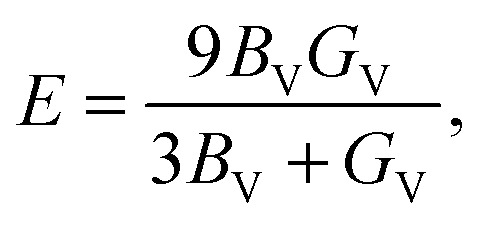

**Fig. 7:**
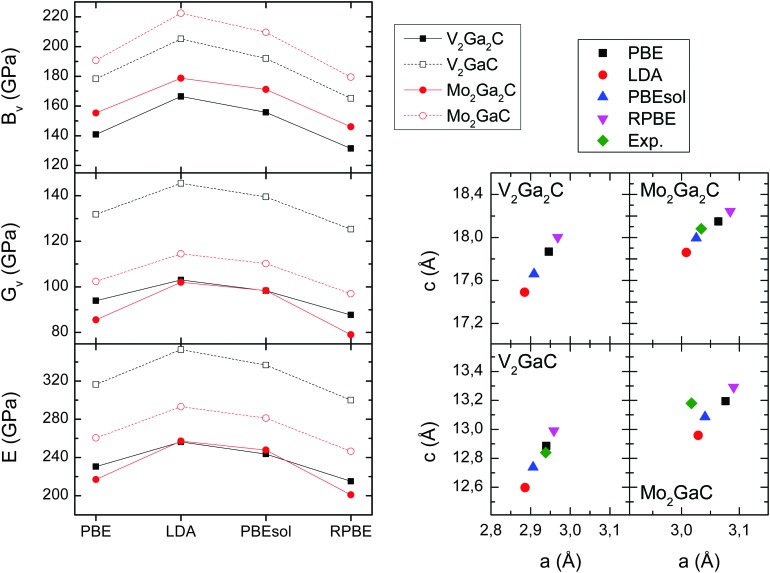
Left panels: Bulk (*B*_V_), shear (*G*_V_), and Young's moduli (*E*) for V_2_Ga_2_C, V_2_GaC, Mo_2_Ga_2_C, and Mo_2_GaC for four different exchange–correlation functionals. Right panels: Lattice parameters for the same four exchange–correlation functionals, and experimentally determined lattice parameters taken from ref. 1 (Mo_2_Ga_2_C) and ref. 34 (V_2_GaC, Mo_2_GaC).

9
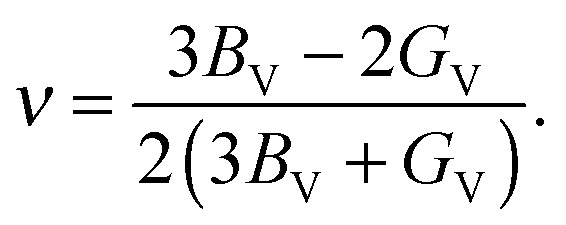
 The three moduli given by eqn (6)–(8) are shown for V_2_Ga_2_C in the three left panels of Fig. 7, together with the corresponding theoretical data for V_2_GaC, Mo_2_Ga_2_C, and Mo_2_GaC, for comparison. In order to bound the moduli, we have used four different exchange–correlation functionals: PBE, LDA, and the two revised PBE functionals PBEsol and RPBE.^32,33^ The same functionals have been used to calculate the lattice parameters for all phases, as seen in the four right panels of Fig. 7. The RPBE functional yields the largest lattice parameters and thus sets a lower bound for the moduli, while the LDA sets an upper bound as it yields the smallest parameters. As is also seen in the figure, the experimentally determined lattice parameters for V_2_GaC, Mo_2_Ga_2_C, and Mo_2_GaC closely match those from the PBE and PBEsol calculations. This suggests that, if V_2_Ga_2_C is synthesized, the lattice parameters will likely agree well with both the PBE and PBEsol calculations. We thus expect that these two functionals give the most accurate estimates of the elastic moduli out of the four functionals tested here.

In Table 3 we list the five elastic constants, the three moduli, and the Poisson's ratios (eqn (9)) for V_2_Ga_2_C, V_2_GaC, Mo_2_Ga_2_C, and Mo_2_GaC, all obtained from calculations using the PBE exchange–correlation functional. As is seen in the table, V_2_Ga_2_C has similar moduli compared to Mo_2_Ga_2_C, but they are significantly lower (20–30%) than for V_2_GaC. A decrease in the moduli is also observed for Mo_2_Ga_2_C compared to Mo_2_GaC, as first reported in ref. 3.

**Table 3:** Calculated elastic constants *C*_*ij*_ (GPa), Voigt bulk *B*_V_ (GPa) and Voigt shear moduli *G*_V_ (GPa), Young's moduli *E* (GPa), and Poisson's ratios *ν* for V_2_Ga_2_C, V_2_GaC, Mo_2_Ga_2_C, and Mo_2_GaC. Data for the two Mo phases has been taken from ref. 3. All values have been calculated using the PBE exchange–correlation functional

Phase	*C*_11_	*C*_12_	*C*_13_	*C*_33_	*C*_44_	*B*_V_	*G*_V_	*E*	*ν*
V_2_Ga_2_C	262	64	69	345	76	141	94	230	0.23
V_2_GaC	331	70	116	298	139	178	132	316	0.21
Mo_2_Ga_2_C	244	62	108	341	78	154	86	218	0.26
Mo_2_GaC	294	96	161	289	126	190	101	257	0.28

At 0.23, Poisson's ratio for V_2_Ga_2_C is 10% greater than for V_2_GaC, and about 12% smaller than for Mo_2_Ga_2_C.

The differences in the elastic properties of the 221 phases compared to the MAX phases can be attributed to the additional Ga layers in the former. Part of the explanation might be a weaker ionic component of the bonds between the [M_2_C] blocks and Ga layers compared to the MAX phases, which was discussed in Section IIIB. Likely to be more significant, however, are the Ga–Ga bonds along the *c* axis in the 221 phases, which should be mainly metallic in character and presumably rather weak compared to the vertical V–Ga, V–C, and V–V bonds.

## IV. Conclusions

We have calculated the phase stability of V_2_Ga_2_C and of the related alloy (Mo_1–*x*_V_*x*_)_2_Ga_2_C for three different V concentrations: *x* = 0.25, 0.5, and 0.75. The formation enthalpy of V_2_Ga_2_C with respect to its set of most competing phases is –0.6 meV per atom, which suggests that it is thermodynamically stable. Furthermore, V_2_Ga_2_C was found to be dynamically as well as mechanically stable.

For (Mo_1–*x*_V_*x*_)_2_Ga_2_C and *x* ≤ 0.25, phase stability is indicated both for an ordered and a disordered configuration on the M sublattice, where the latter is stabilized at around 1000 K. Phase stability is further indicated for disordered (Mo_1–*x*_V_*x*_)_2_Ga_2_C for *x* = 0.5 and *x* ≥ 0.75, at temperatures of about 2100 and 1750 K, respectively.

We have also investigated the electronic and elastic properties of V_2_Ga_2_C. The layered nature of the crystal structure is reflected in the electronic band structure in the form of a distinct anisotropy, which is a possible indicator of anisotropic conductivity. The calculated Voigt bulk modulus is 141 GPa, the Voigt shear modulus 94 GPa, and Young's modulus 230 GPa, which are lower values than for the corresponding MAX phase V_2_GaC. A Bader analysis shows significant charge transfer within the [V_2_C] blocks and a smaller transfer between the [V_2_C] blocks and the Ga layers in both V_2_Ga_2_C and the MAX phase counterpart V_2_GaC. The differences in the elastic moduli between V_2_Ga_2_C and V_2_GaC might be partly explained by a smaller [V_2_C] → Ga transfer in V_2_Ga_2_C, but we speculate that the most important factor is weak interlayer bonding between the Ga layers in this phase.
