# Supplementary figures and images for: Phase stability of the nanolaminates V2Ga2C and (Mo1–xVx)2Ga2C from first-principles calculations
Source: Phys Chem Chem Phys. 2016 Apr 20;18(18):12682–8. doi: 10.1039/c6cp00802j (PMC5066482; doi:10.1039/c6cp00802j)

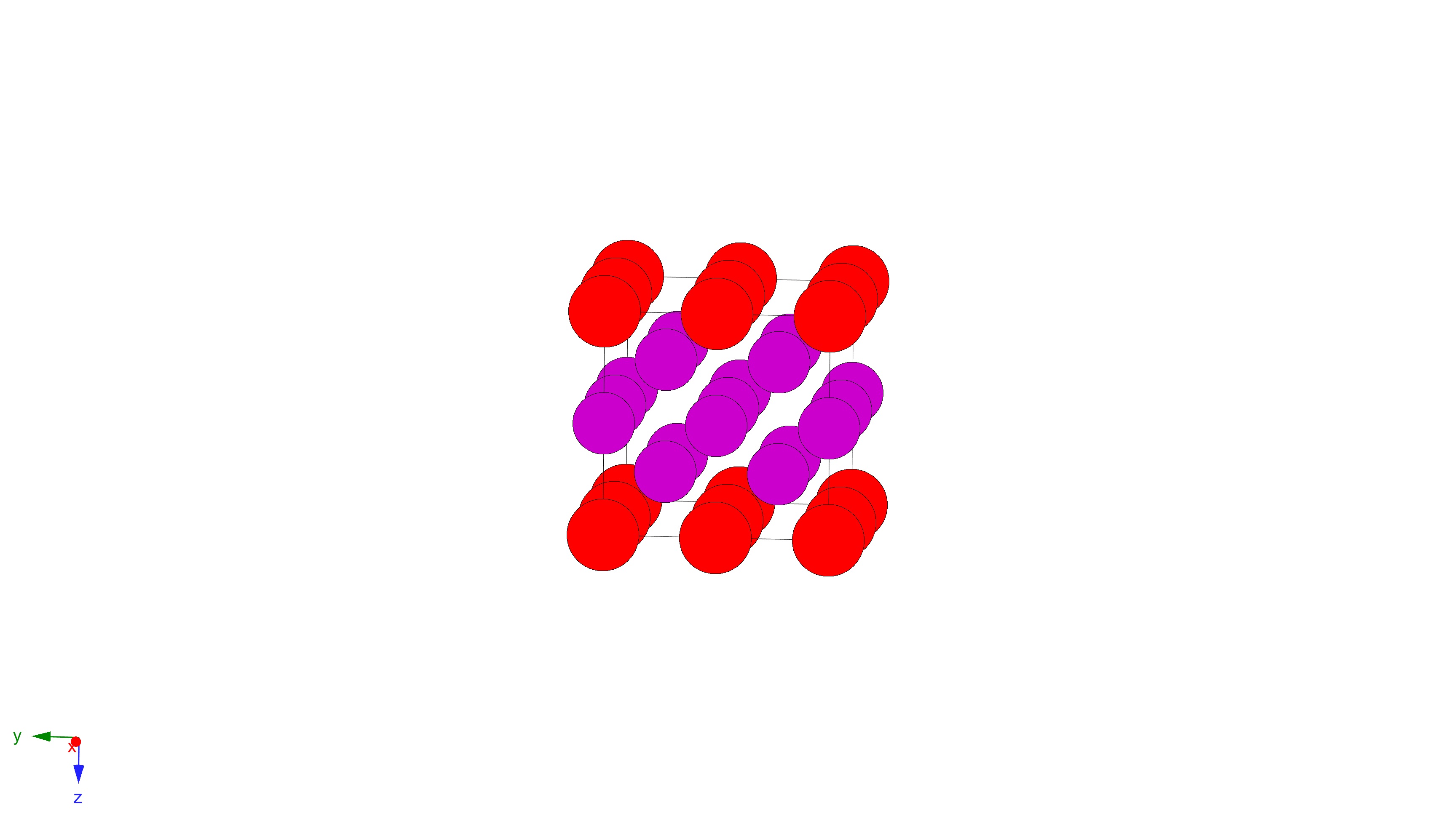

Supplement: Supplementary file 1 [file CP-018-C6CP00802J-s001.zip › mov_alloy_figures/mov/25mo/1a.jpg]

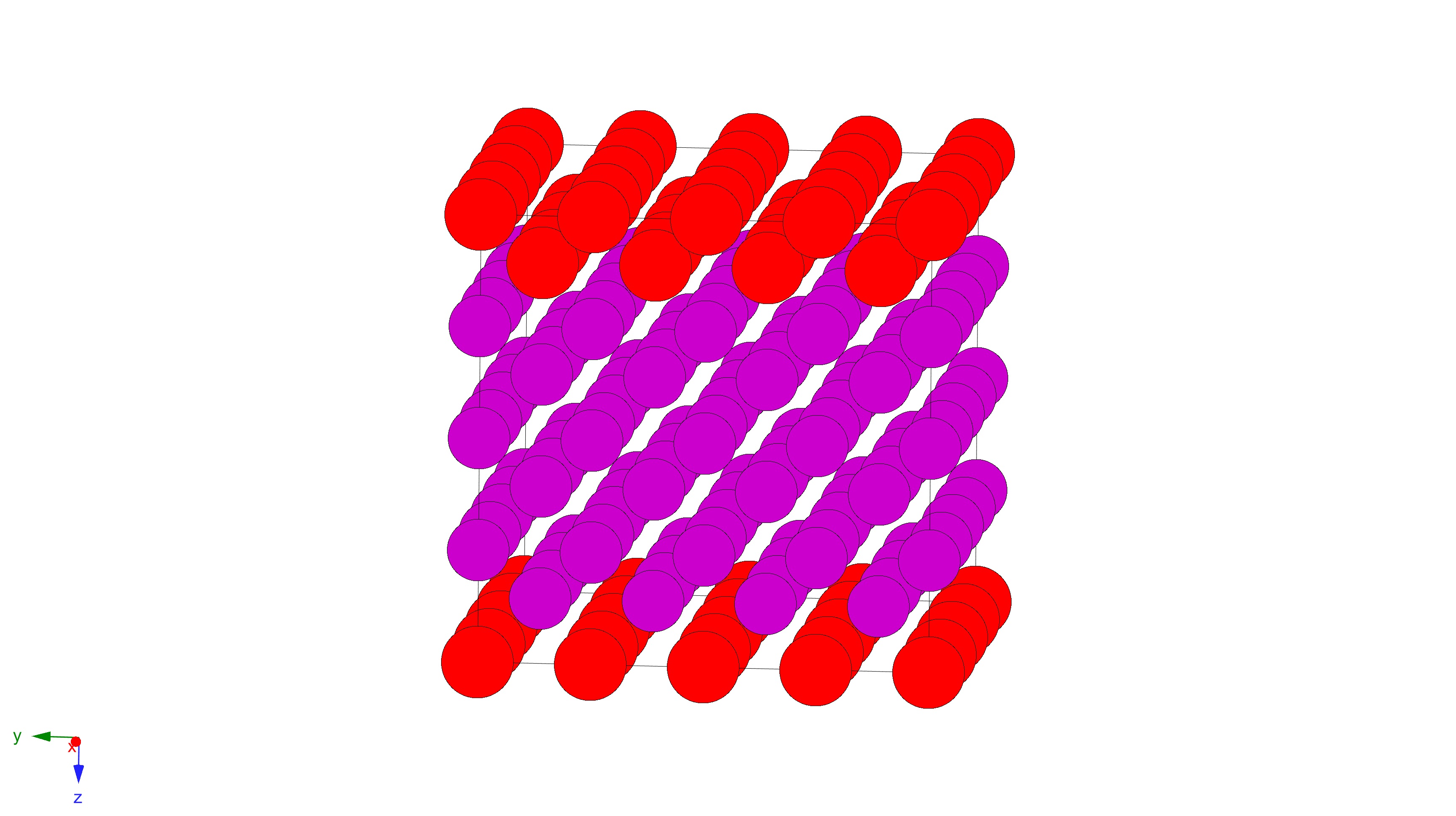

Supplement: Supplementary file 1 [file CP-018-C6CP00802J-s001.zip › mov_alloy_figures/mov/25mo/1b.jpg]

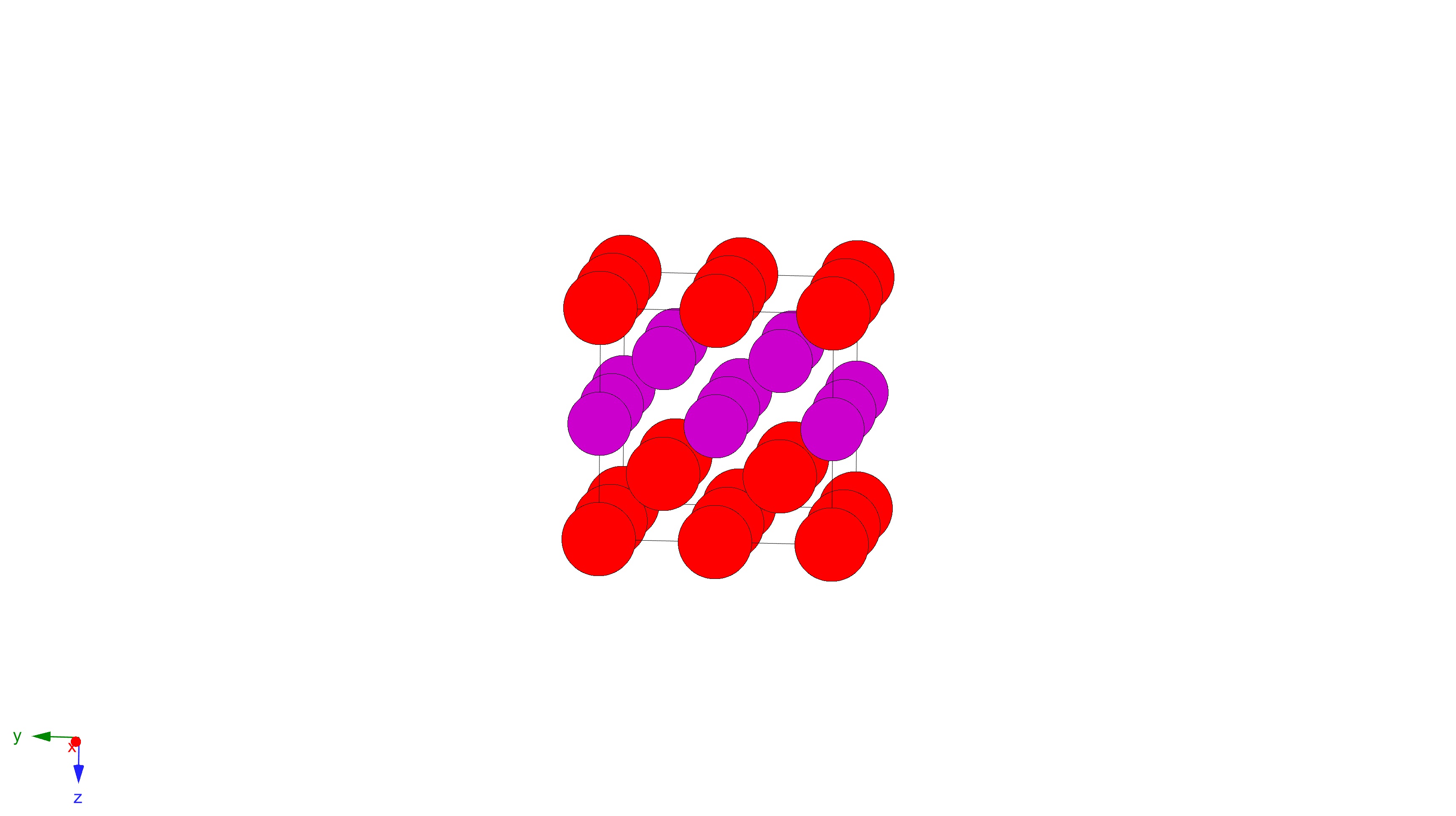

Supplement: Supplementary file 1 [file CP-018-C6CP00802J-s001.zip › mov_alloy_figures/mov/50mo/1c.jpg]

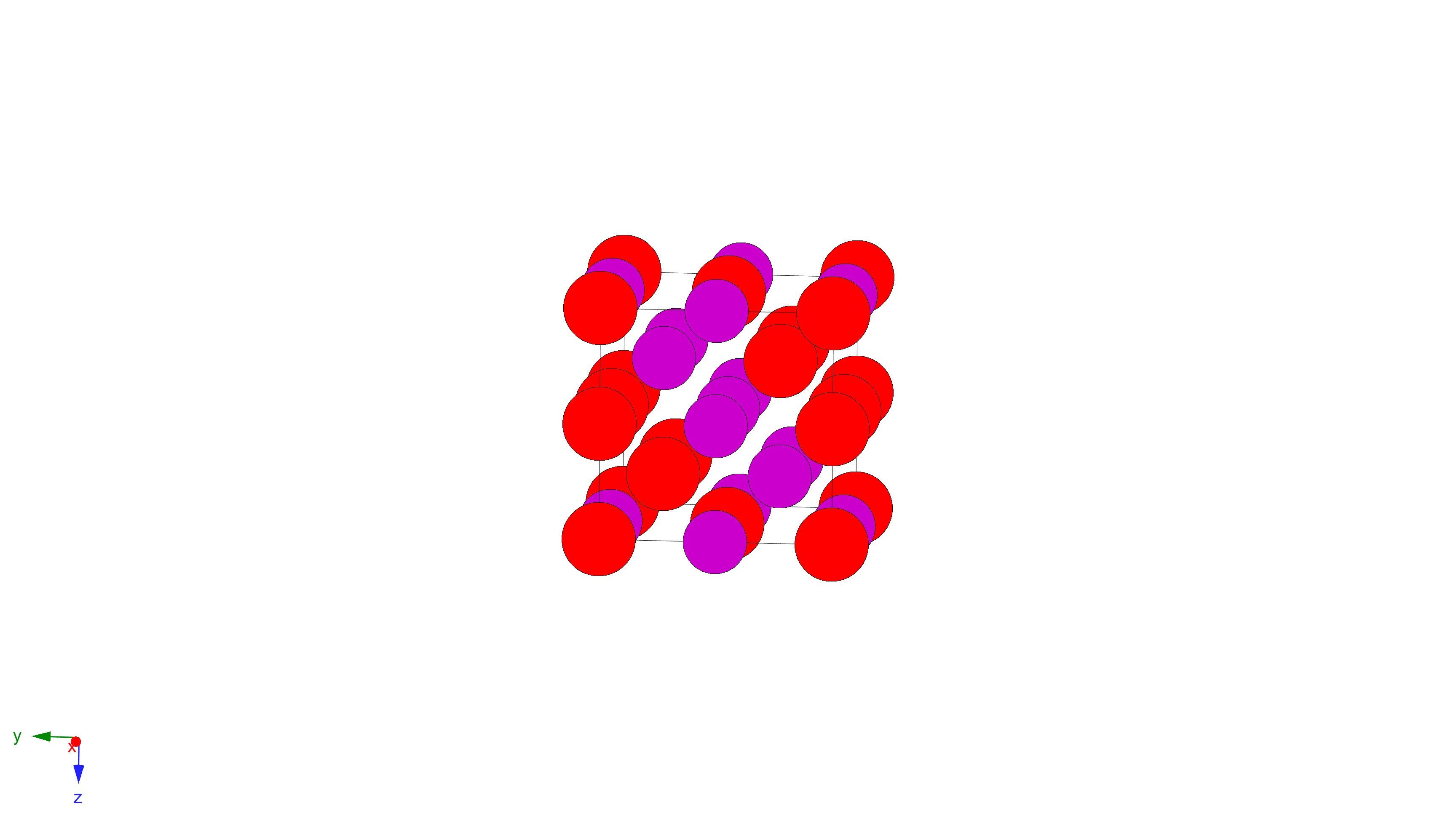

Supplement: Supplementary file 1 [file CP-018-C6CP00802J-s001.zip › mov_alloy_figures/mov/50mo/1d.jpg]

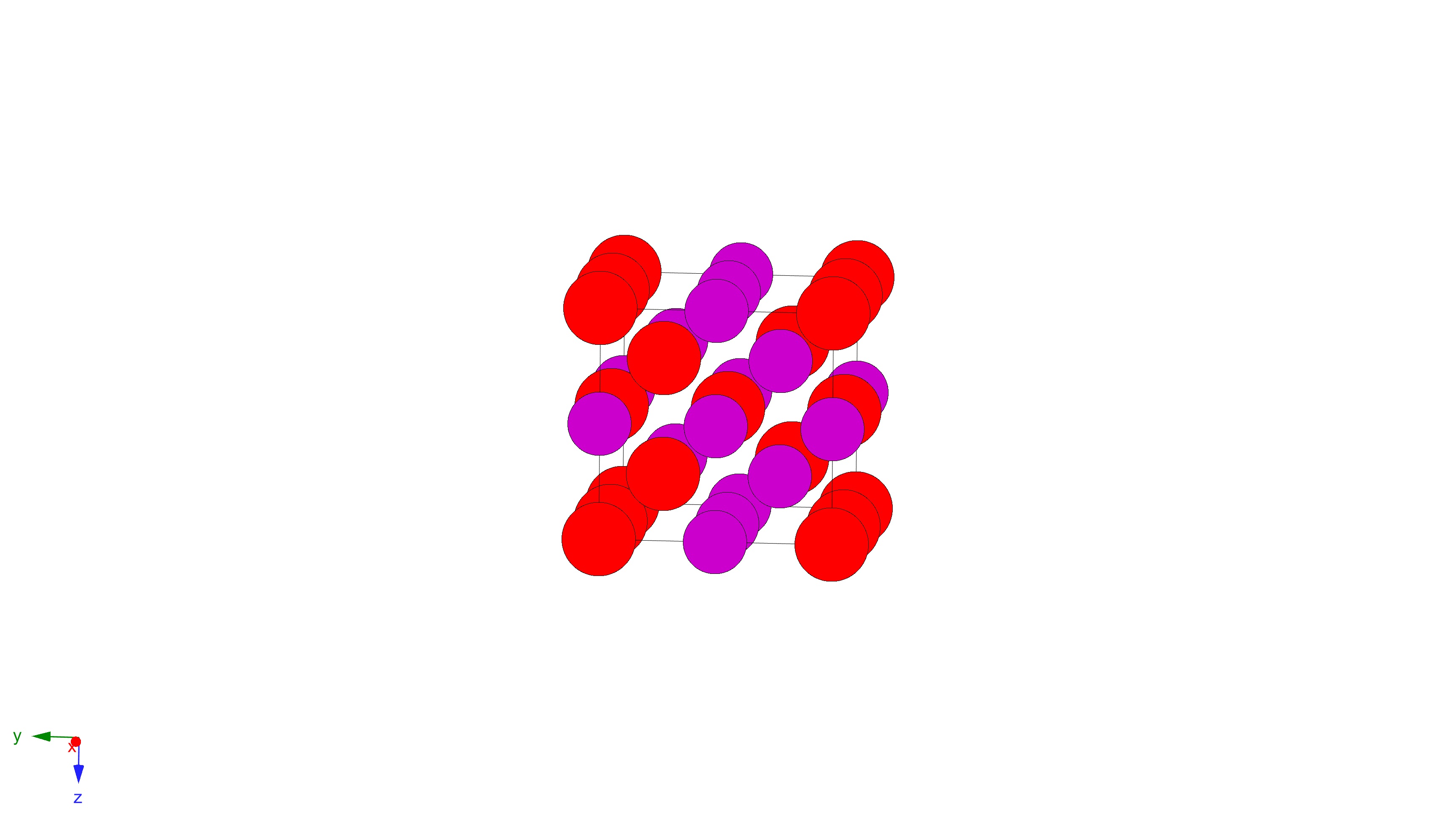

Supplement: Supplementary file 1 [file CP-018-C6CP00802J-s001.zip › mov_alloy_figures/mov/50mo/1e.jpg]

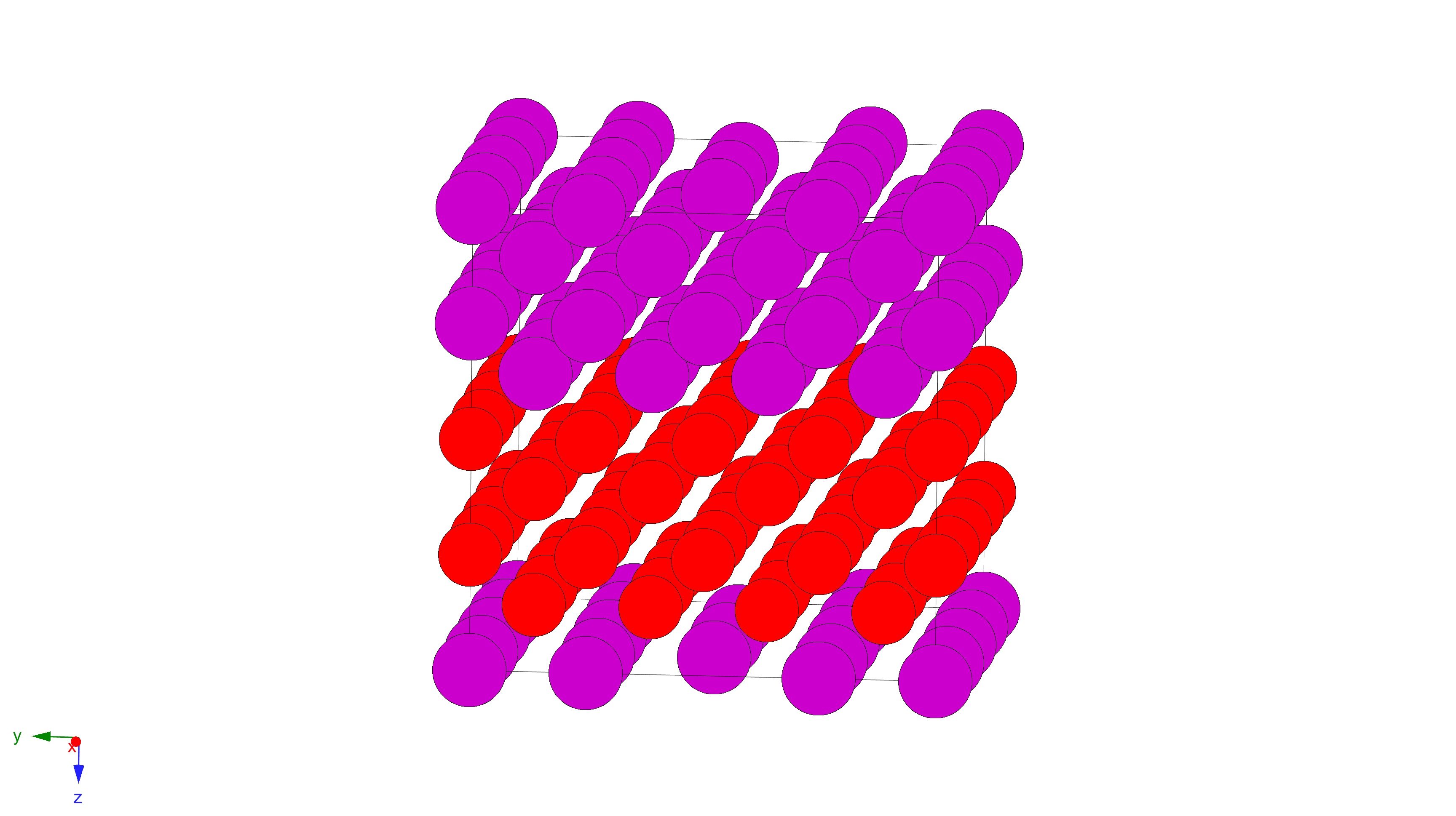

Supplement: Supplementary file 1 [file CP-018-C6CP00802J-s001.zip › mov_alloy_figures/mov/50mo/1f.jpg]

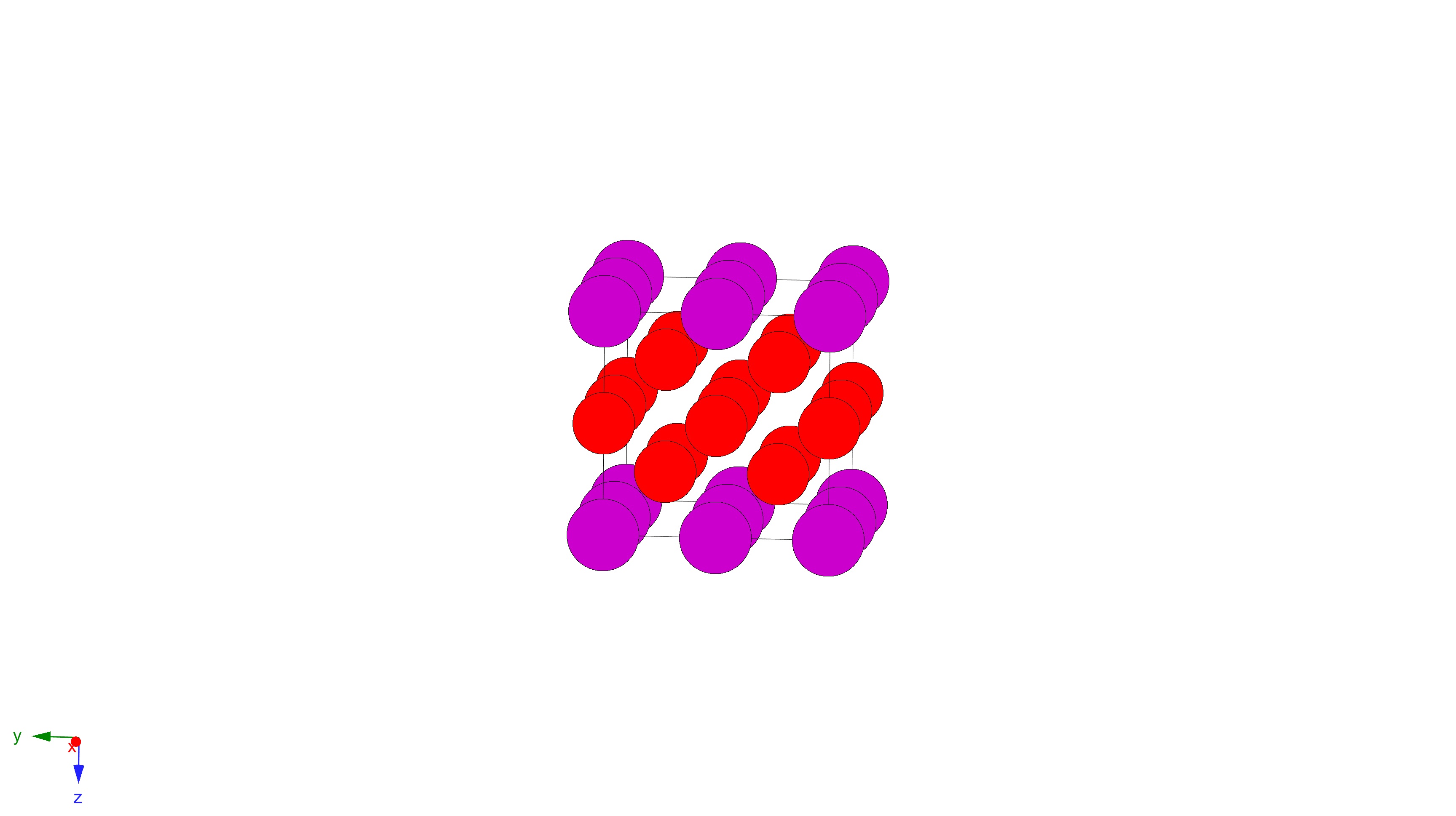

Supplement: Supplementary file 1 [file CP-018-C6CP00802J-s001.zip › mov_alloy_figures/mov/75mo/1g.jpg]

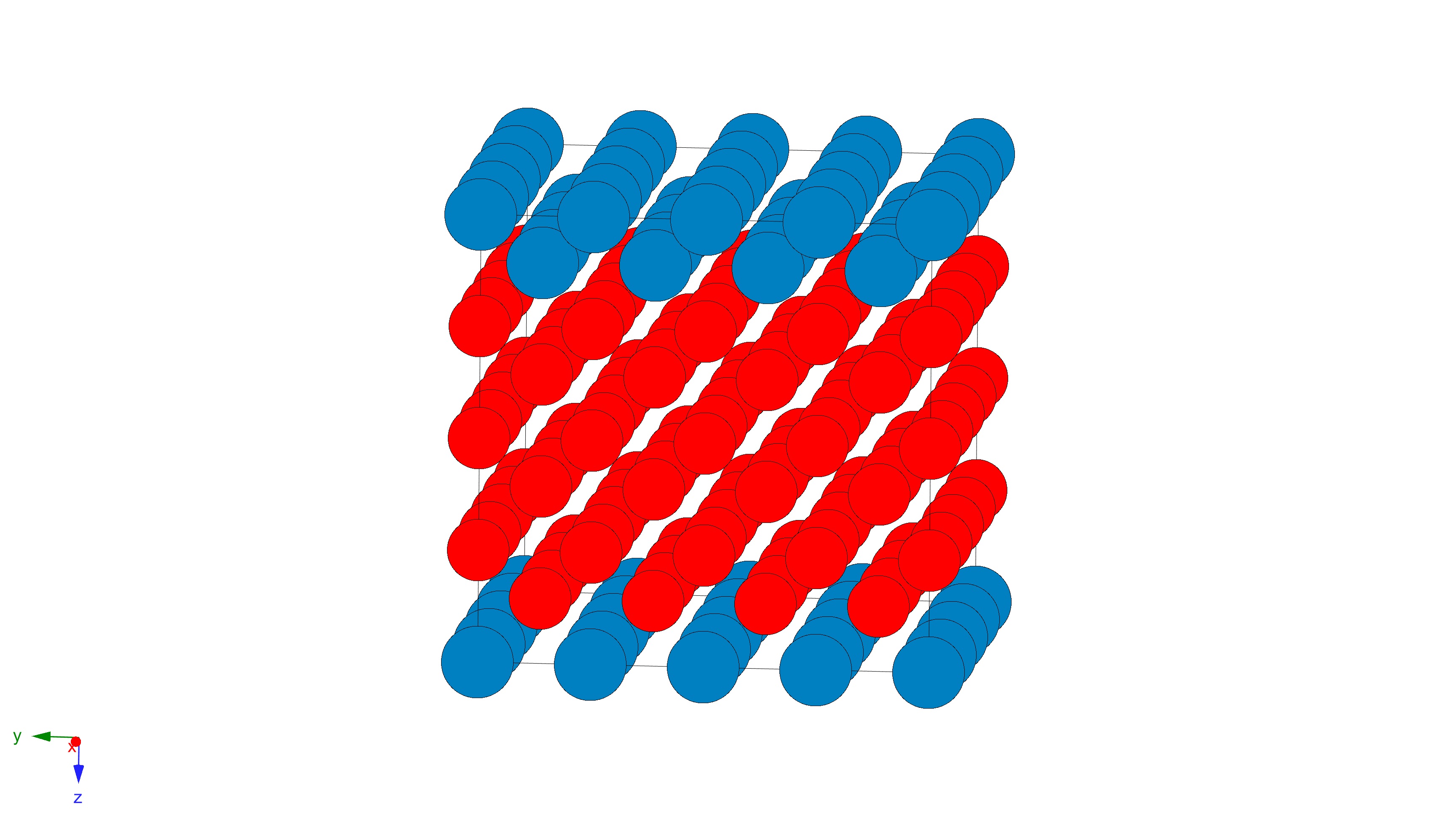

Supplement: Supplementary file 1 [file CP-018-C6CP00802J-s001.zip › mov_alloy_figures/mov/75mo/1h.jpg]

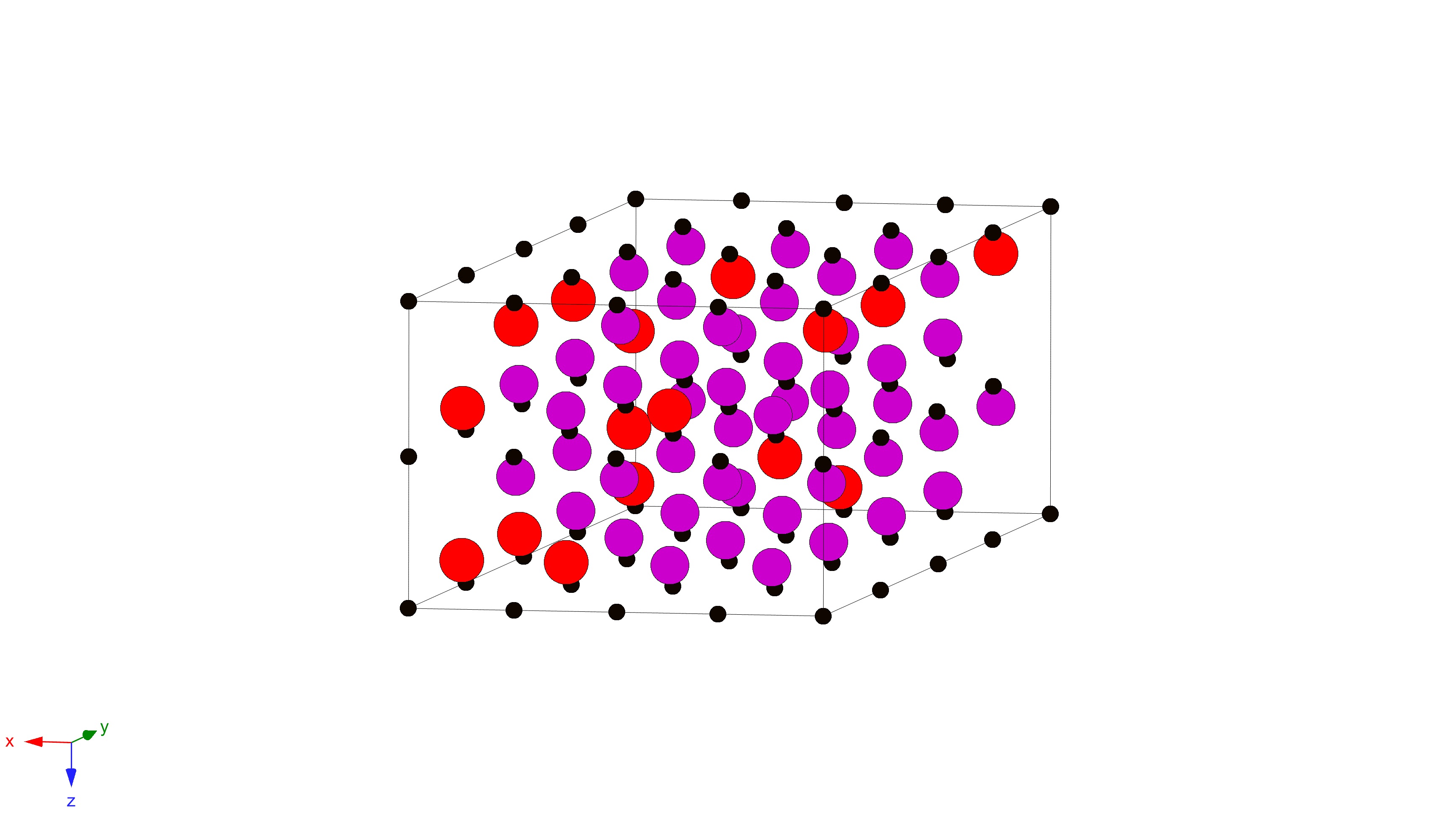

Supplement: Supplementary file 1 [file CP-018-C6CP00802J-s001.zip › mov_alloy_figures/mov2c/25mo/5a.jpg]

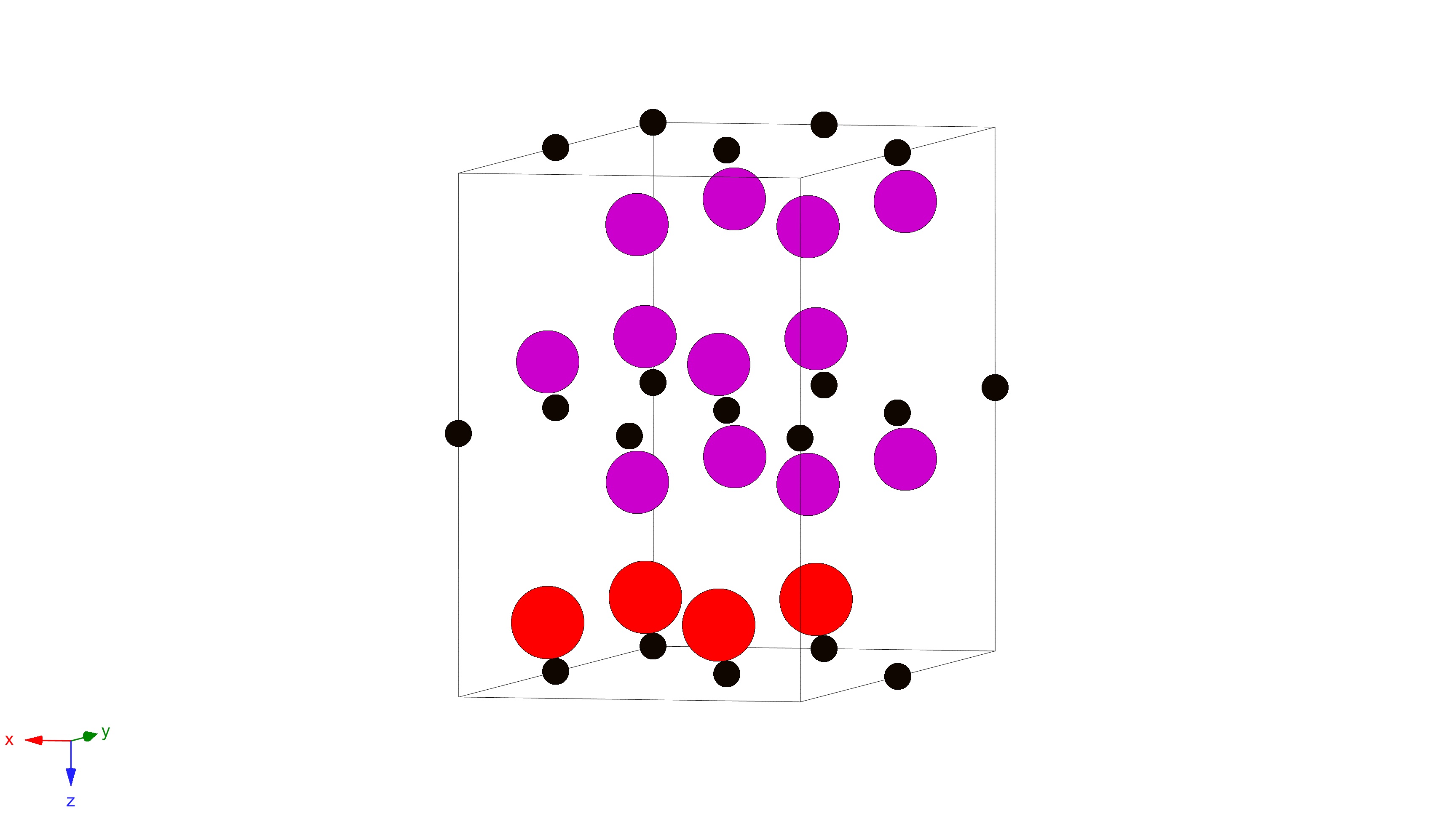

Supplement: Supplementary file 1 [file CP-018-C6CP00802J-s001.zip › mov_alloy_figures/mov2c/25mo/5b.jpg]

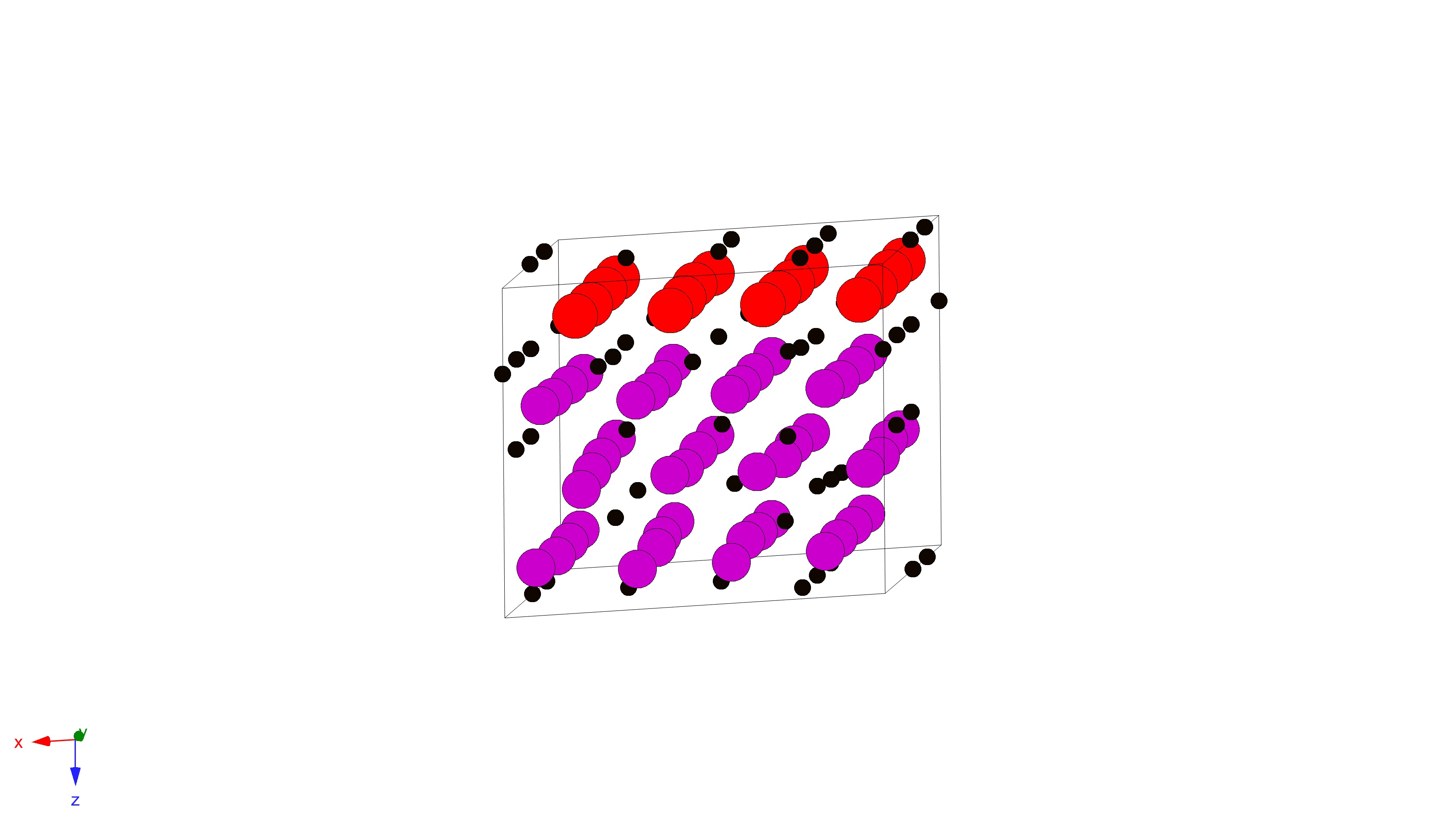

Supplement: Supplementary file 1 [file CP-018-C6CP00802J-s001.zip › mov_alloy_figures/mov2c/25mo/5c.jpg]

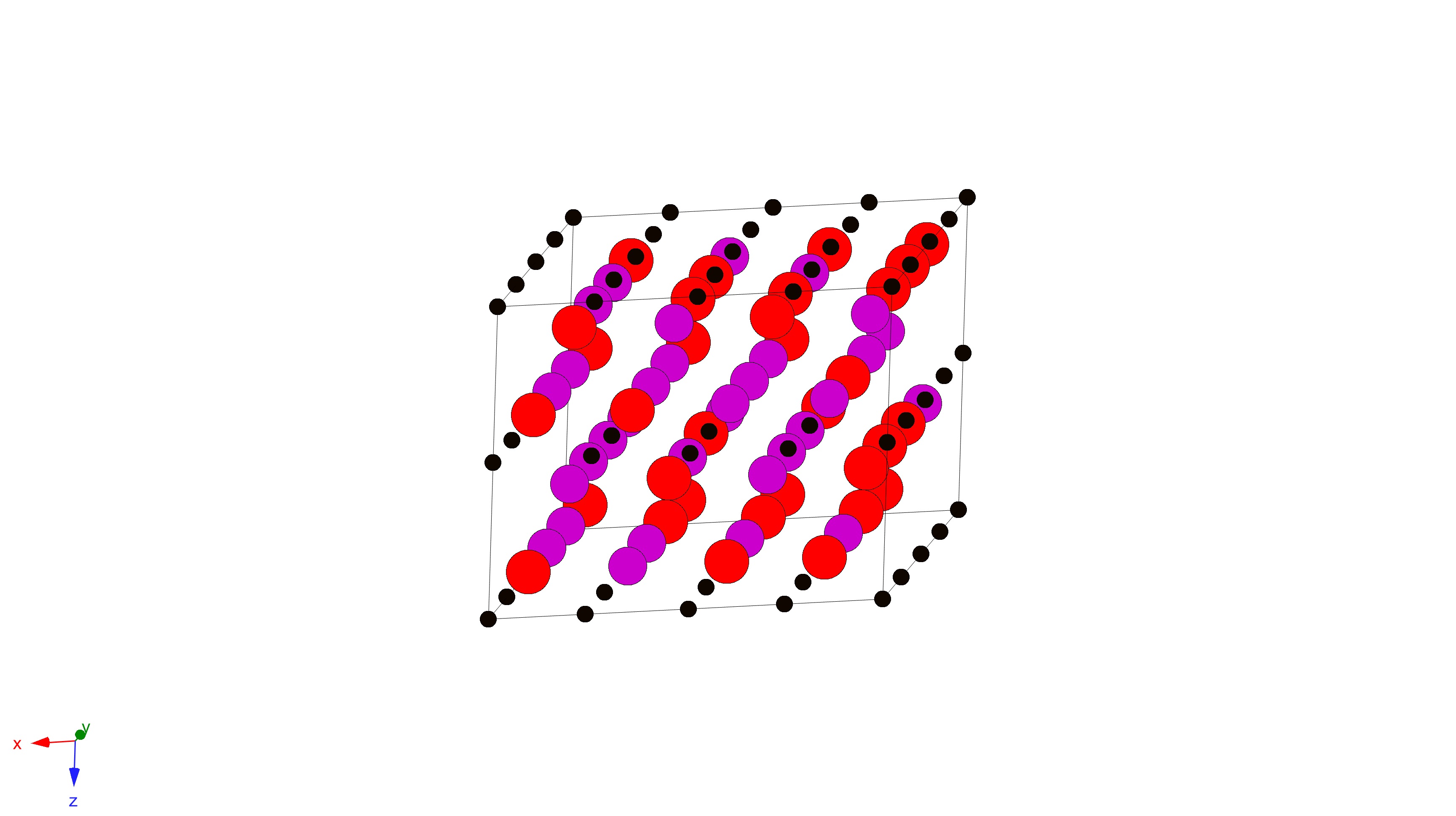

Supplement: Supplementary file 1 [file CP-018-C6CP00802J-s001.zip › mov_alloy_figures/mov2c/50mo/5d.jpg]

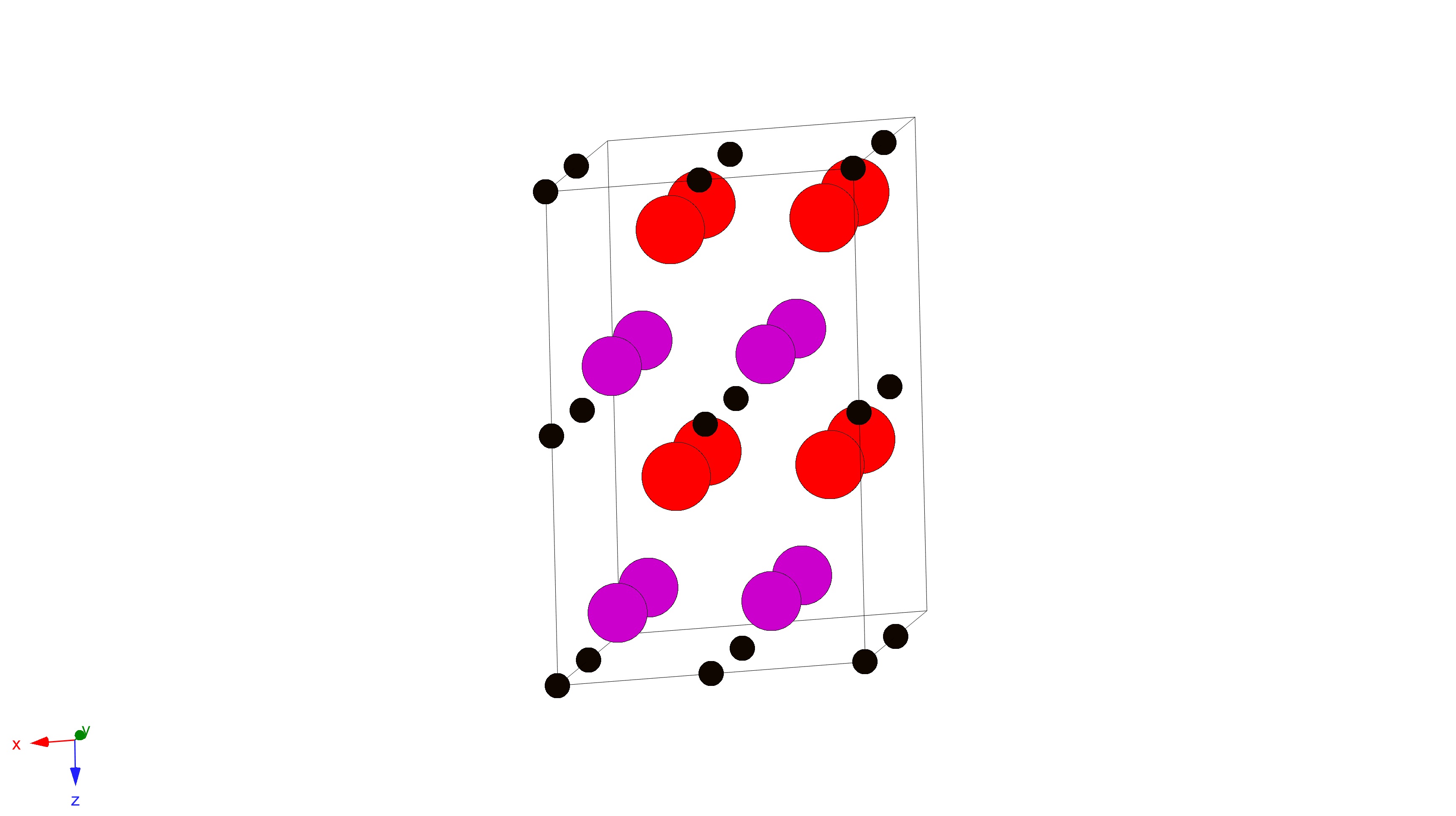

Supplement: Supplementary file 1 [file CP-018-C6CP00802J-s001.zip › mov_alloy_figures/mov2c/50mo/5e.jpg]

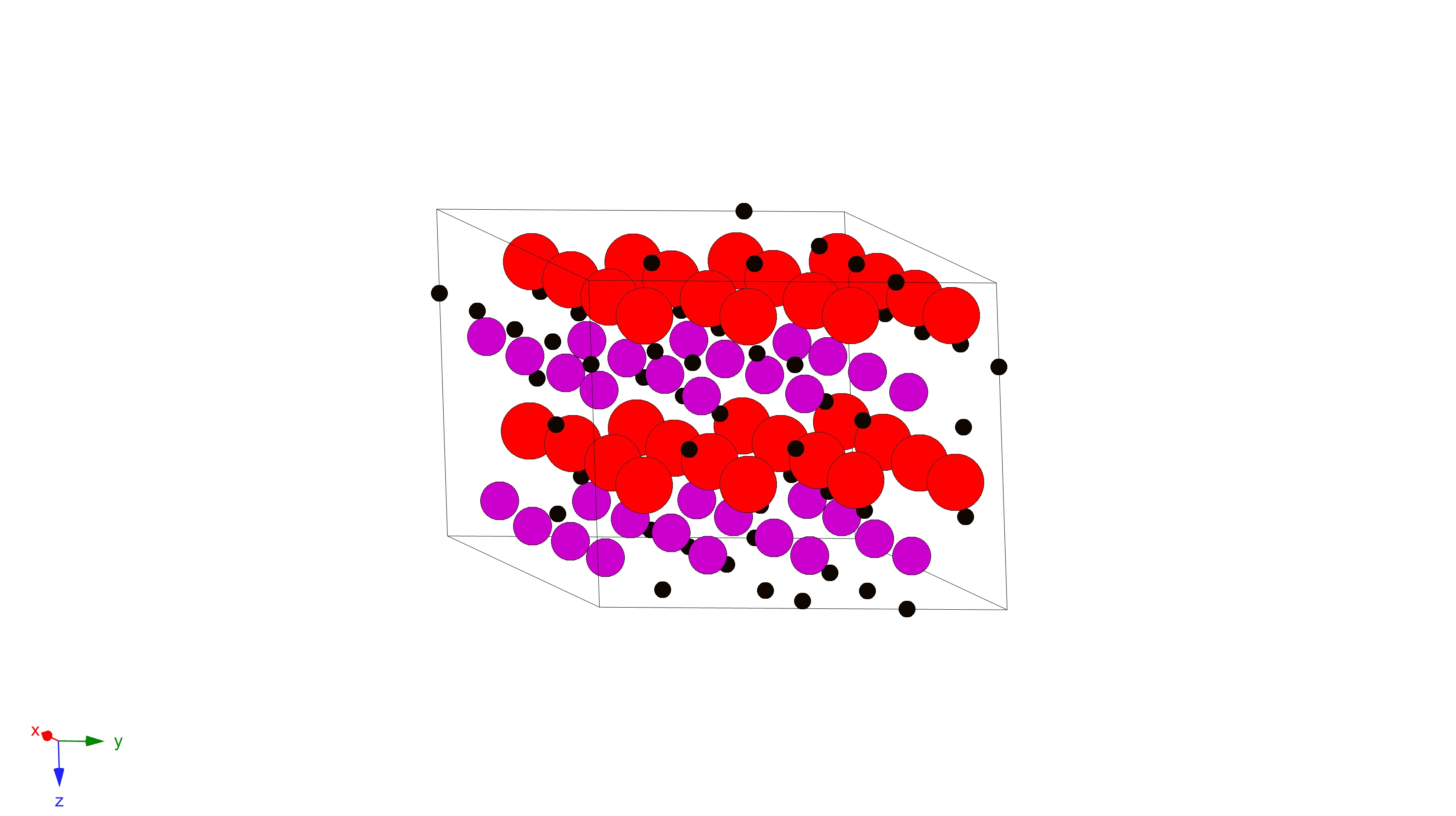

Supplement: Supplementary file 1 [file CP-018-C6CP00802J-s001.zip › mov_alloy_figures/mov2c/50mo/5f.jpg]

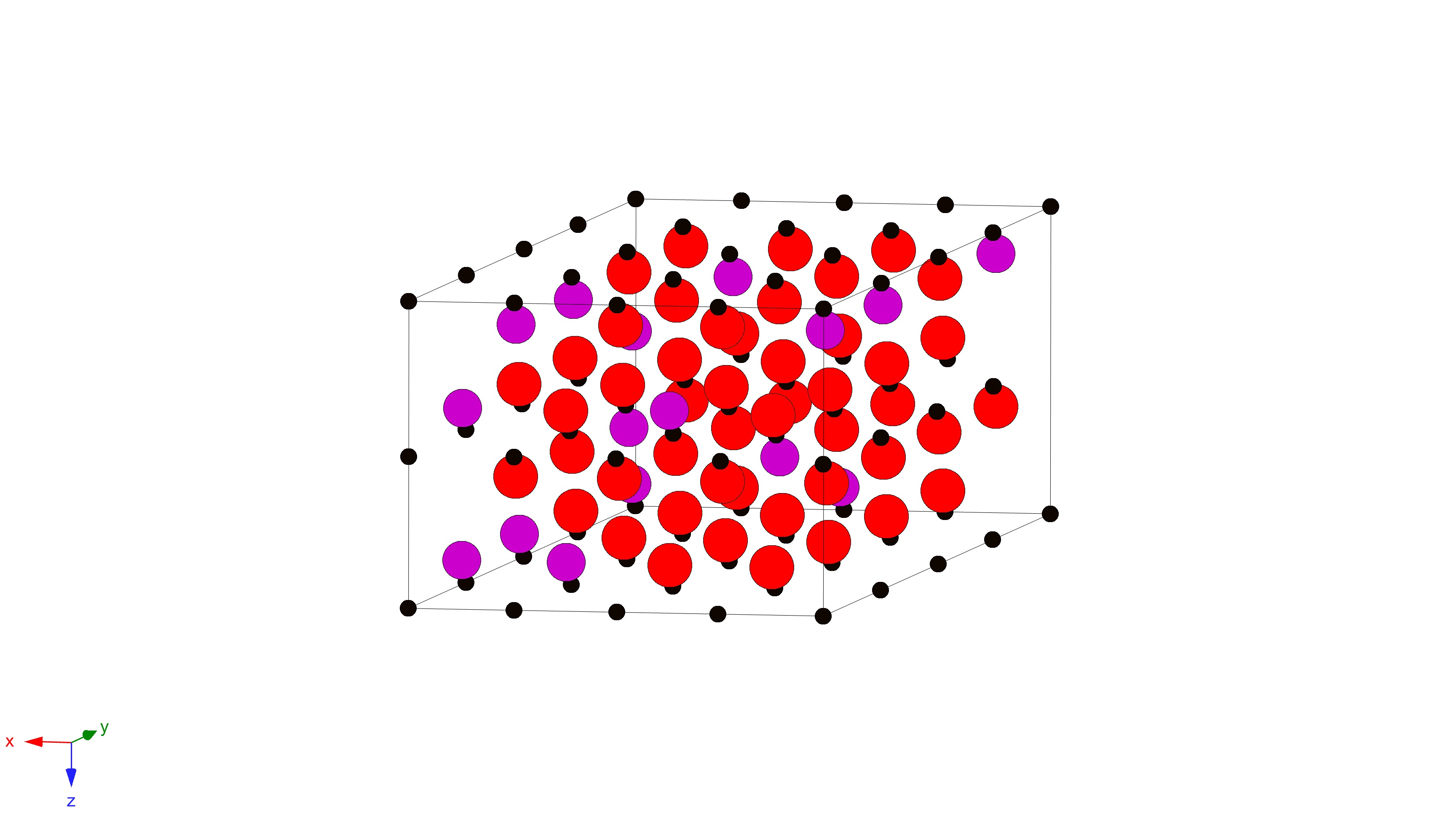

Supplement: Supplementary file 1 [file CP-018-C6CP00802J-s001.zip › mov_alloy_figures/mov2c/75mo/5g.jpg]

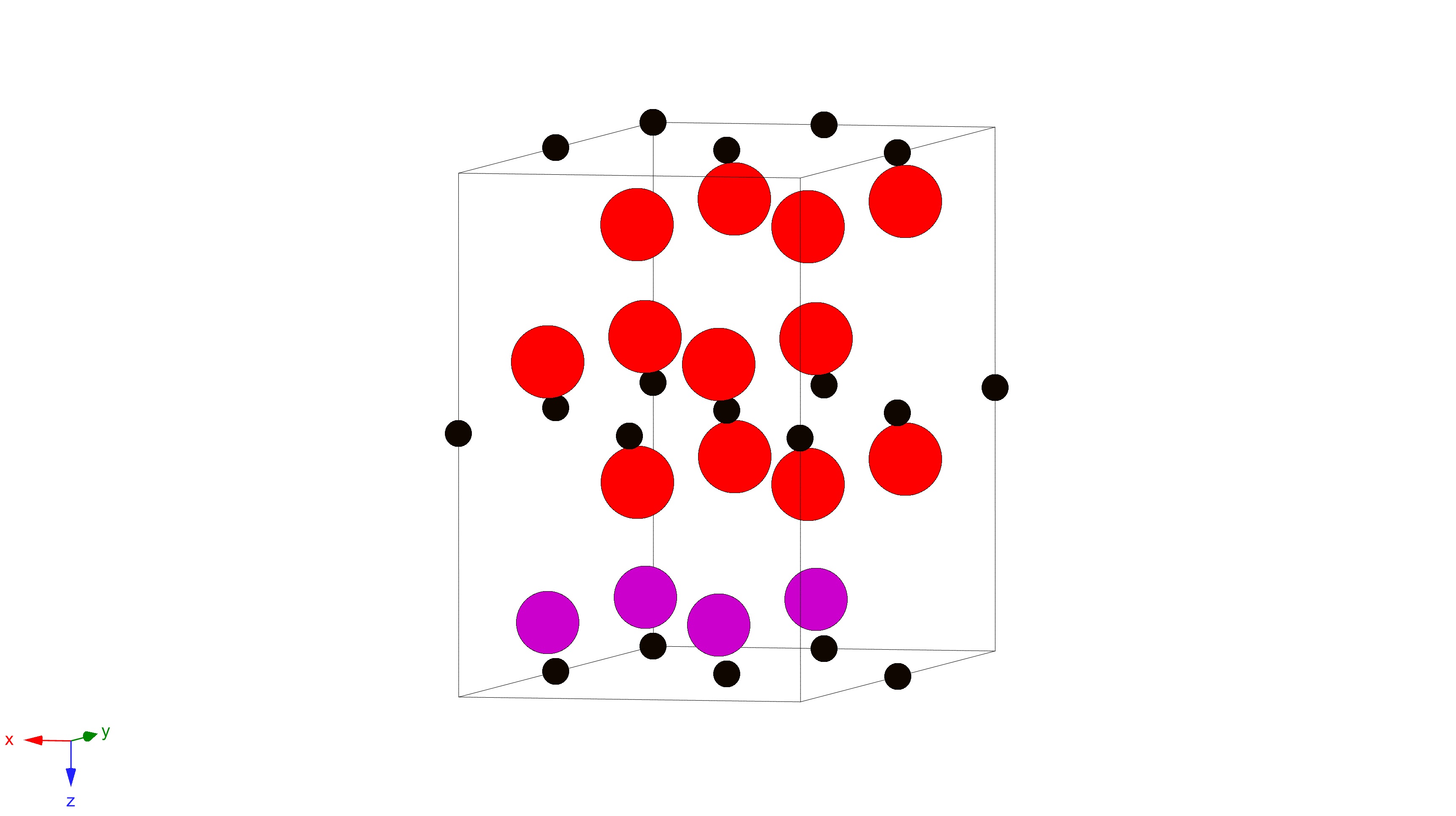

Supplement: Supplementary file 1 [file CP-018-C6CP00802J-s001.zip › mov_alloy_figures/mov2c/75mo/5h.jpg]

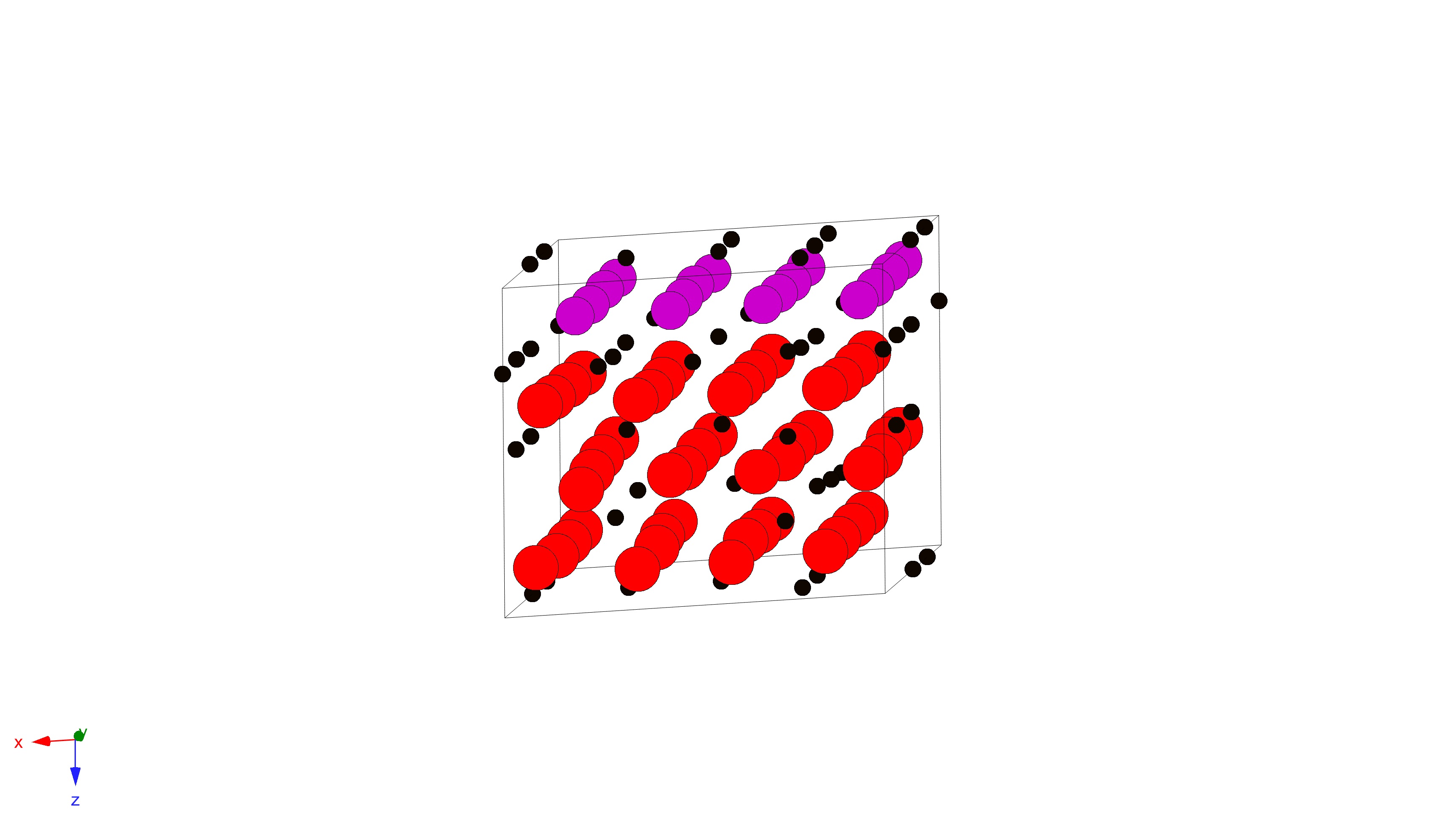

Supplement: Supplementary file 1 [file CP-018-C6CP00802J-s001.zip › mov_alloy_figures/mov2c/75mo/5i.jpg]

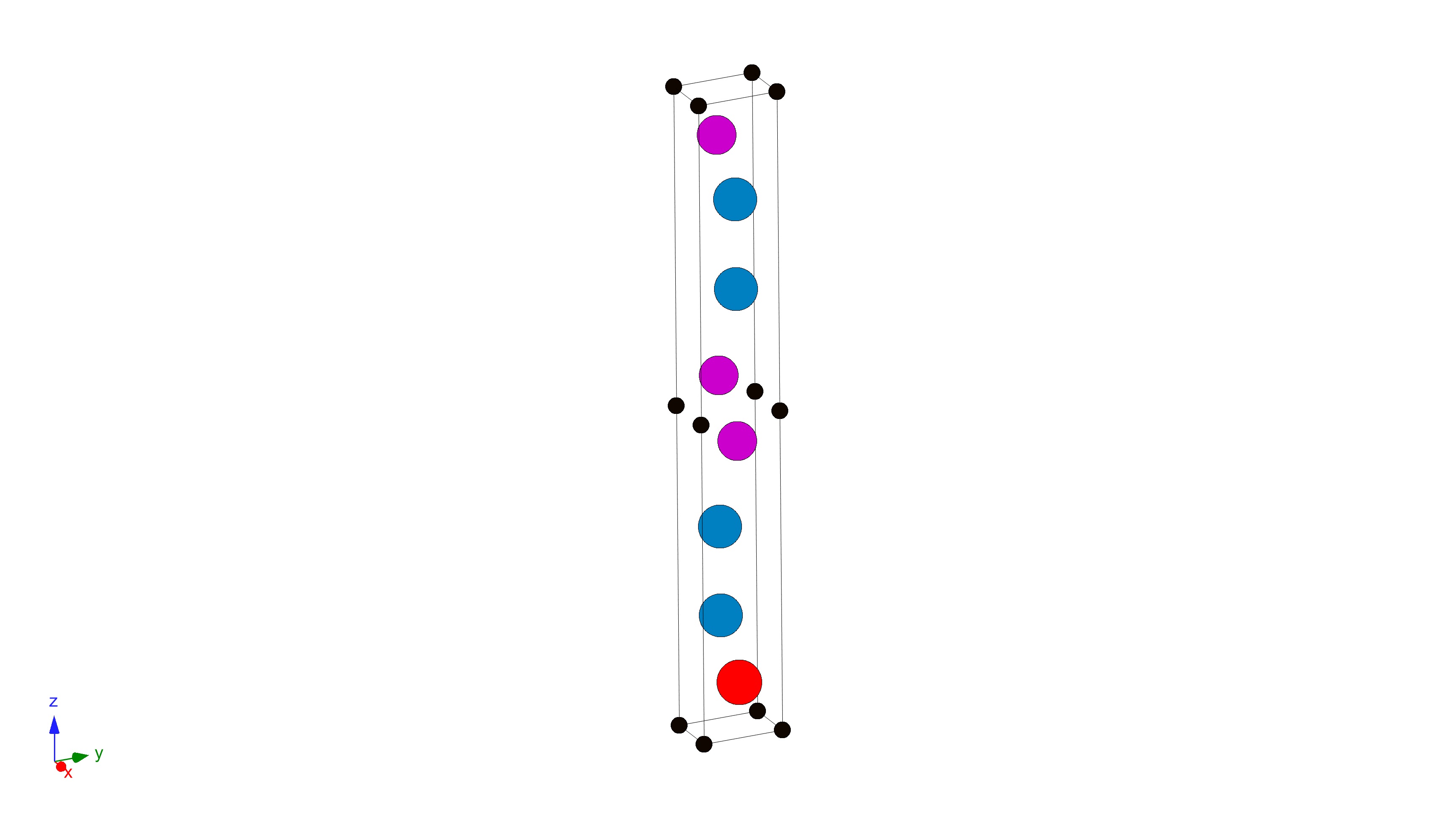

Supplement: Supplementary file 1 [file CP-018-C6CP00802J-s001.zip › mov_alloy_figures/mov2ga2c/25mo/6a.jpg]

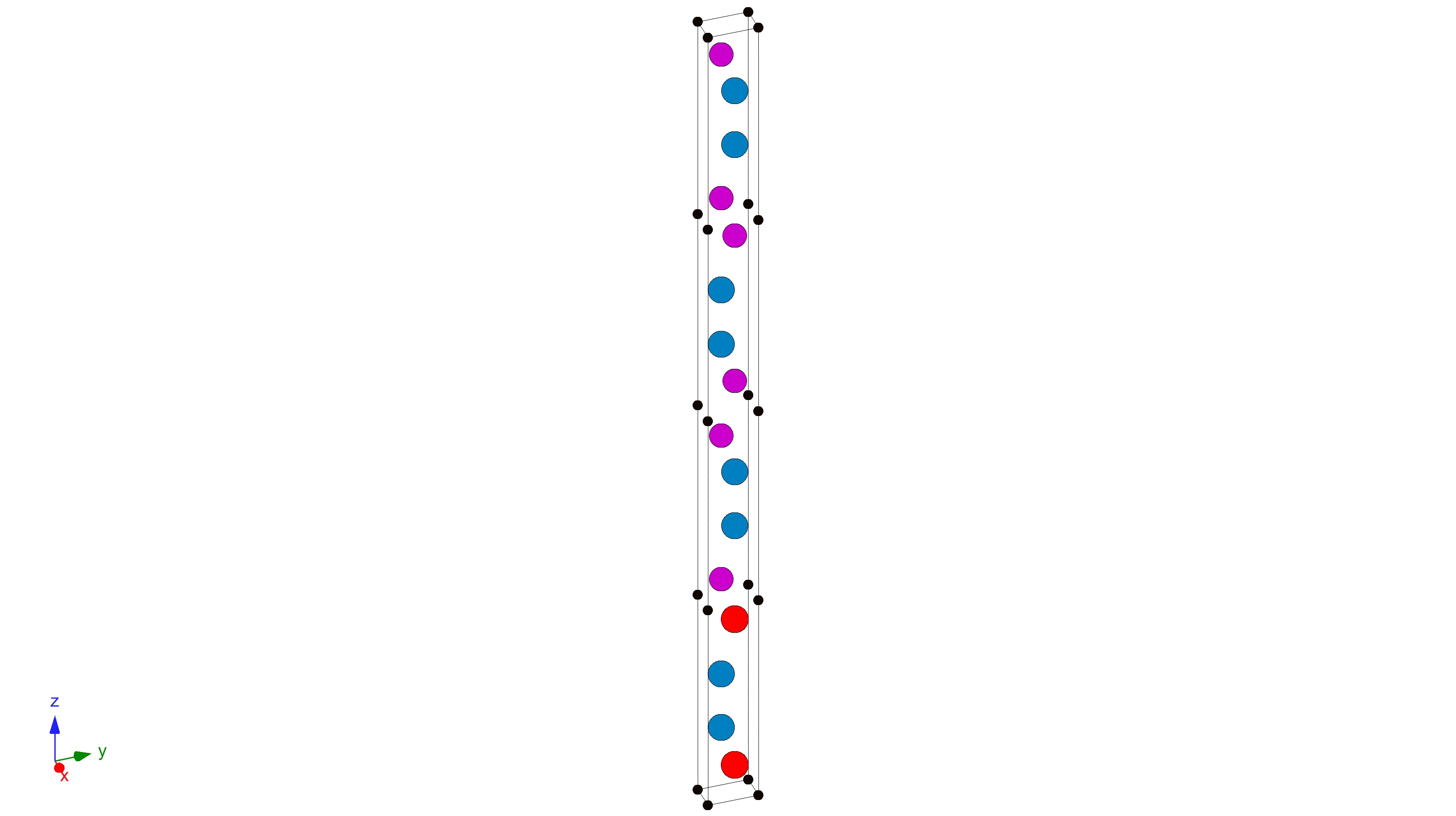

Supplement: Supplementary file 1 [file CP-018-C6CP00802J-s001.zip › mov_alloy_figures/mov2ga2c/25mo/6b.jpg]

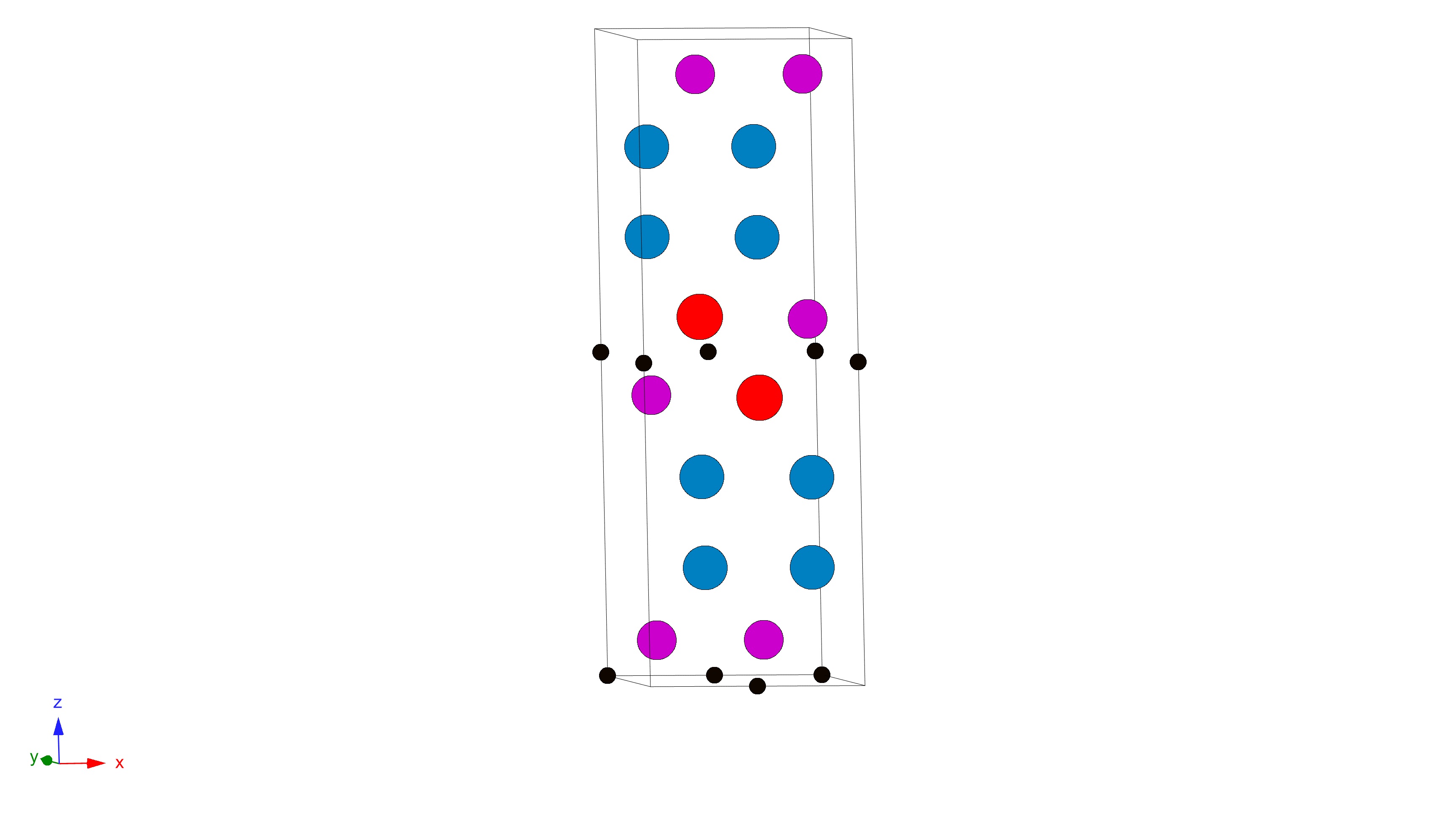

Supplement: Supplementary file 1 [file CP-018-C6CP00802J-s001.zip › mov_alloy_figures/mov2ga2c/25mo/6c.jpg]

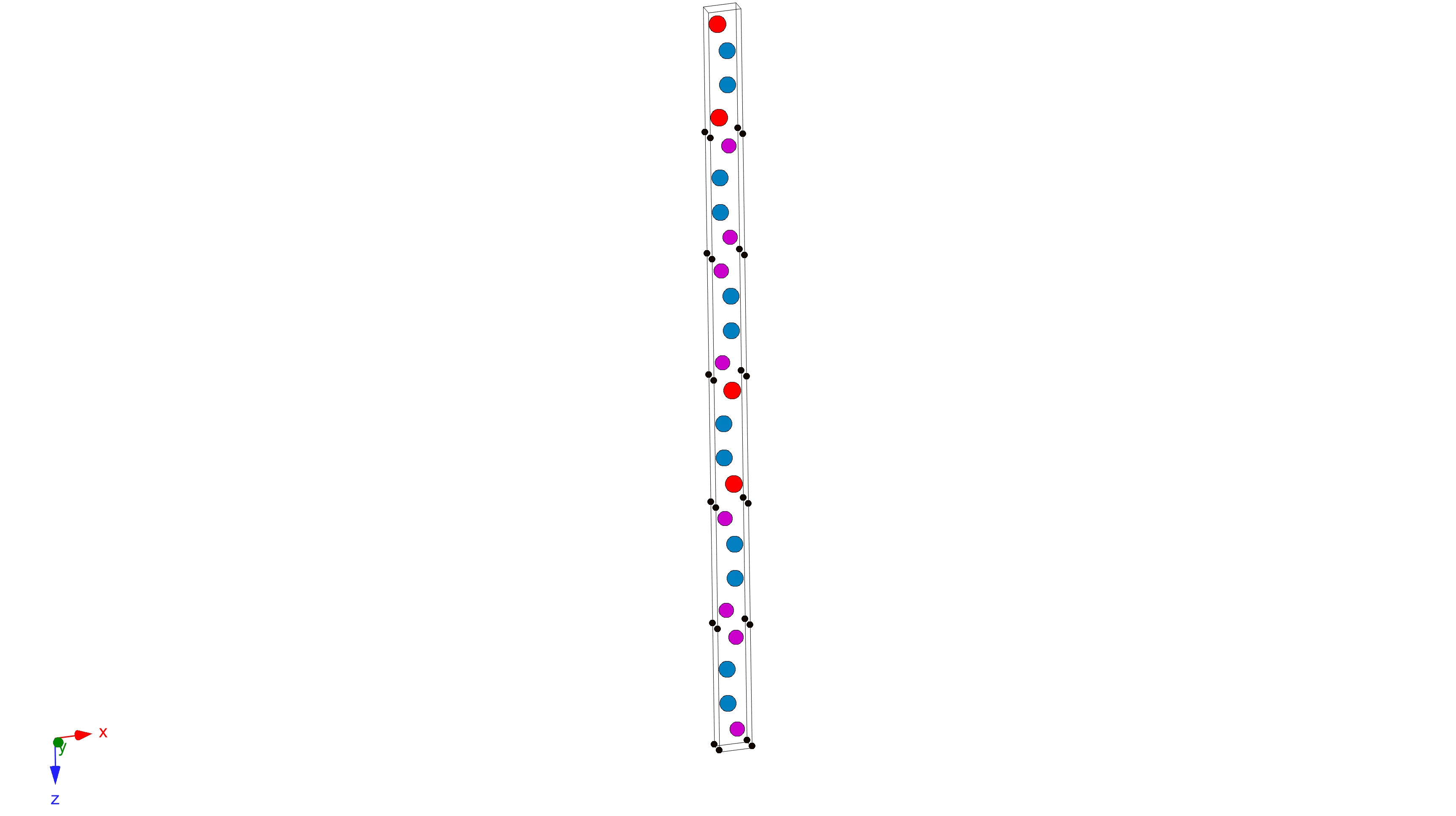

Supplement: Supplementary file 1 [file CP-018-C6CP00802J-s001.zip › mov_alloy_figures/mov2ga2c/333mo/6d.jpg]

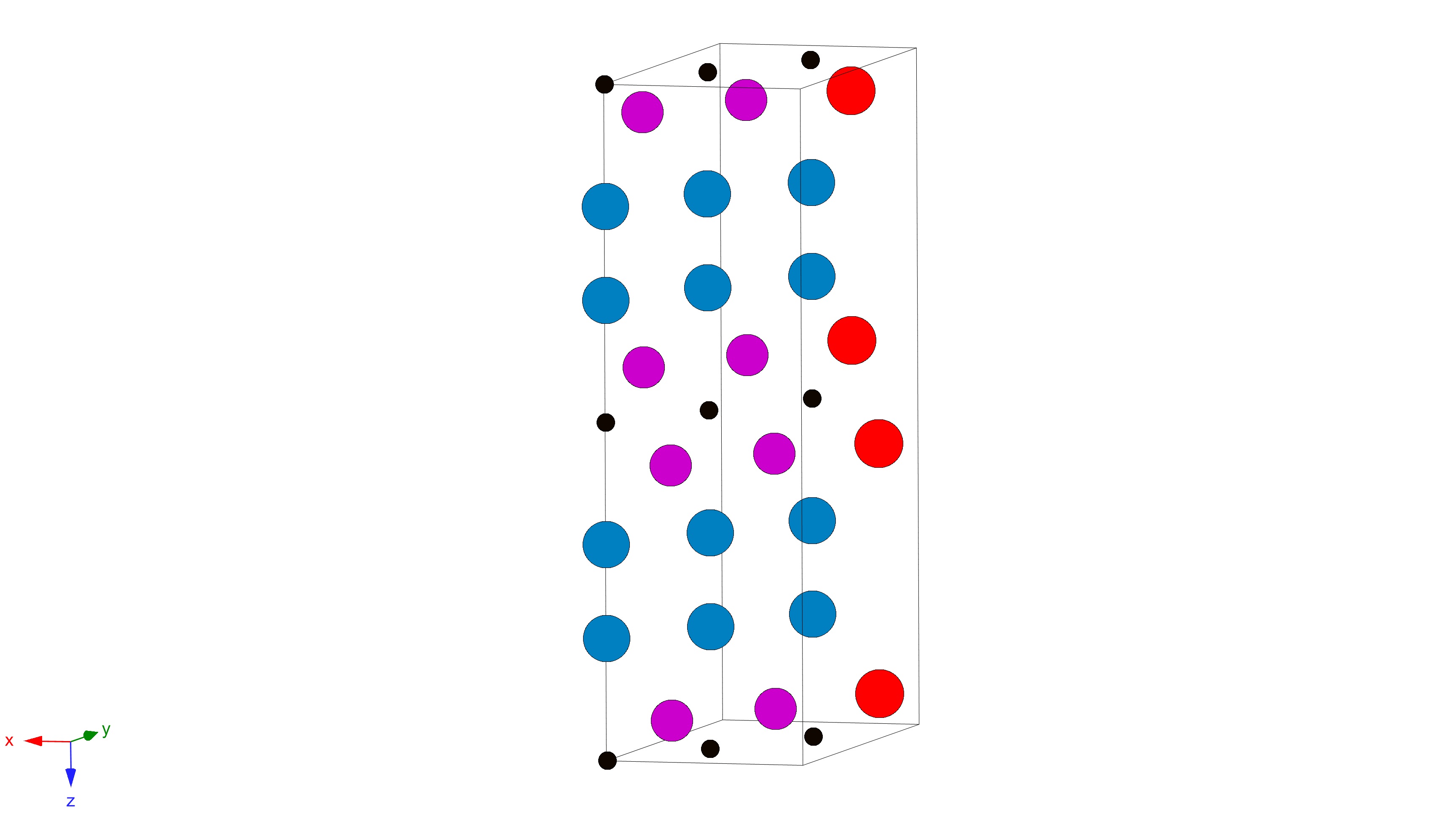

Supplement: Supplementary file 1 [file CP-018-C6CP00802J-s001.zip › mov_alloy_figures/mov2ga2c/333mo/6e.jpg]

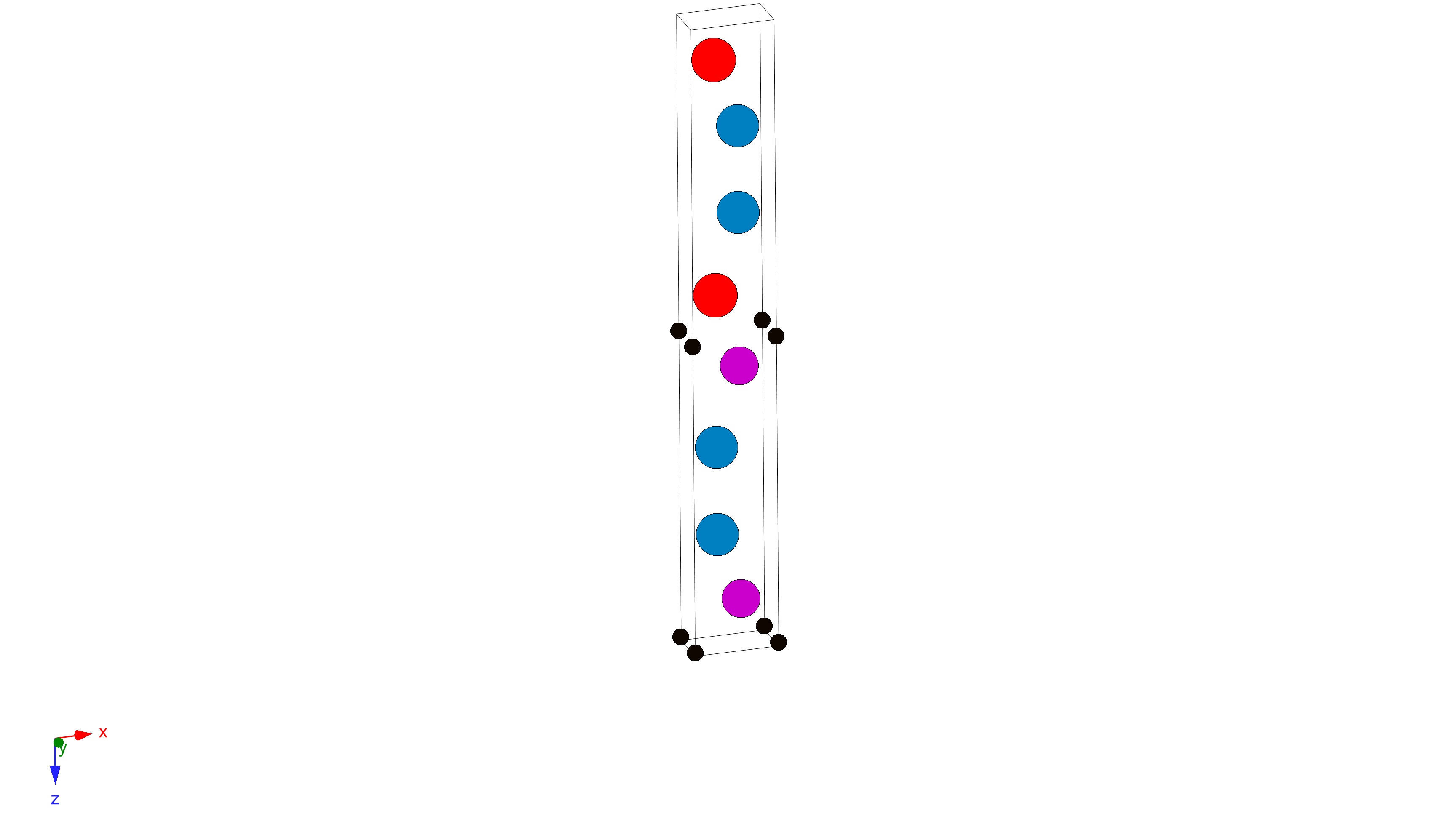

Supplement: Supplementary file 1 [file CP-018-C6CP00802J-s001.zip › mov_alloy_figures/mov2ga2c/50mo/6f.jpg]

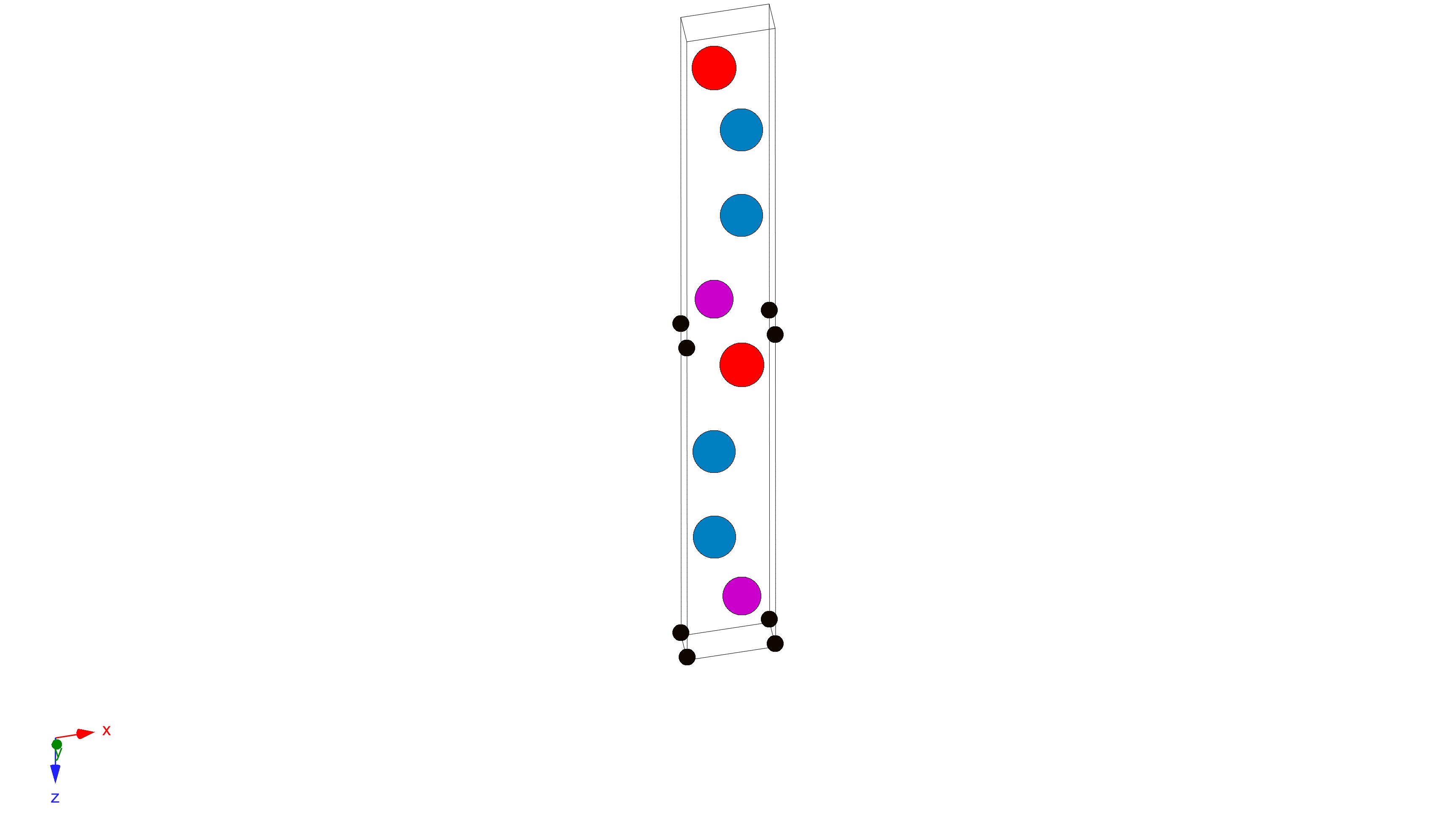

Supplement: Supplementary file 1 [file CP-018-C6CP00802J-s001.zip › mov_alloy_figures/mov2ga2c/50mo/6g.jpg]

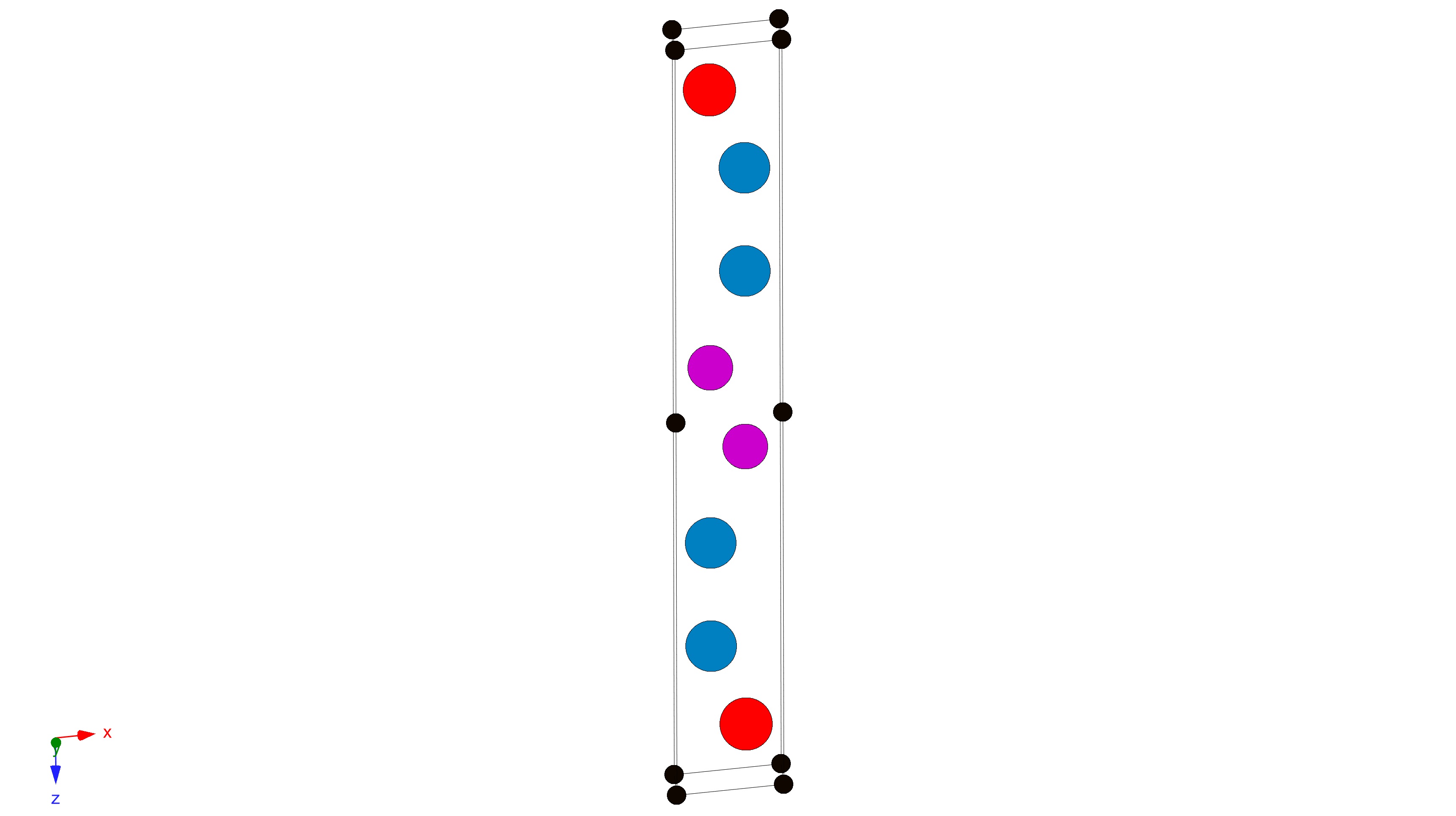

Supplement: Supplementary file 1 [file CP-018-C6CP00802J-s001.zip › mov_alloy_figures/mov2ga2c/50mo/6h.jpg]

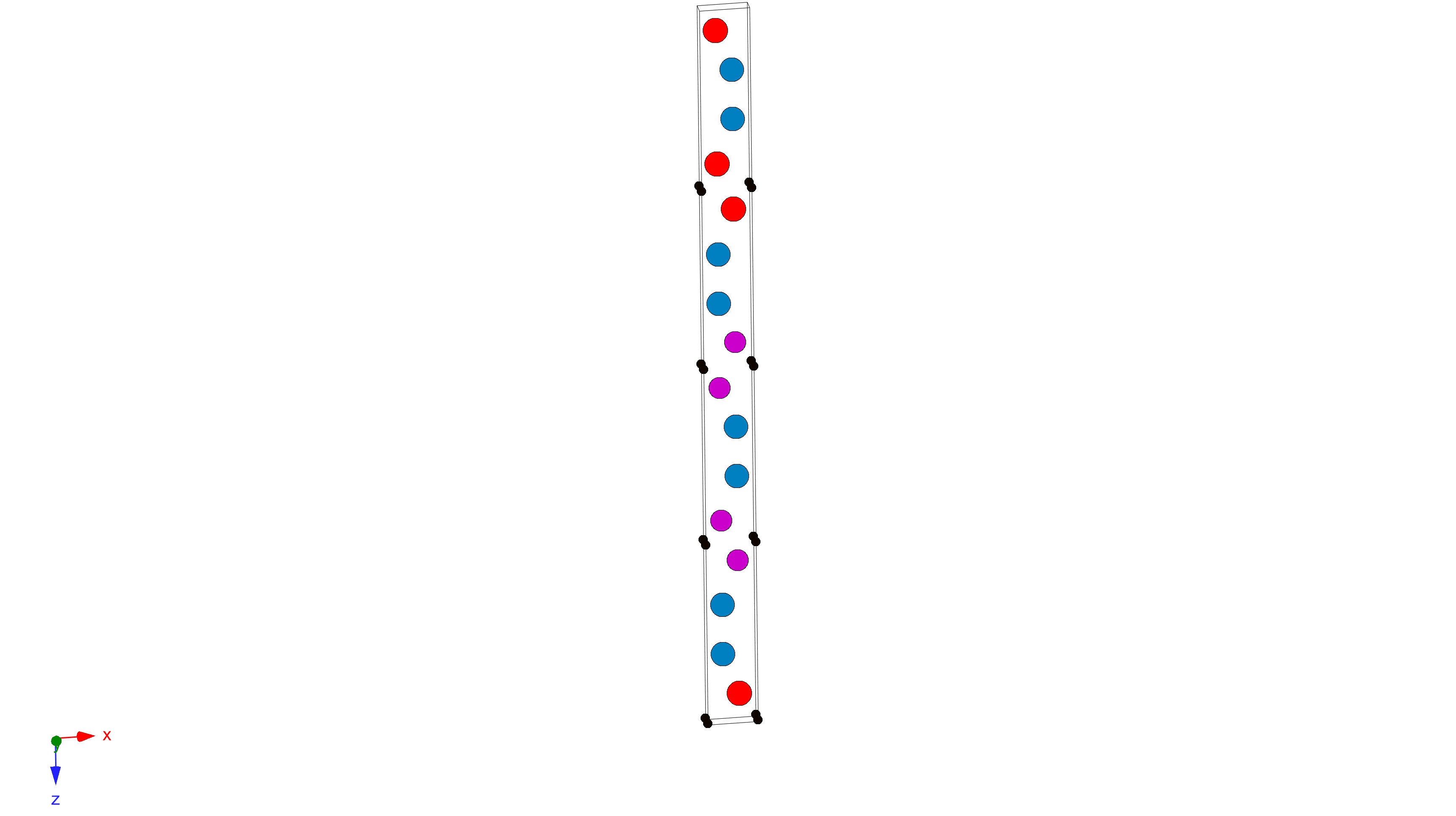

Supplement: Supplementary file 1 [file CP-018-C6CP00802J-s001.zip › mov_alloy_figures/mov2ga2c/50mo/6i.jpg]

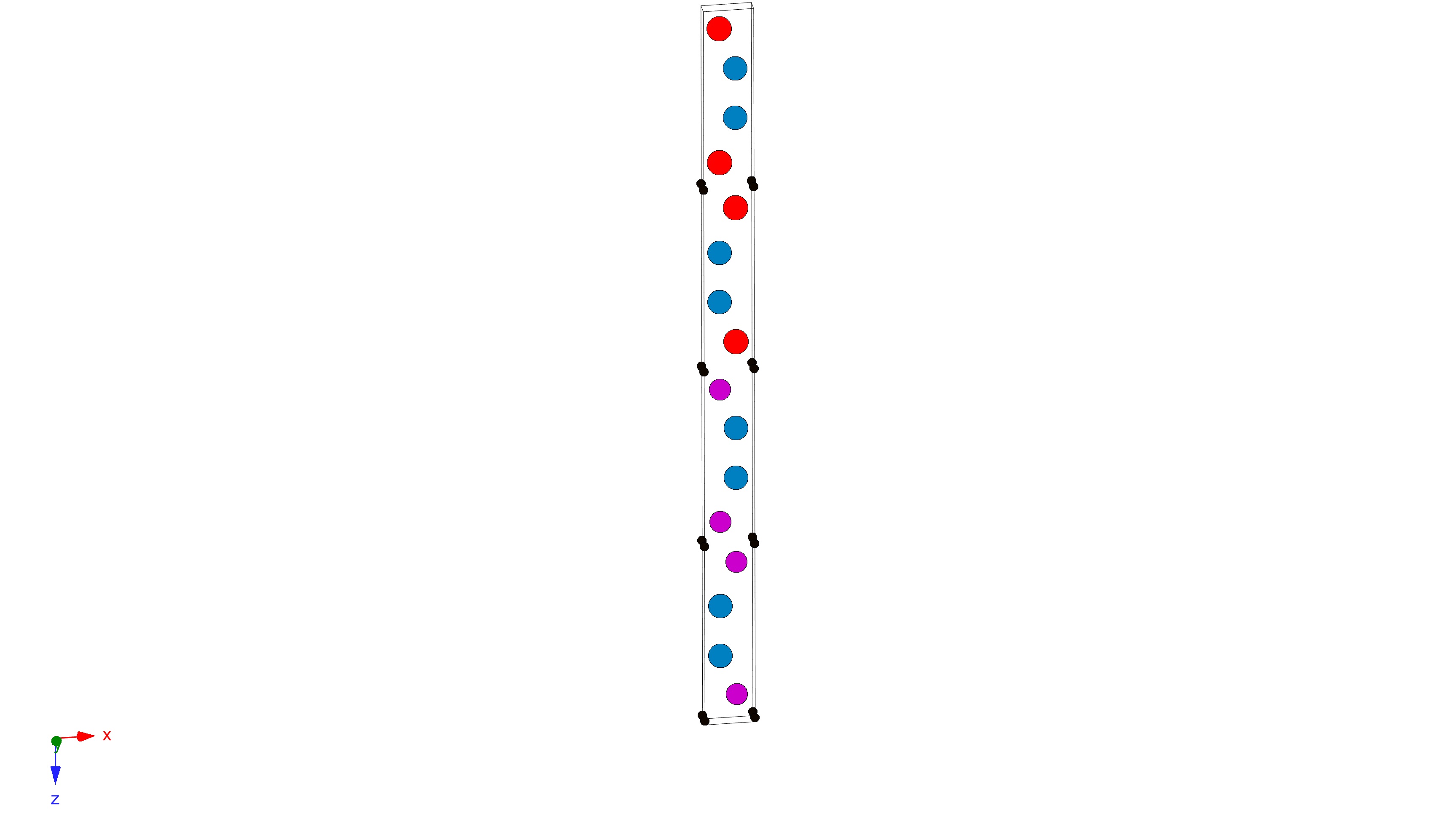

Supplement: Supplementary file 1 [file CP-018-C6CP00802J-s001.zip › mov_alloy_figures/mov2ga2c/50mo/6j.jpg]

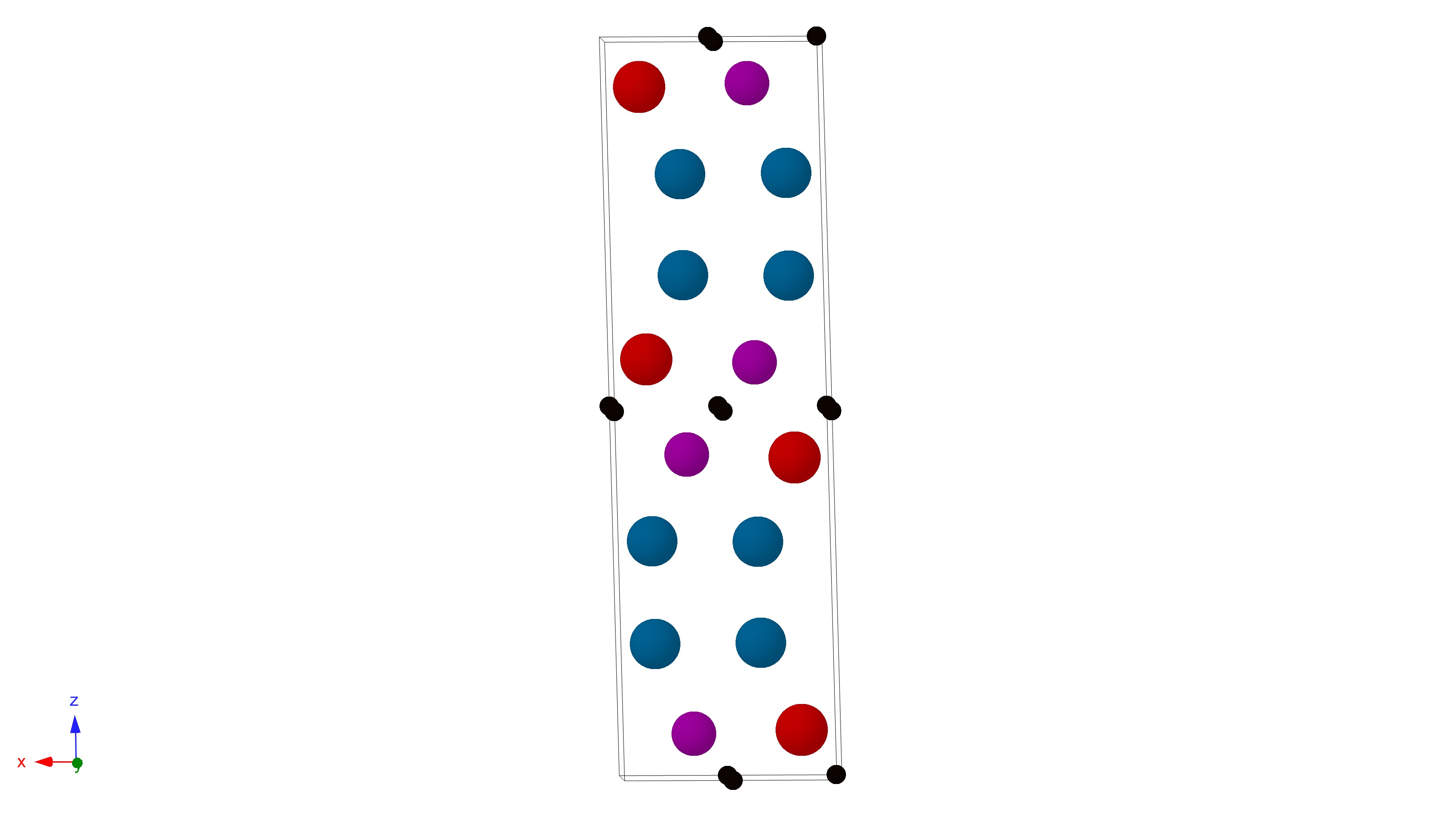

Supplement: Supplementary file 1 [file CP-018-C6CP00802J-s001.zip › mov_alloy_figures/mov2ga2c/50mo/6k.jpg]

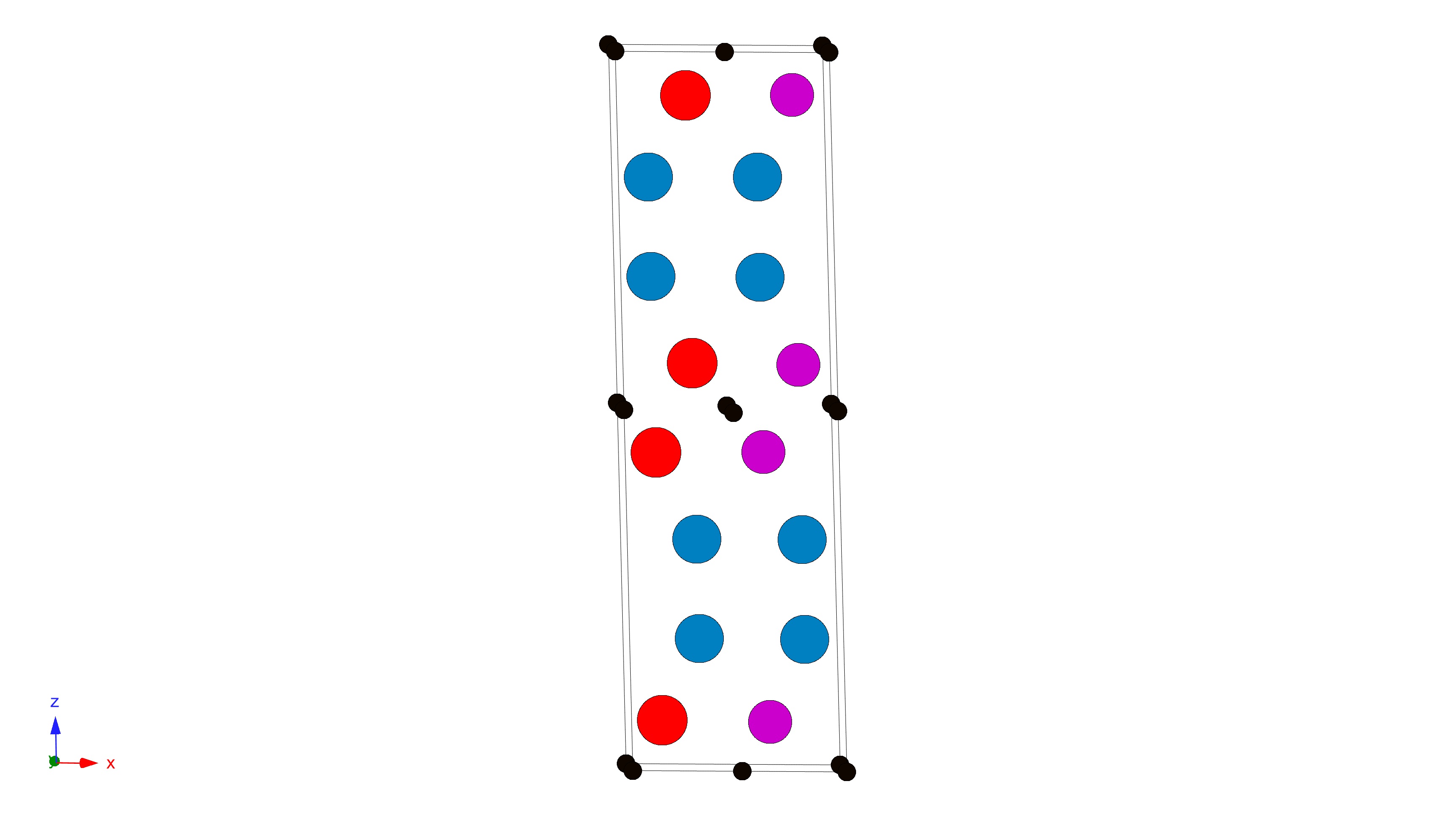

Supplement: Supplementary file 1 [file CP-018-C6CP00802J-s001.zip › mov_alloy_figures/mov2ga2c/50mo/6l.jpg]

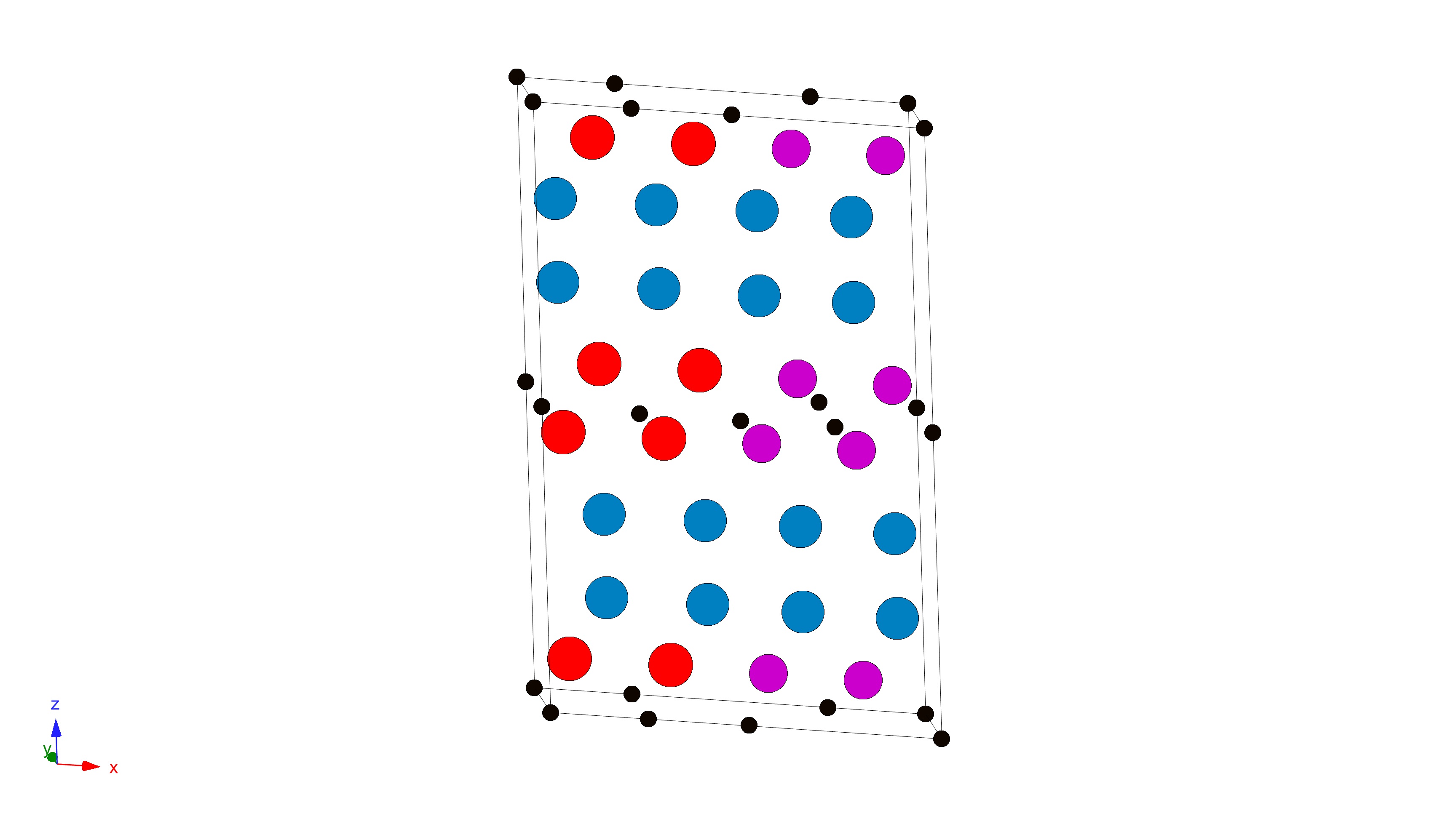

Supplement: Supplementary file 1 [file CP-018-C6CP00802J-s001.zip › mov_alloy_figures/mov2ga2c/50mo/6m.jpg]

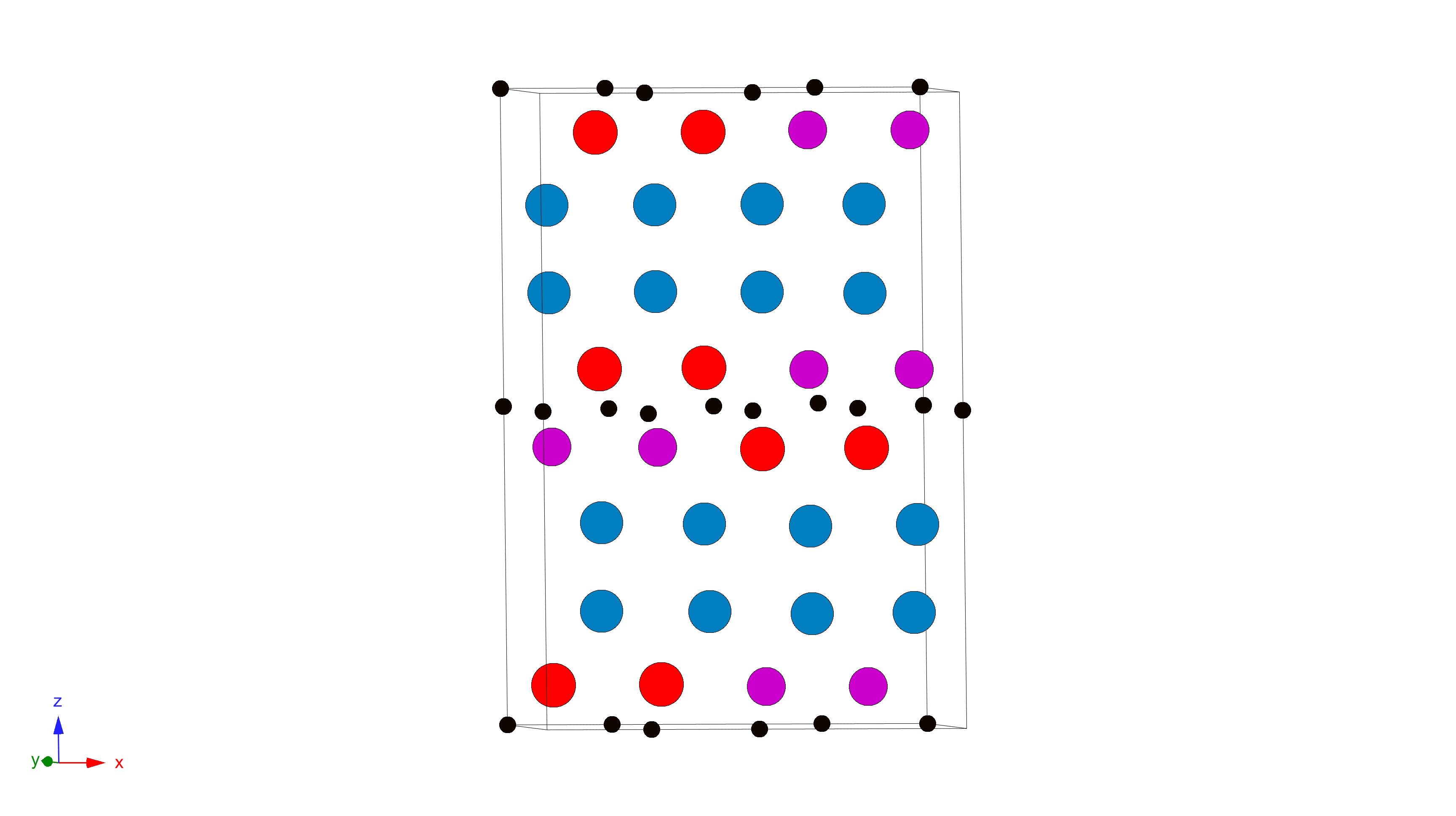

Supplement: Supplementary file 1 [file CP-018-C6CP00802J-s001.zip › mov_alloy_figures/mov2ga2c/50mo/6n.jpg]

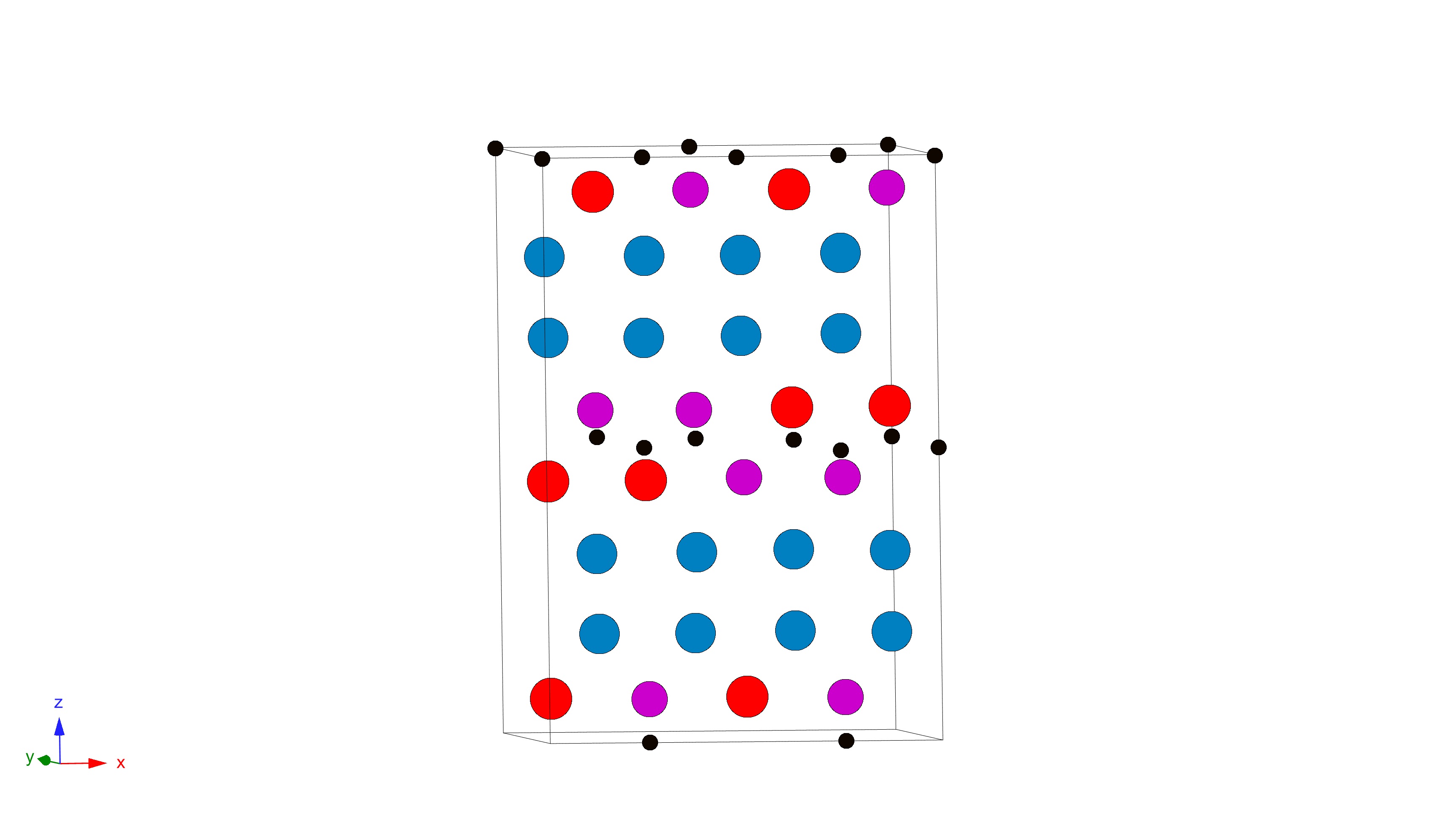

Supplement: Supplementary file 1 [file CP-018-C6CP00802J-s001.zip › mov_alloy_figures/mov2ga2c/50mo/6o.jpg]

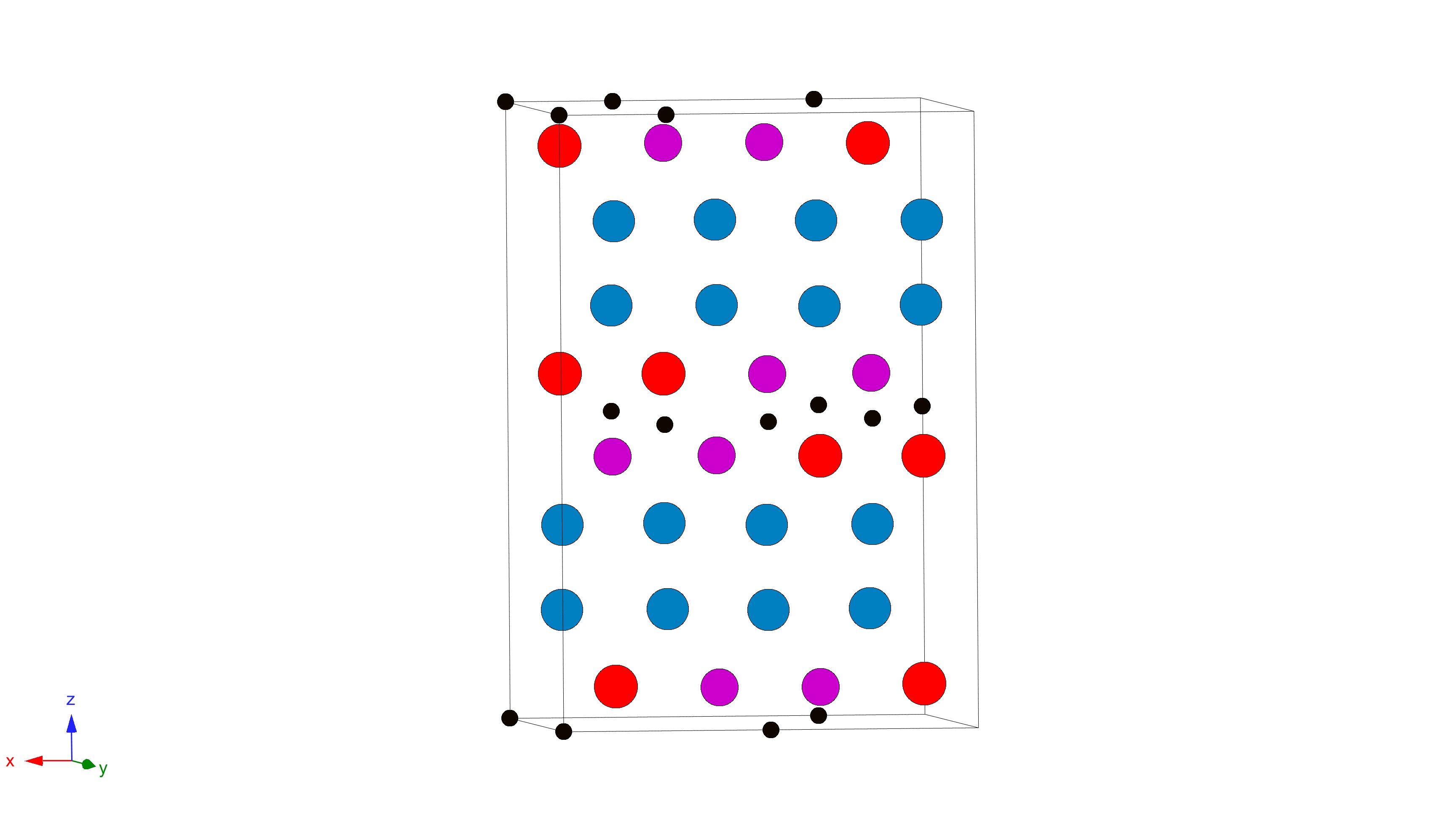

Supplement: Supplementary file 1 [file CP-018-C6CP00802J-s001.zip › mov_alloy_figures/mov2ga2c/50mo/6p.jpg]

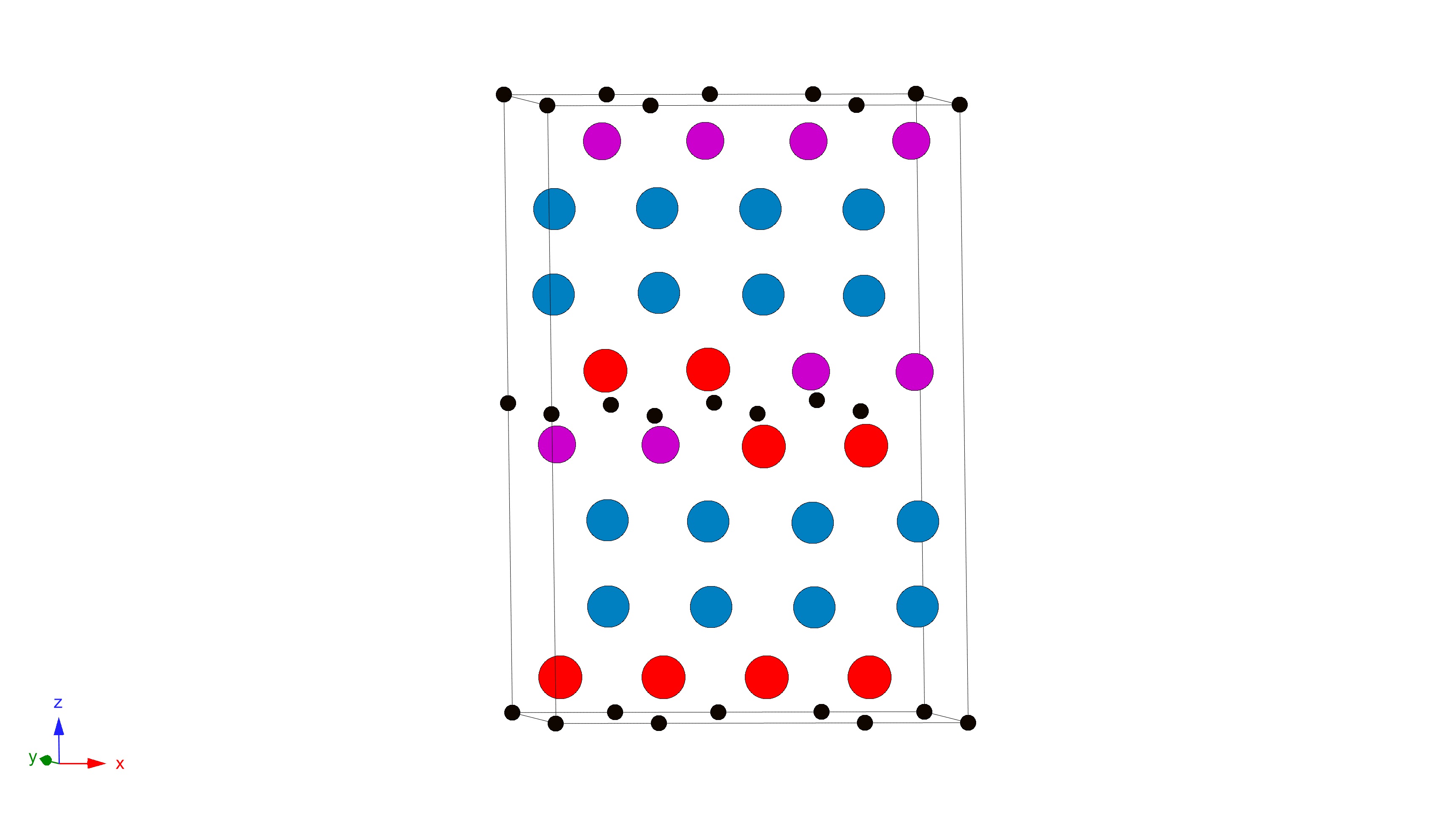

Supplement: Supplementary file 1 [file CP-018-C6CP00802J-s001.zip › mov_alloy_figures/mov2ga2c/50mo/6q.jpg]

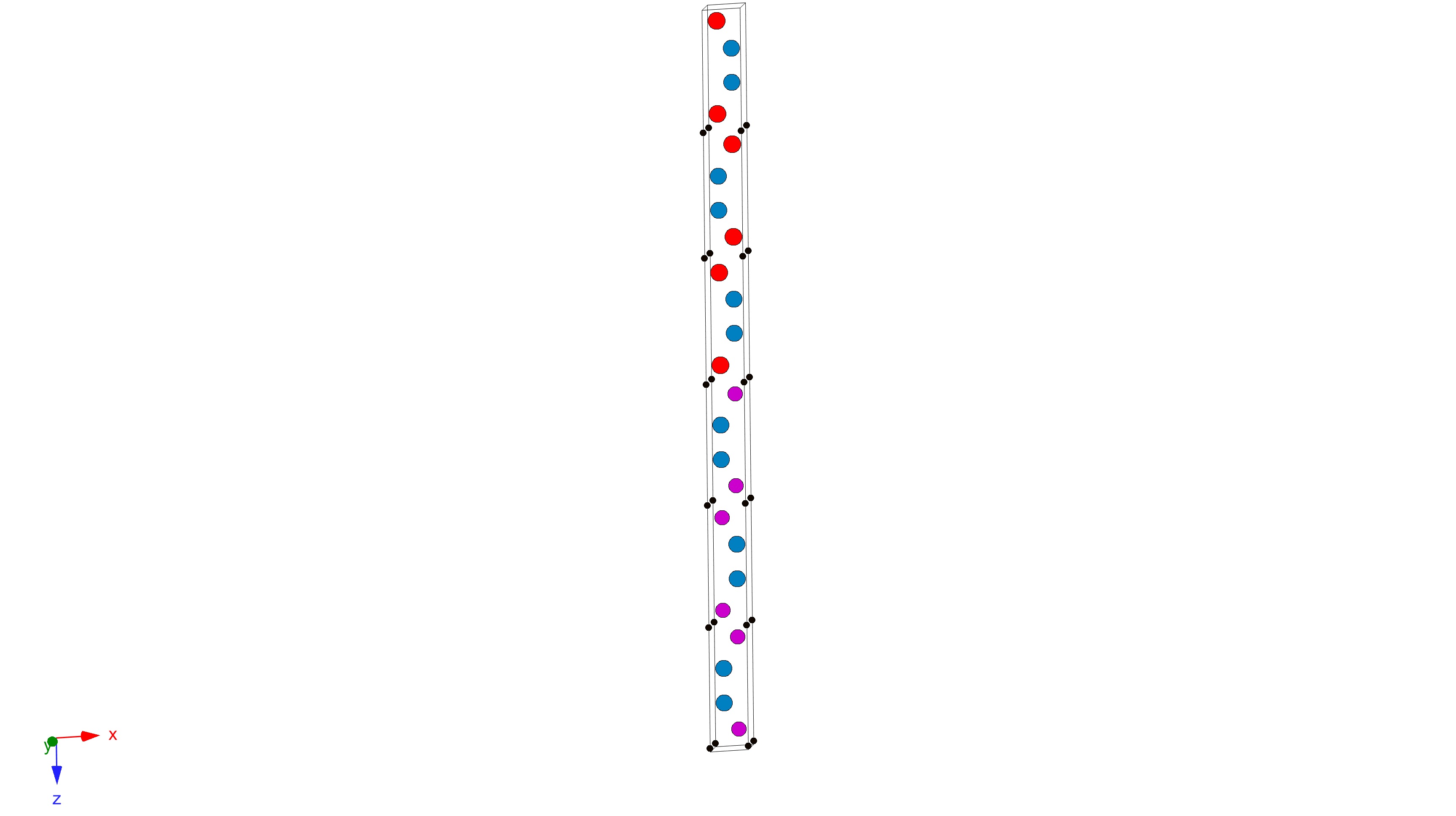

Supplement: Supplementary file 1 [file CP-018-C6CP00802J-s001.zip › mov_alloy_figures/mov2ga2c/50mo/6r.jpg]

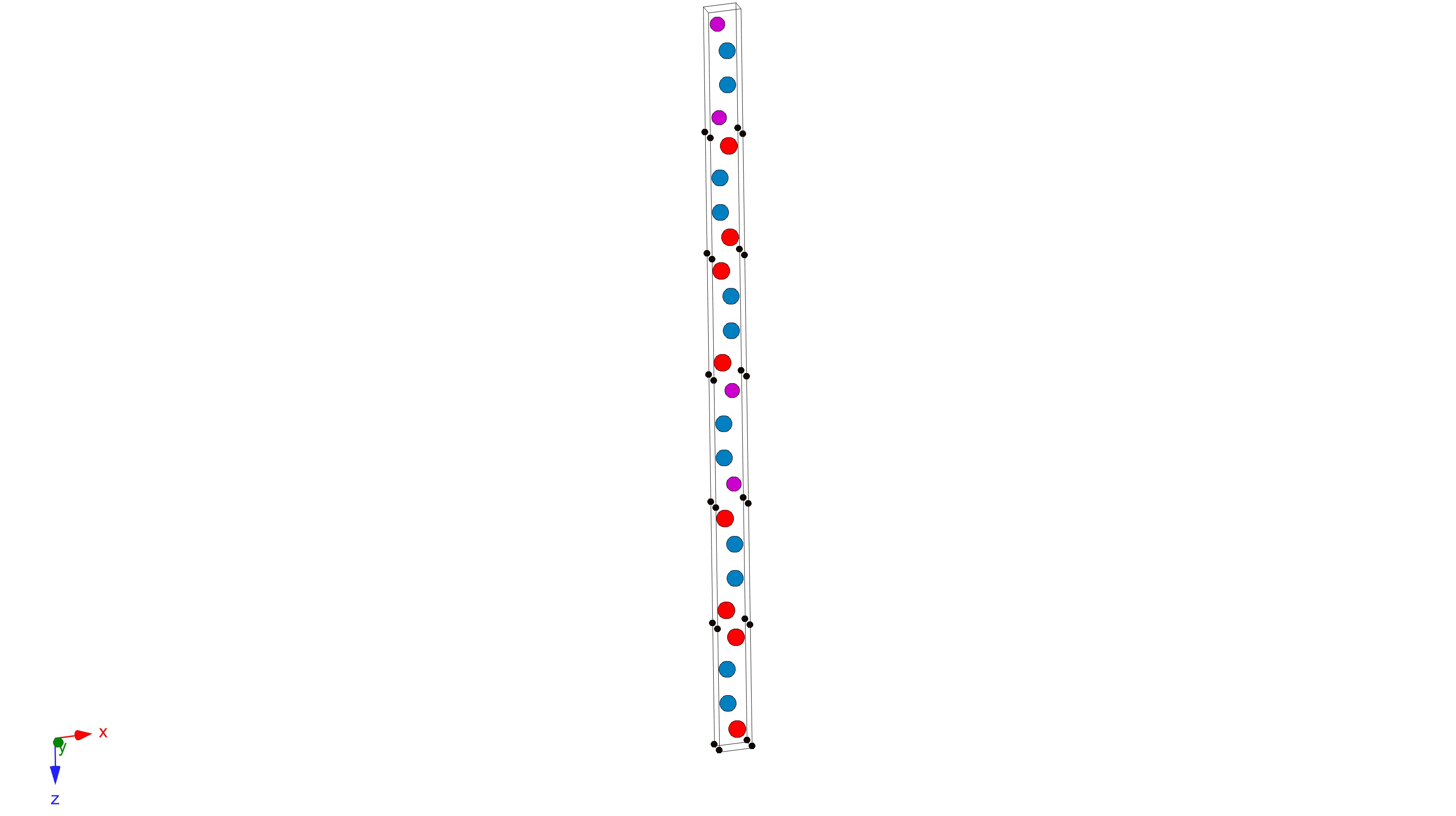

Supplement: Supplementary file 1 [file CP-018-C6CP00802J-s001.zip › mov_alloy_figures/mov2ga2c/667mo/6s.jpg]

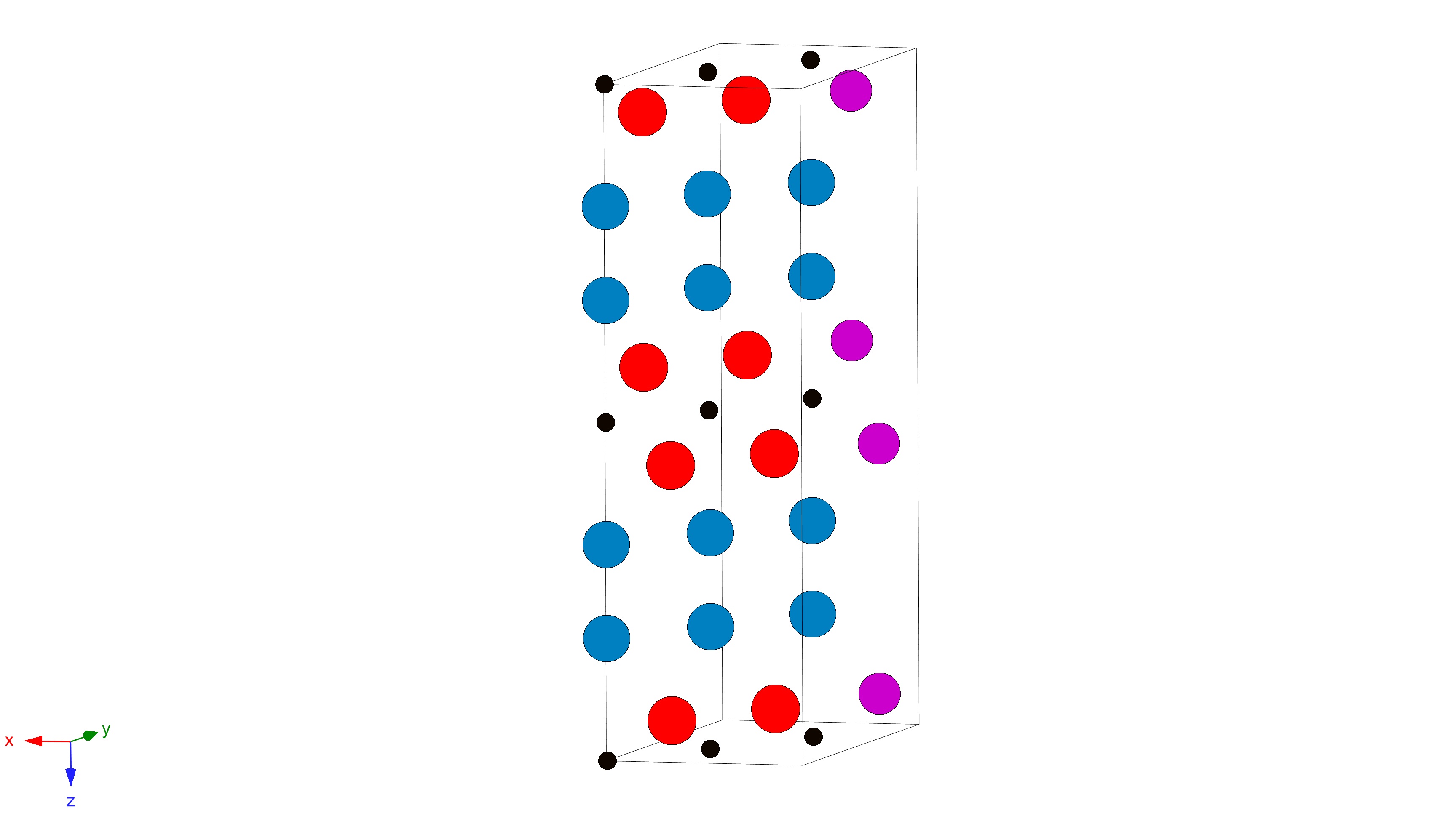

Supplement: Supplementary file 1 [file CP-018-C6CP00802J-s001.zip › mov_alloy_figures/mov2ga2c/667mo/6t.jpg]

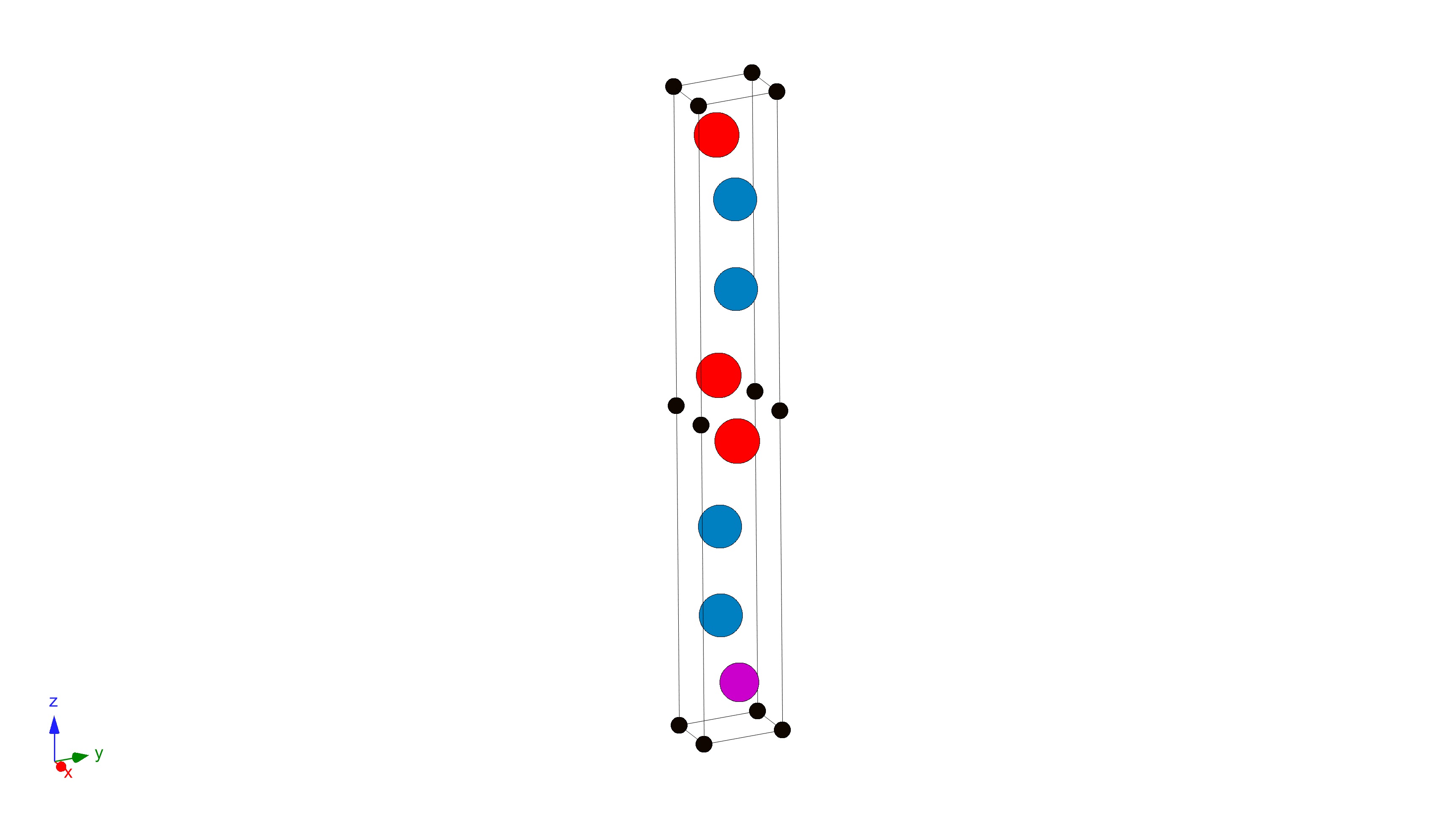

Supplement: Supplementary file 1 [file CP-018-C6CP00802J-s001.zip › mov_alloy_figures/mov2ga2c/75mo/6u.jpg]

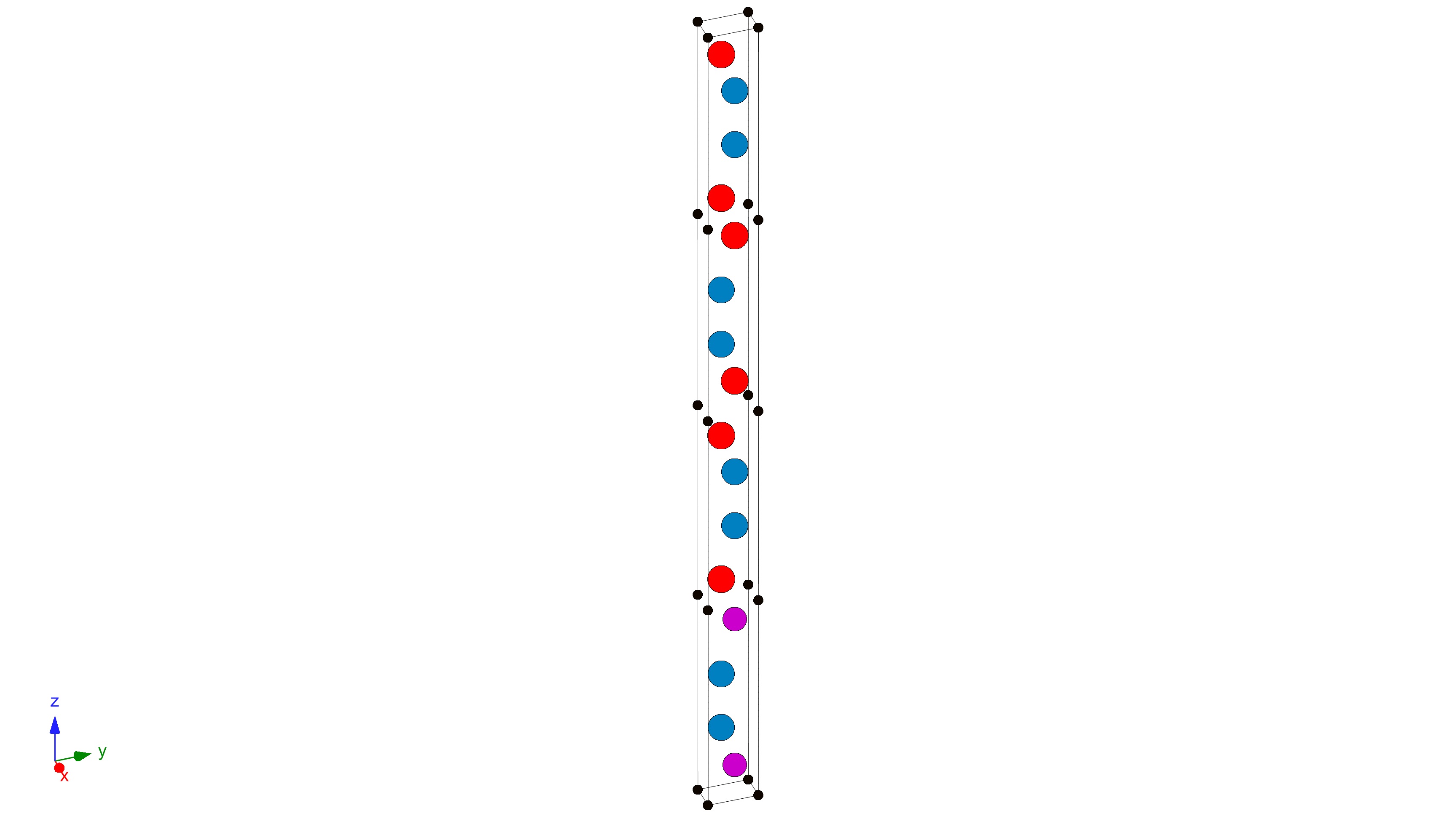

Supplement: Supplementary file 1 [file CP-018-C6CP00802J-s001.zip › mov_alloy_figures/mov2ga2c/75mo/6v.jpg]

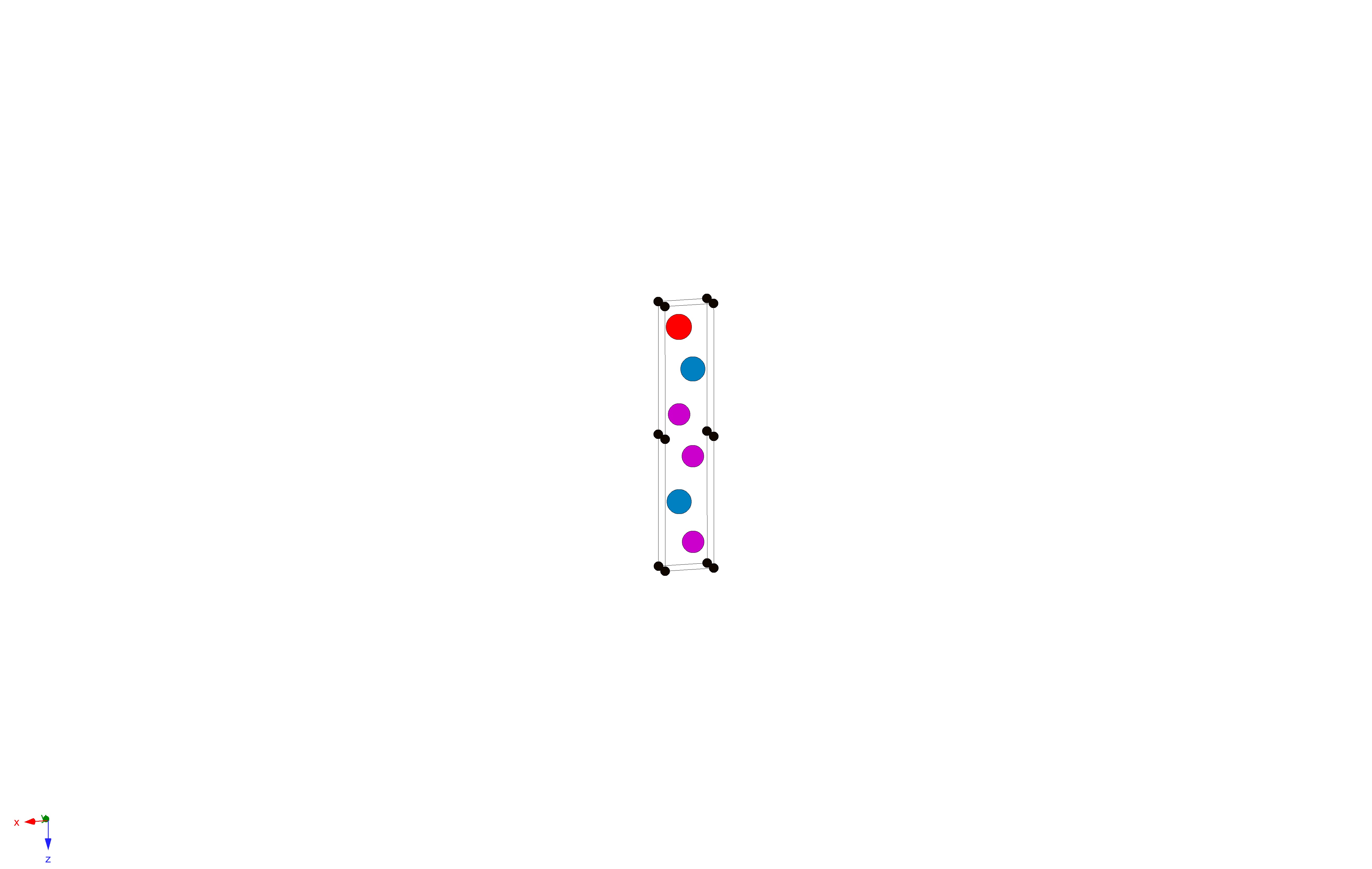

Supplement: Supplementary file 1 [file CP-018-C6CP00802J-s001.zip › mov_alloy_figures/mov2gac/25mo/2a.jpg]

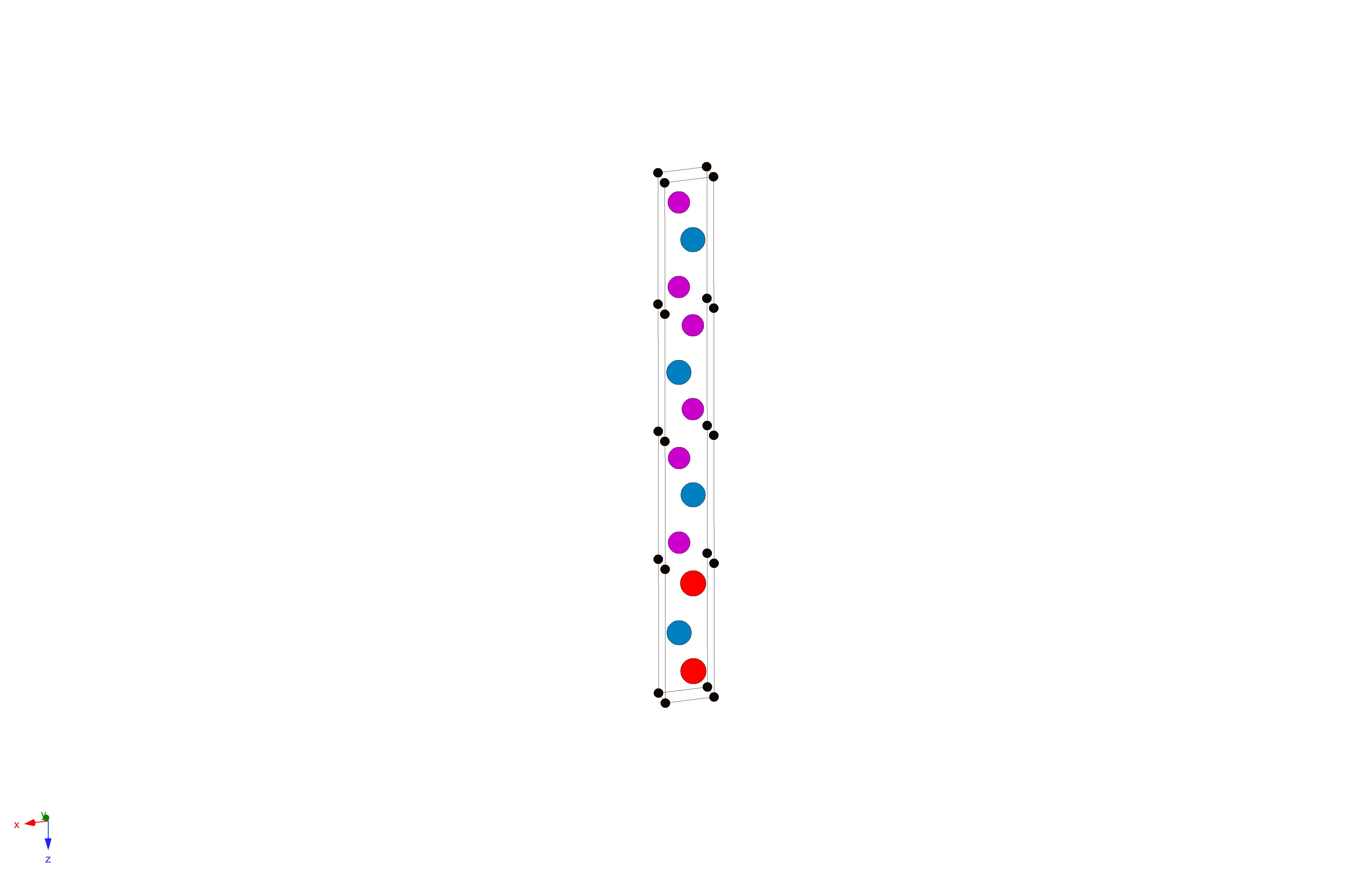

Supplement: Supplementary file 1 [file CP-018-C6CP00802J-s001.zip › mov_alloy_figures/mov2gac/25mo/2b.jpg]

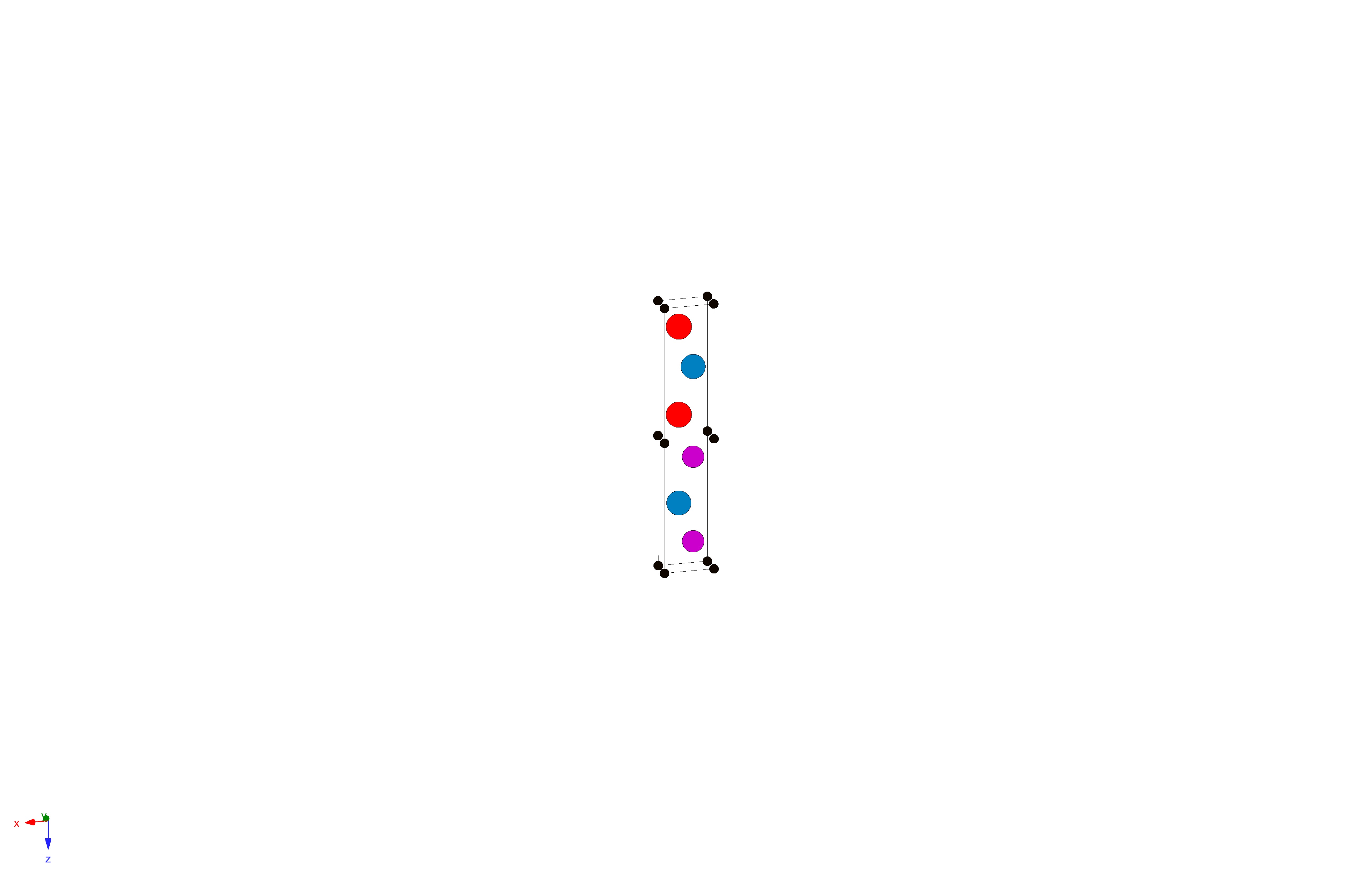

Supplement: Supplementary file 1 [file CP-018-C6CP00802J-s001.zip › mov_alloy_figures/mov2gac/50mo/2c.jpg]

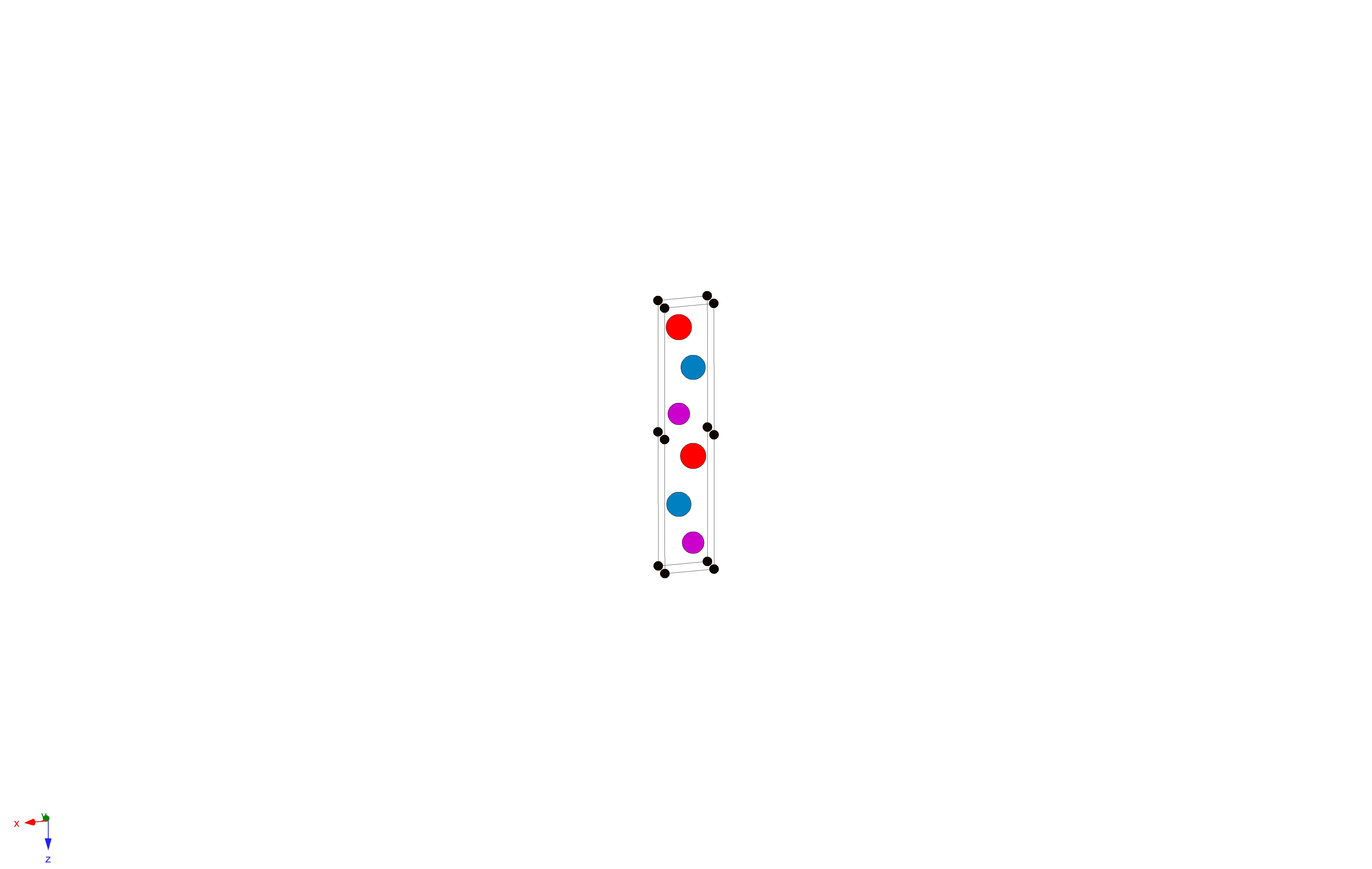

Supplement: Supplementary file 1 [file CP-018-C6CP00802J-s001.zip › mov_alloy_figures/mov2gac/50mo/2d.jpg]

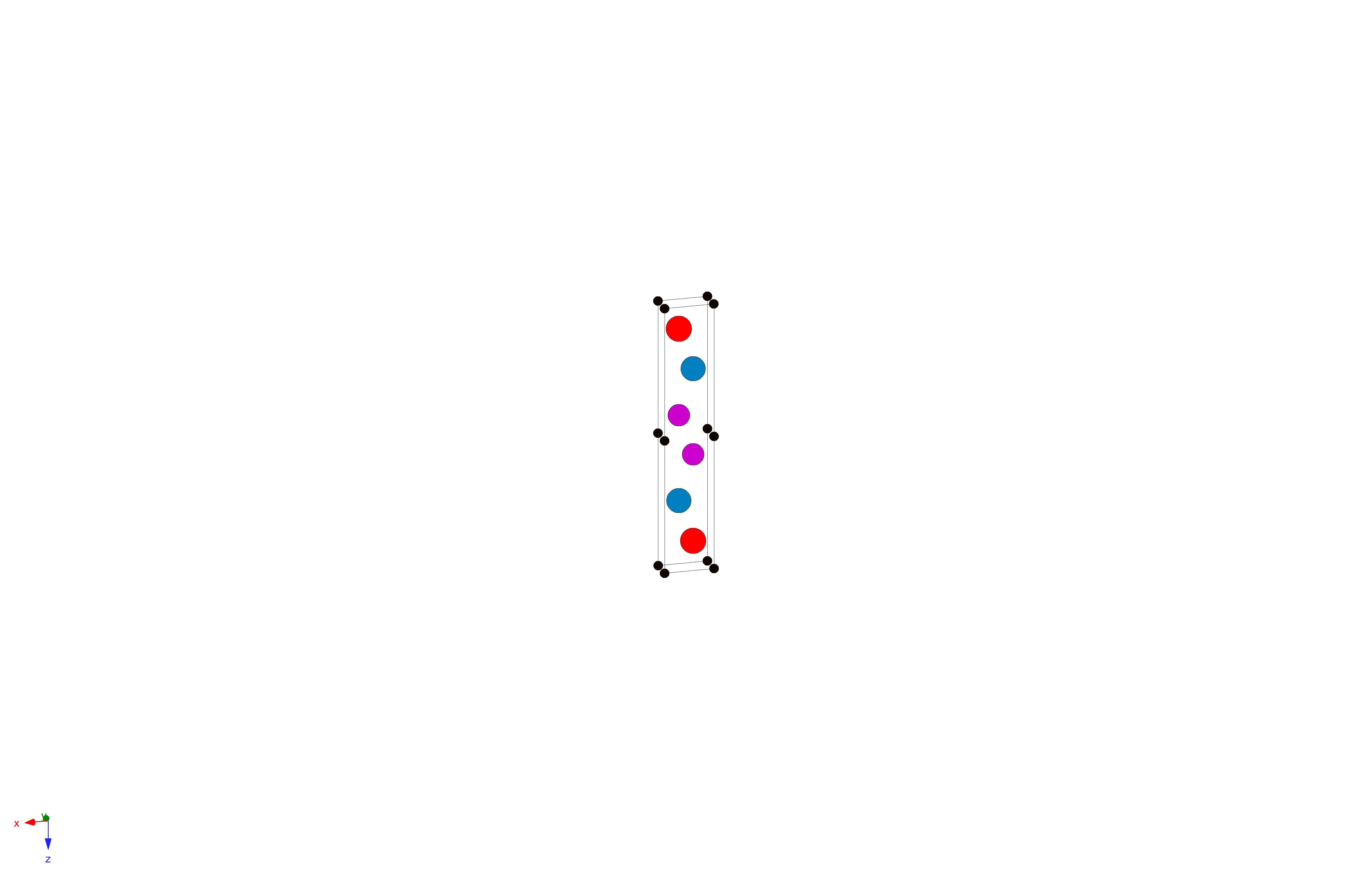

Supplement: Supplementary file 1 [file CP-018-C6CP00802J-s001.zip › mov_alloy_figures/mov2gac/50mo/2e.jpg]

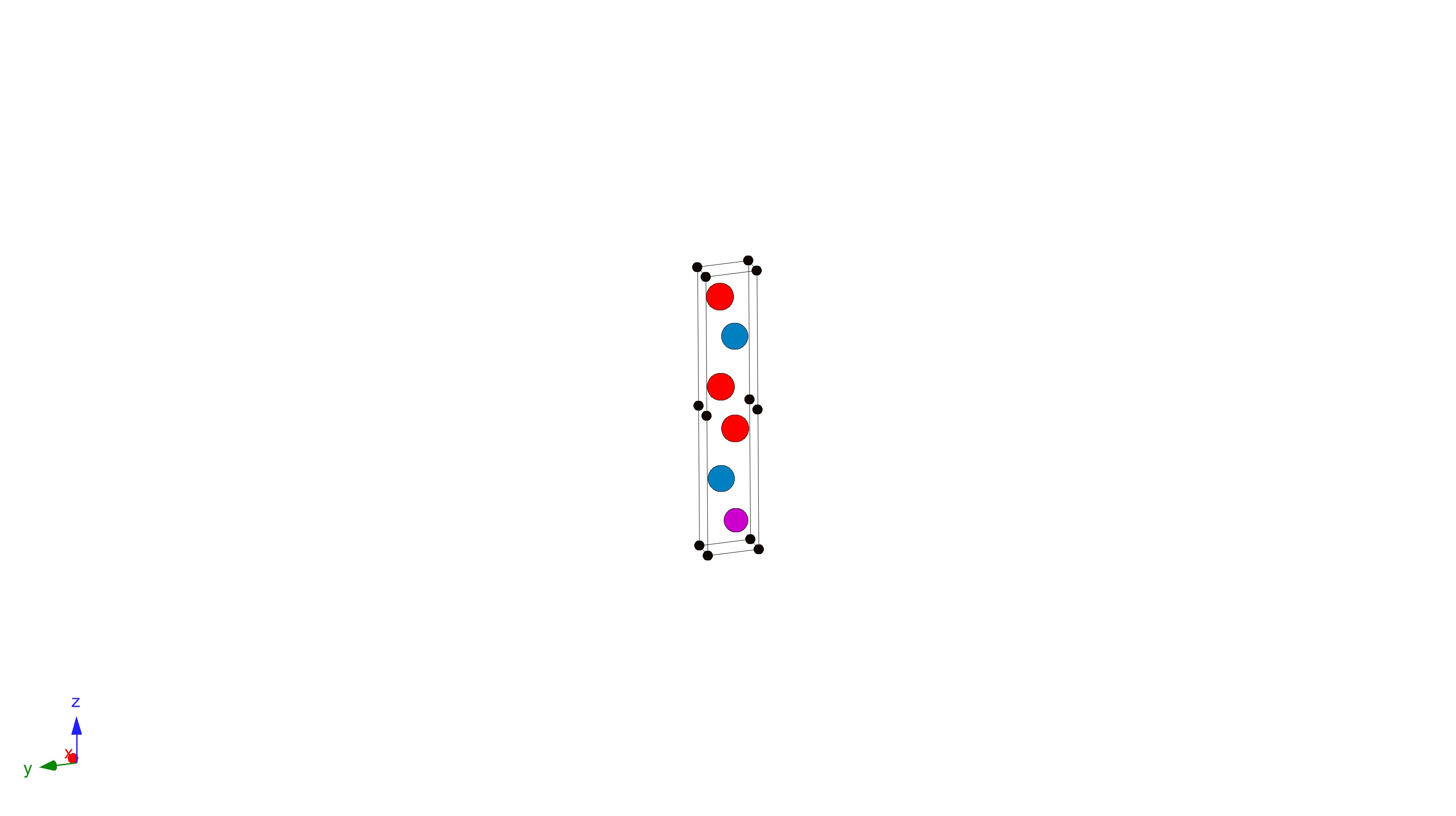

Supplement: Supplementary file 1 [file CP-018-C6CP00802J-s001.zip › mov_alloy_figures/mov2gac/75mo/2f.jpg]

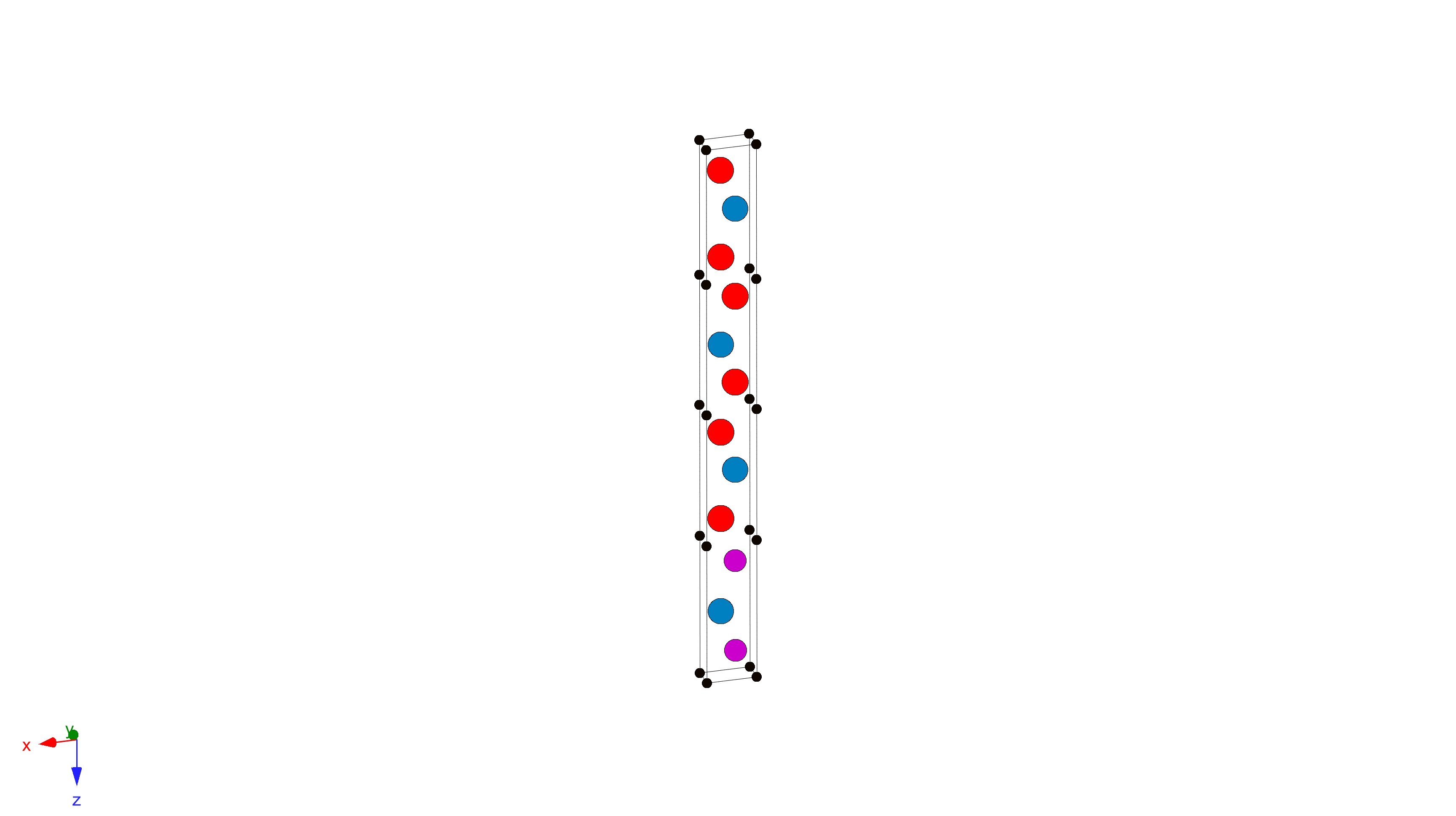

Supplement: Supplementary file 1 [file CP-018-C6CP00802J-s001.zip › mov_alloy_figures/mov2gac/75mo/2g.jpg]

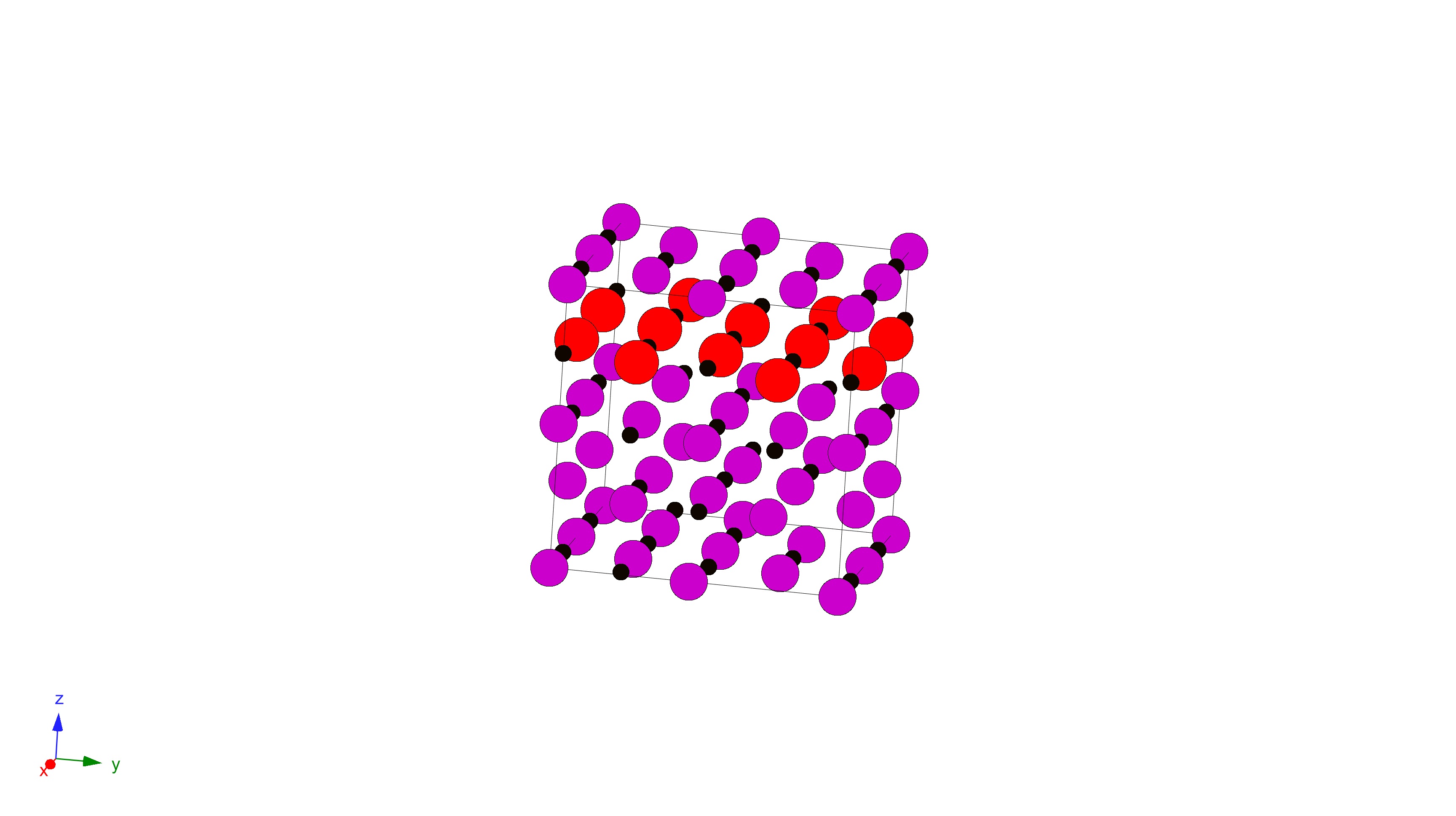

Supplement: Supplementary file 1 [file CP-018-C6CP00802J-s001.zip › mov_alloy_figures/movc with c vacancies/25mo/4a.jpg]

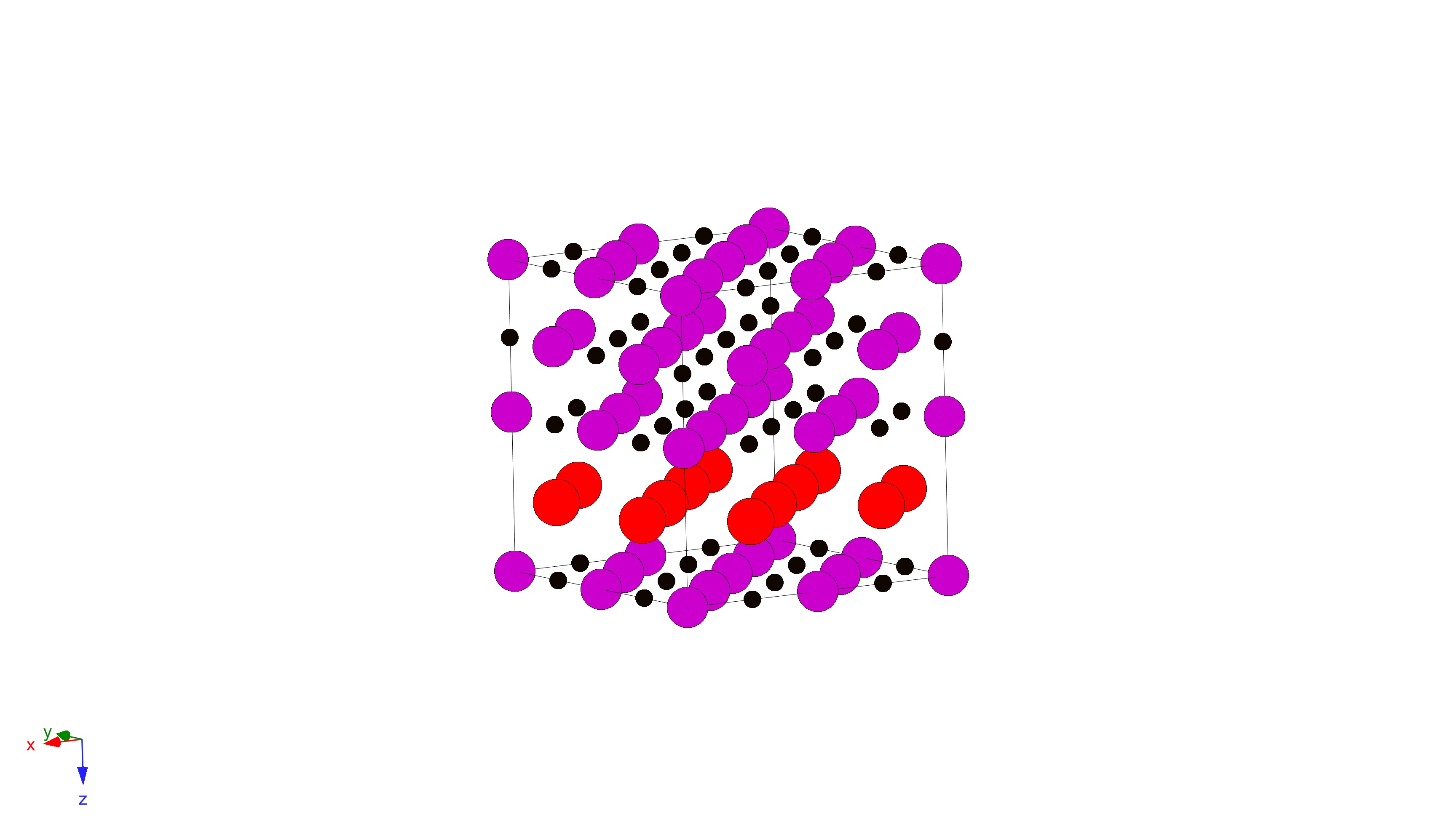

Supplement: Supplementary file 1 [file CP-018-C6CP00802J-s001.zip › mov_alloy_figures/movc with c vacancies/25mo/4b.jpg]

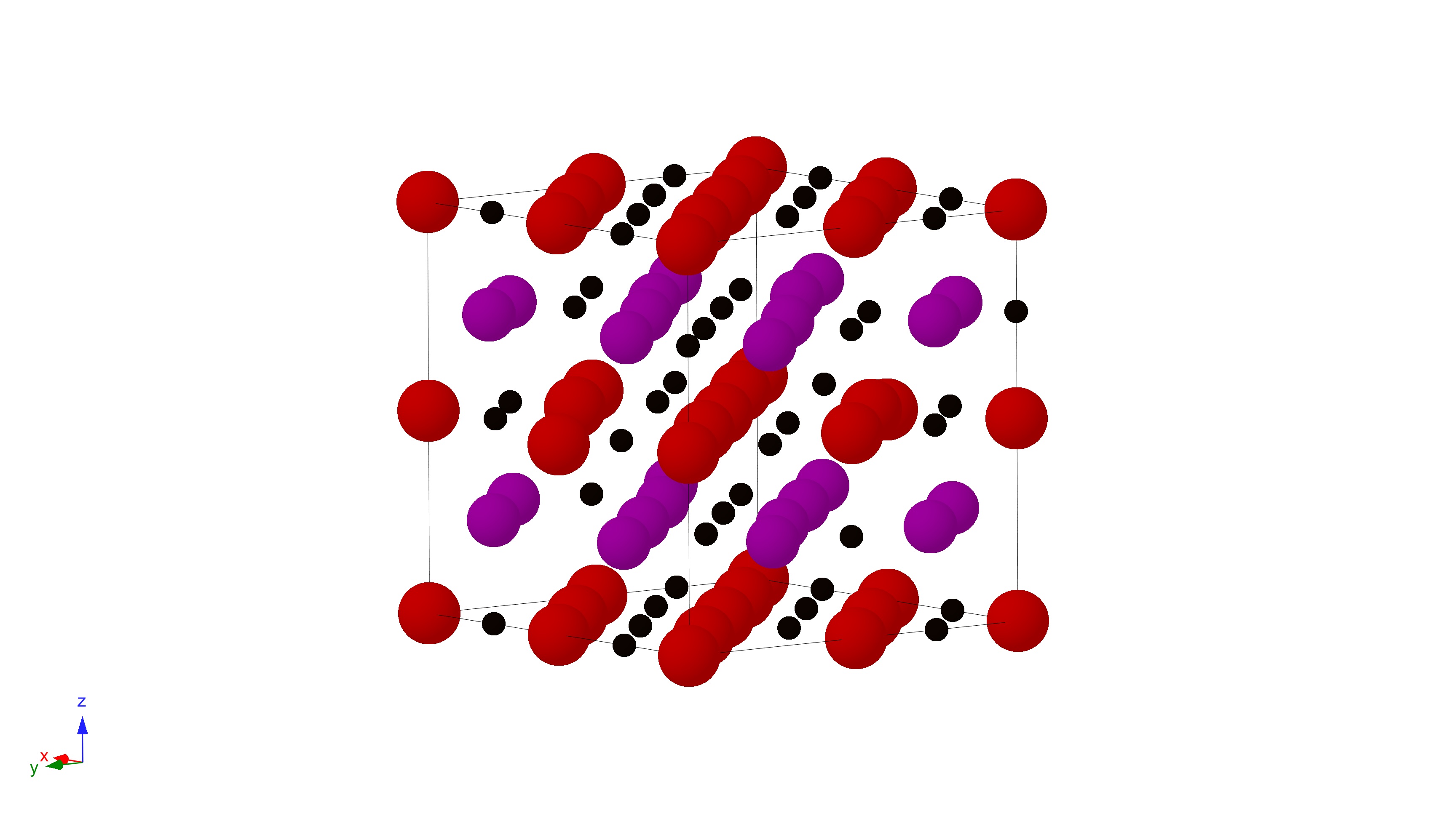

Supplement: Supplementary file 1 [file CP-018-C6CP00802J-s001.zip › mov_alloy_figures/movc with c vacancies/50mo/4c.jpg]

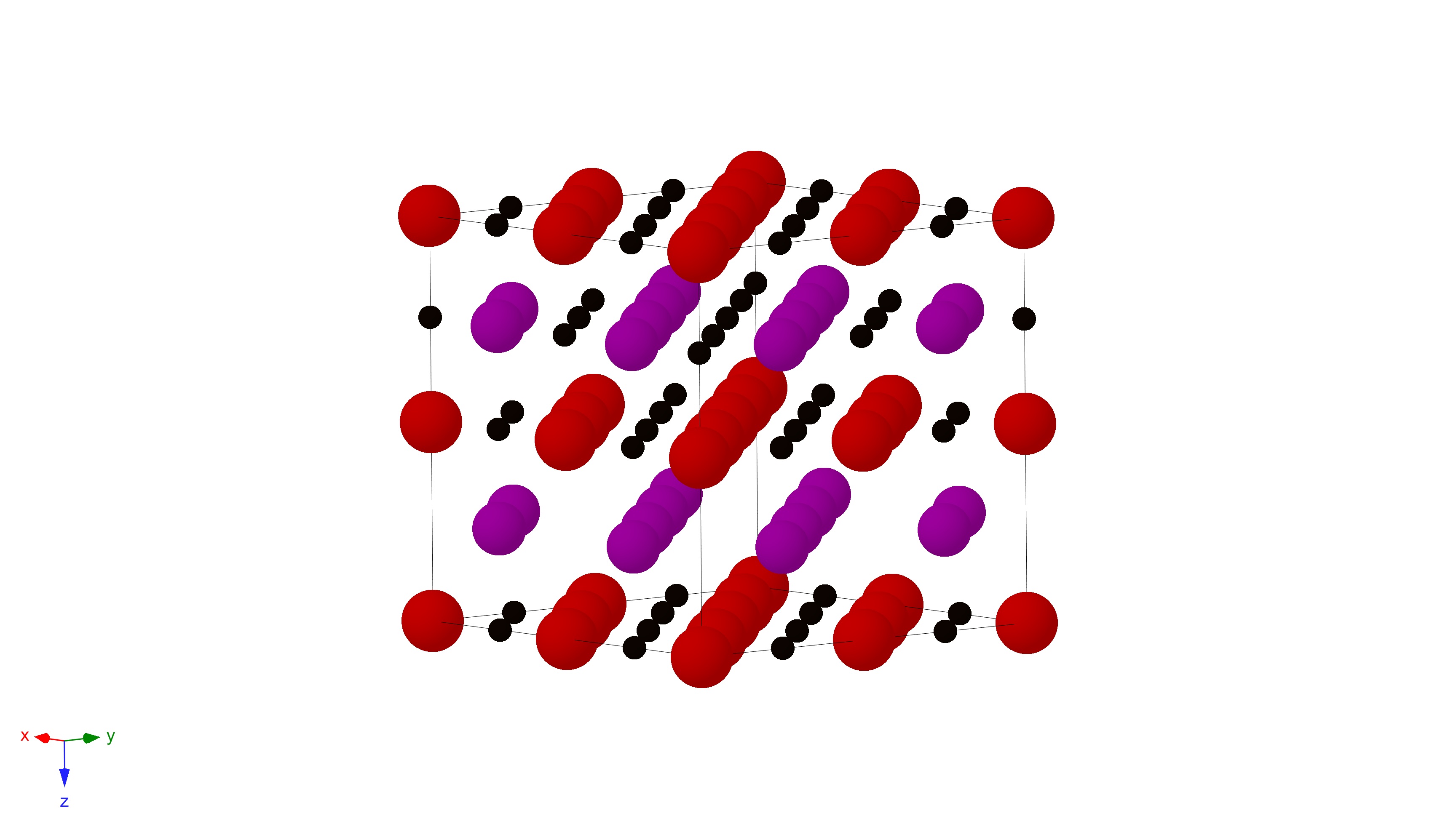

Supplement: Supplementary file 1 [file CP-018-C6CP00802J-s001.zip › mov_alloy_figures/movc with c vacancies/50mo/4d.jpg]

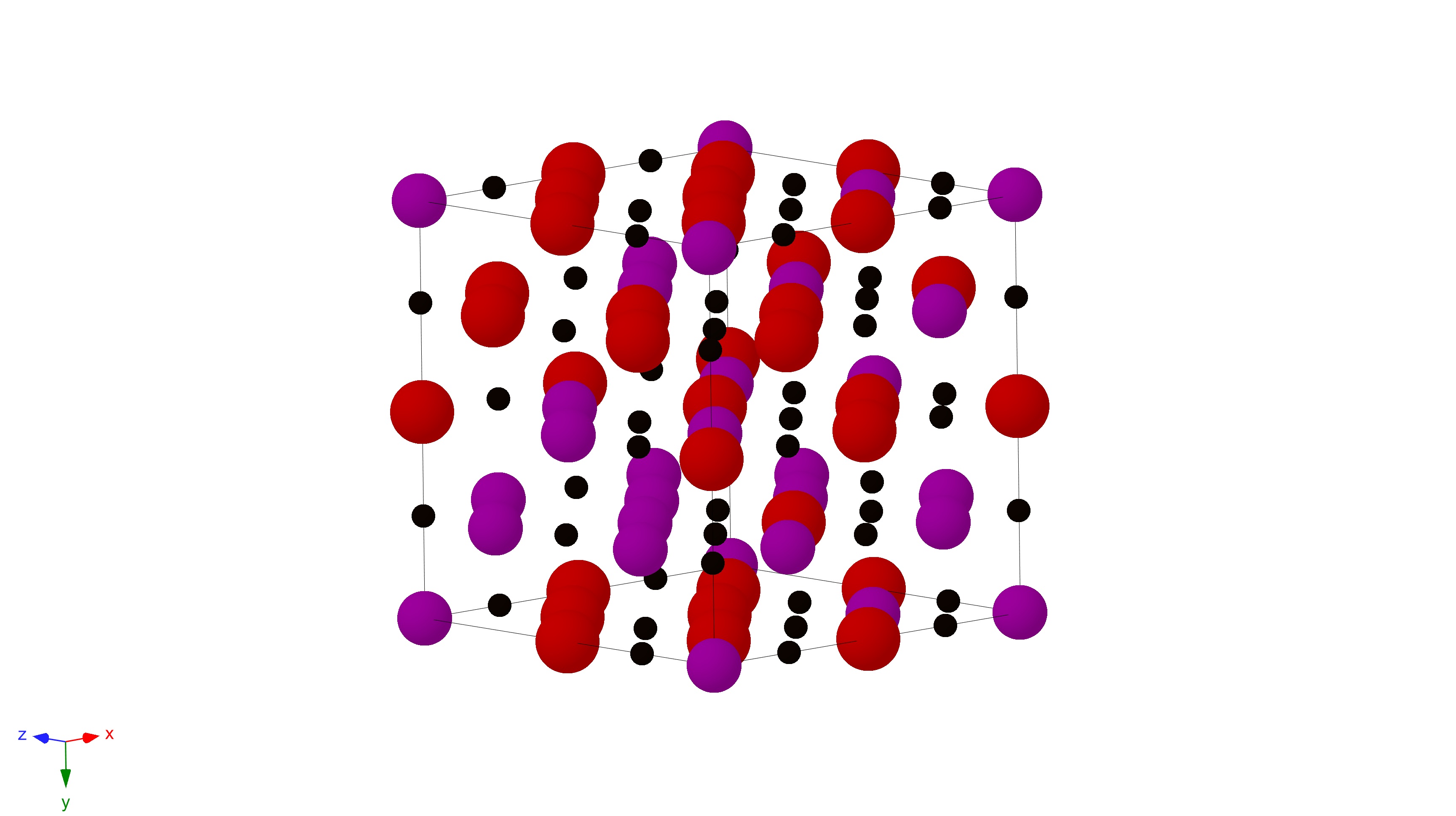

Supplement: Supplementary file 1 [file CP-018-C6CP00802J-s001.zip › mov_alloy_figures/movc with c vacancies/50mo/4e.jpg]

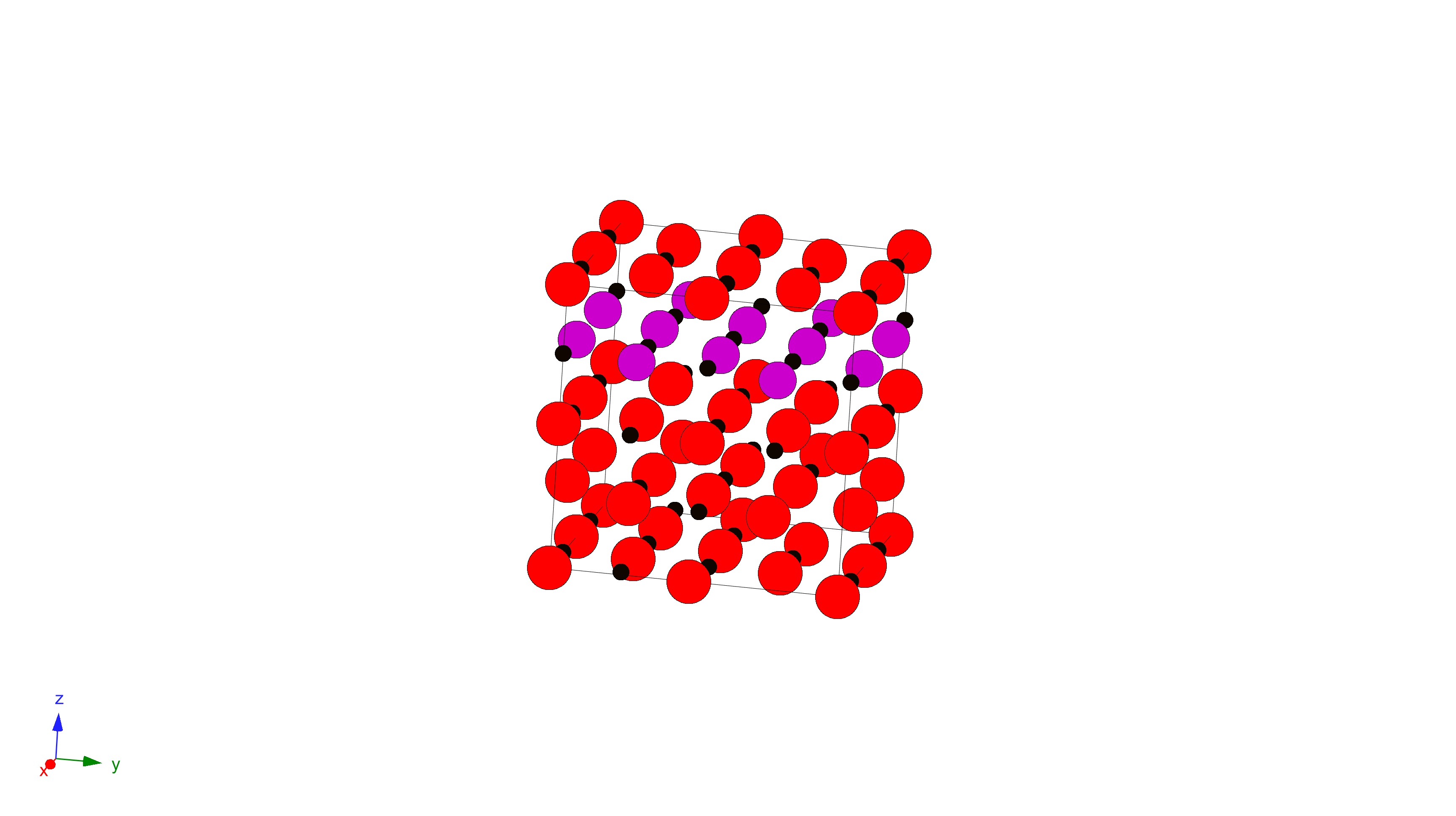

Supplement: Supplementary file 1 [file CP-018-C6CP00802J-s001.zip › mov_alloy_figures/movc with c vacancies/75mo/4f.jpg]

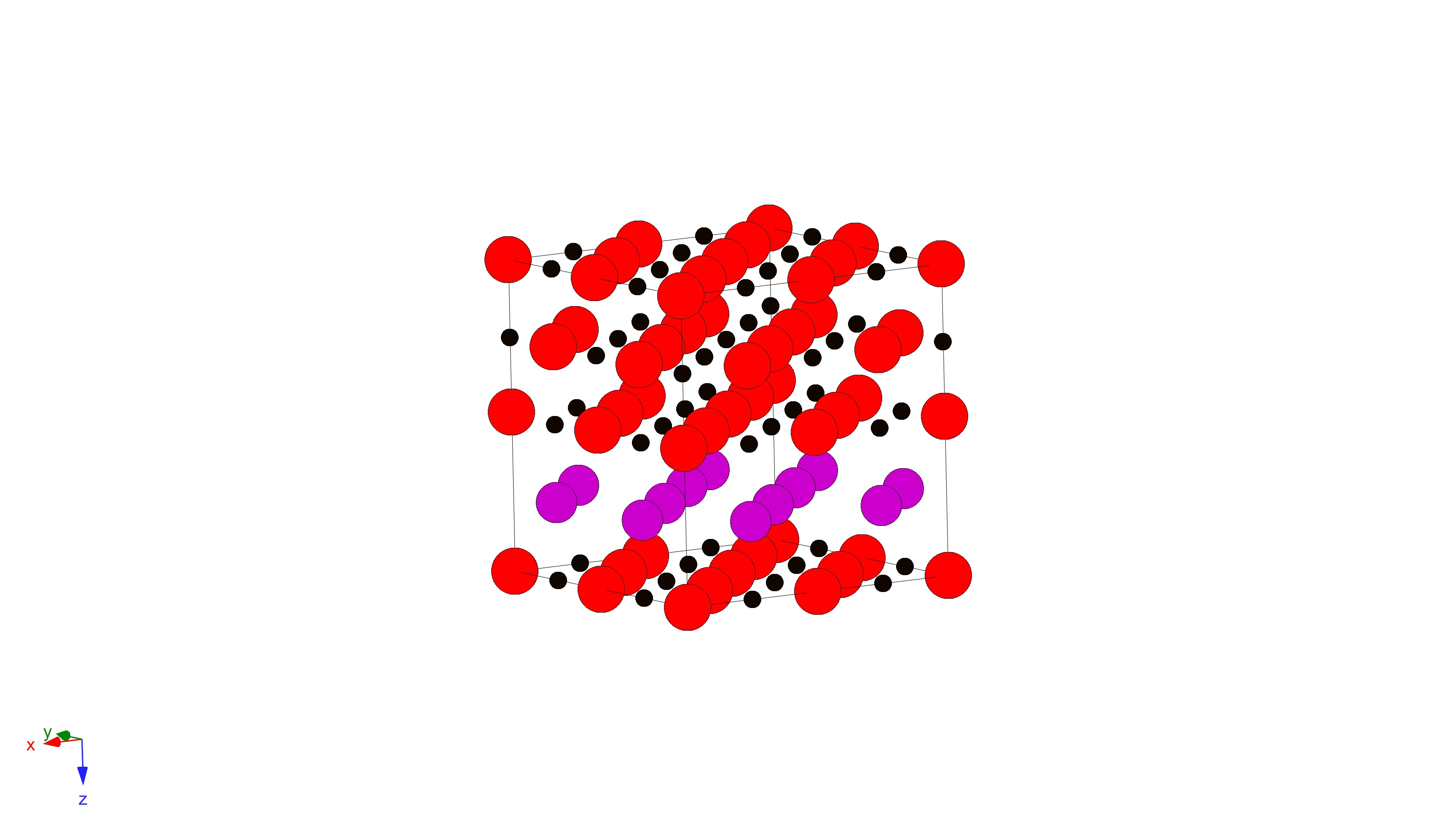

Supplement: Supplementary file 1 [file CP-018-C6CP00802J-s001.zip › mov_alloy_figures/movc with c vacancies/75mo/4g.jpg]

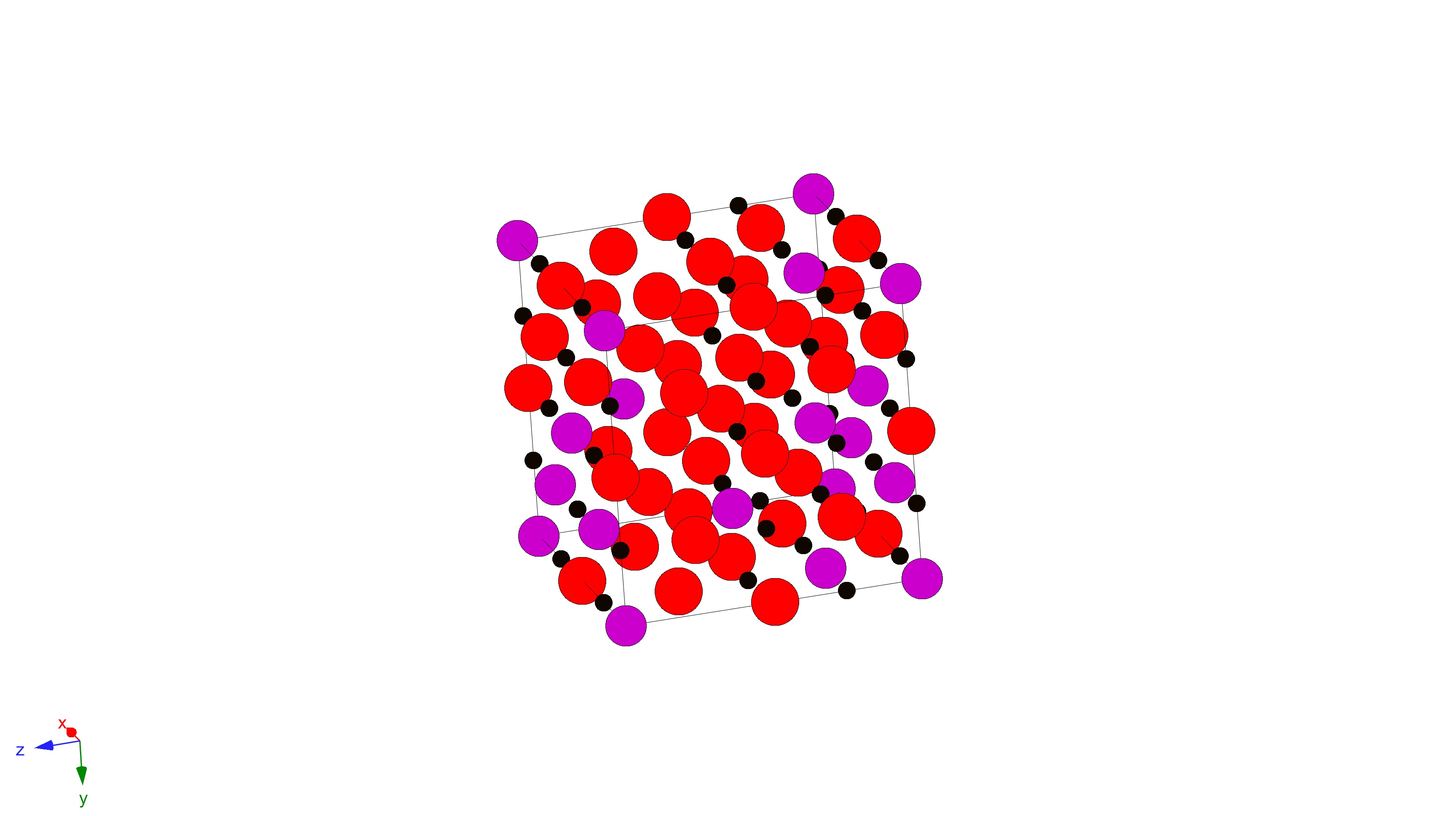

Supplement: Supplementary file 1 [file CP-018-C6CP00802J-s001.zip › mov_alloy_figures/movc with c vacancies/75mo/4h.jpg]

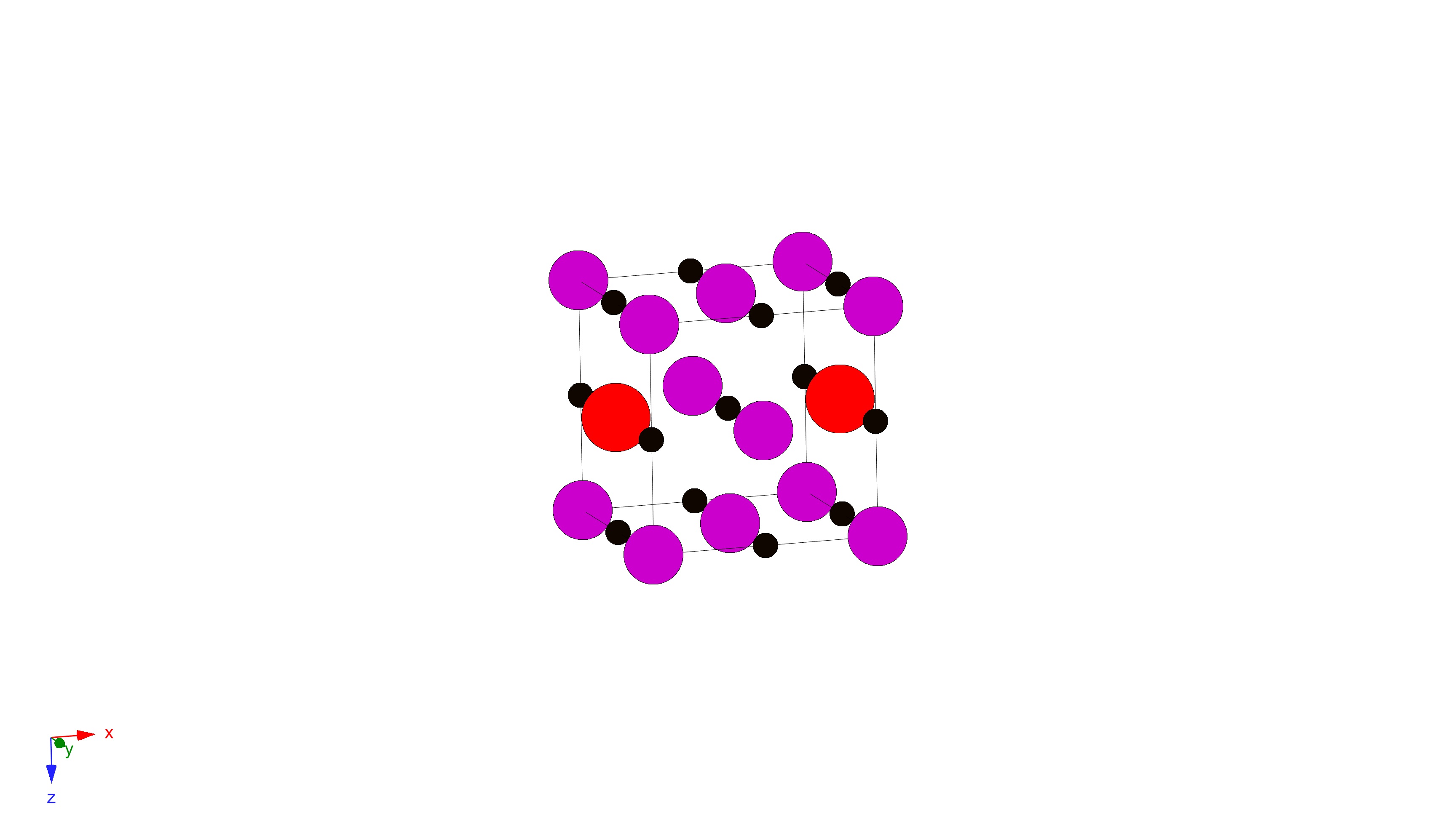

Supplement: Supplementary file 1 [file CP-018-C6CP00802J-s001.zip › mov_alloy_figures/movc/25mo/3a.jpg]

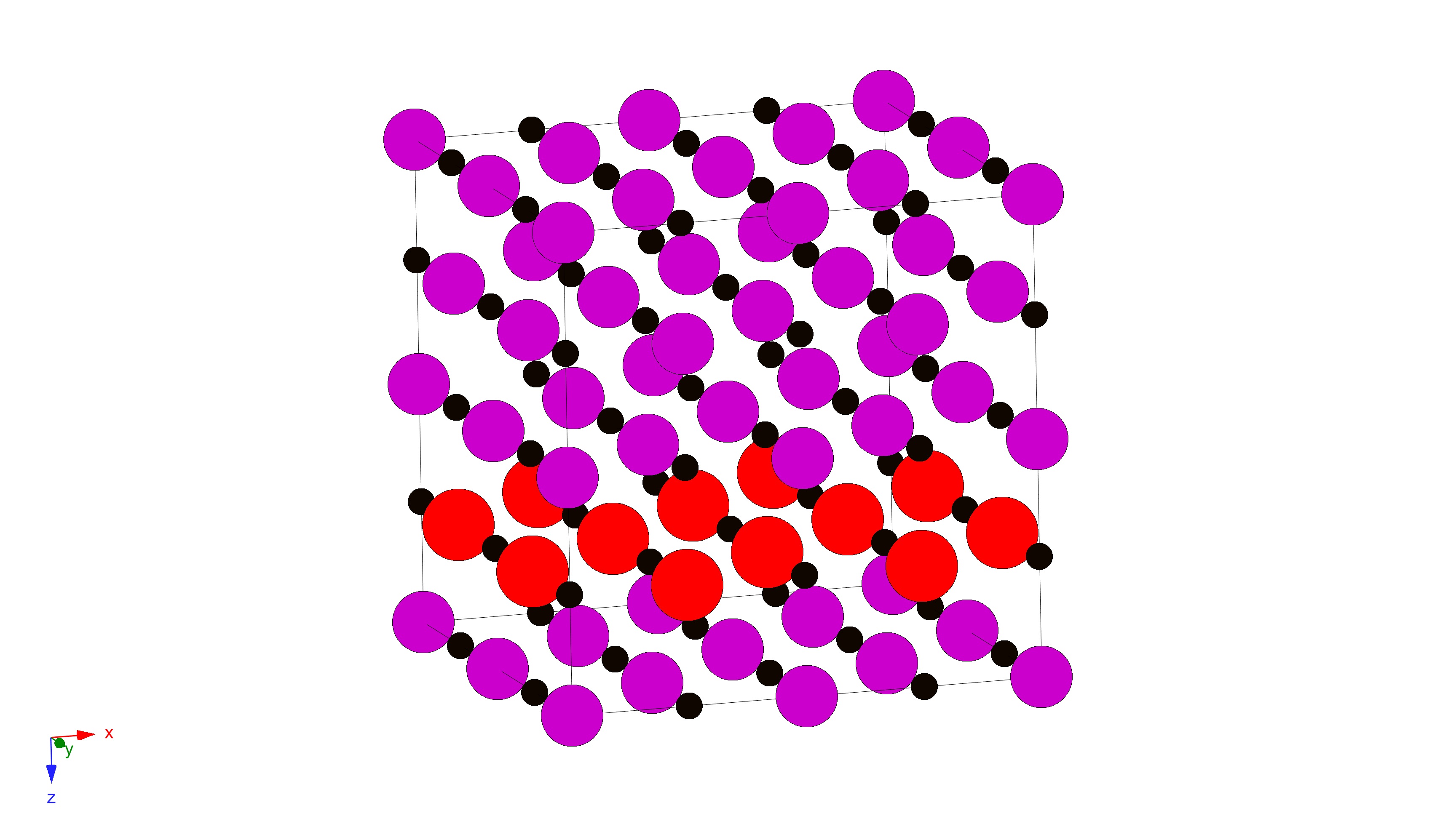

Supplement: Supplementary file 1 [file CP-018-C6CP00802J-s001.zip › mov_alloy_figures/movc/25mo/3b.jpg]

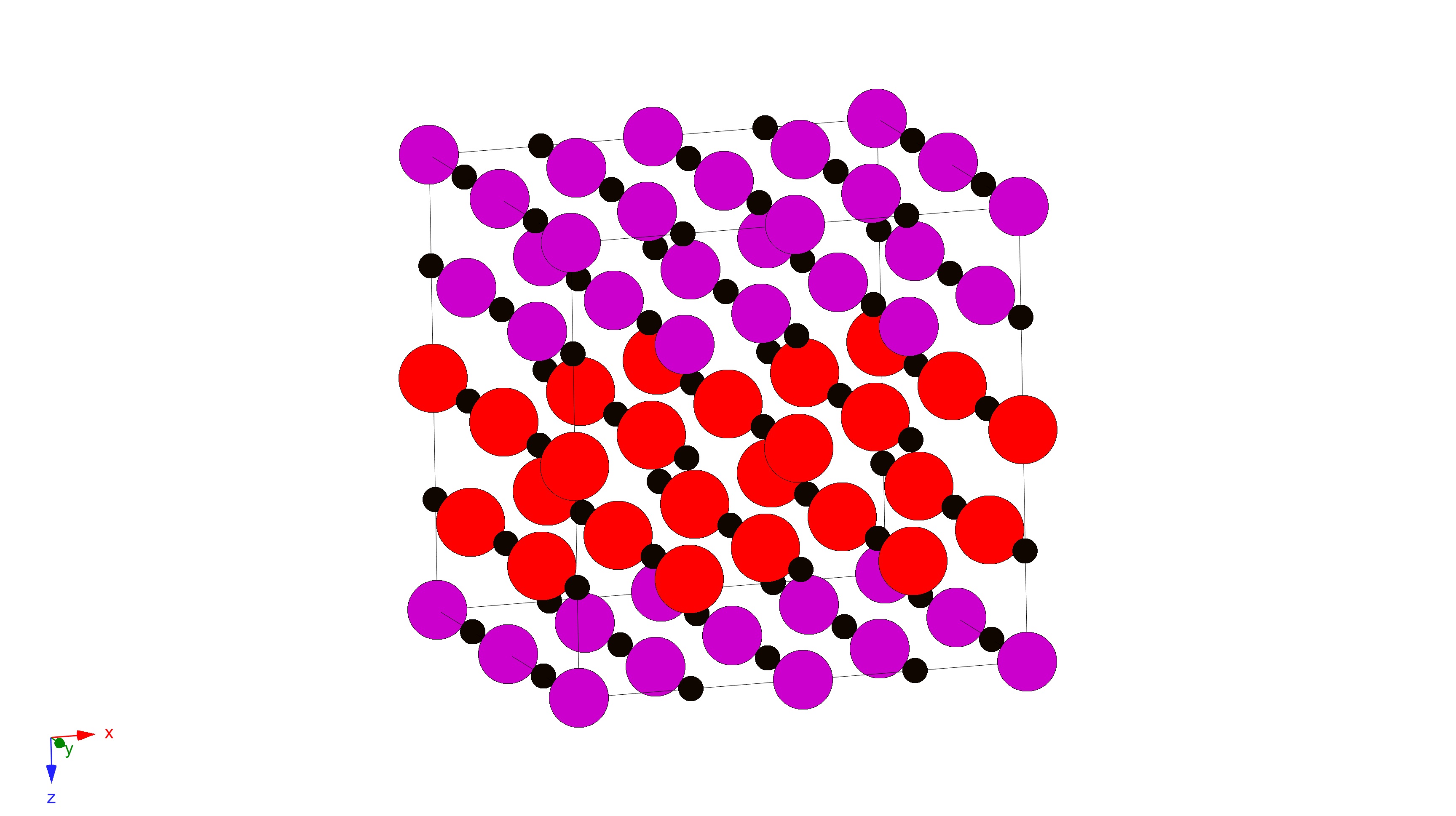

Supplement: Supplementary file 1 [file CP-018-C6CP00802J-s001.zip › mov_alloy_figures/movc/50mo/3c.jpg]

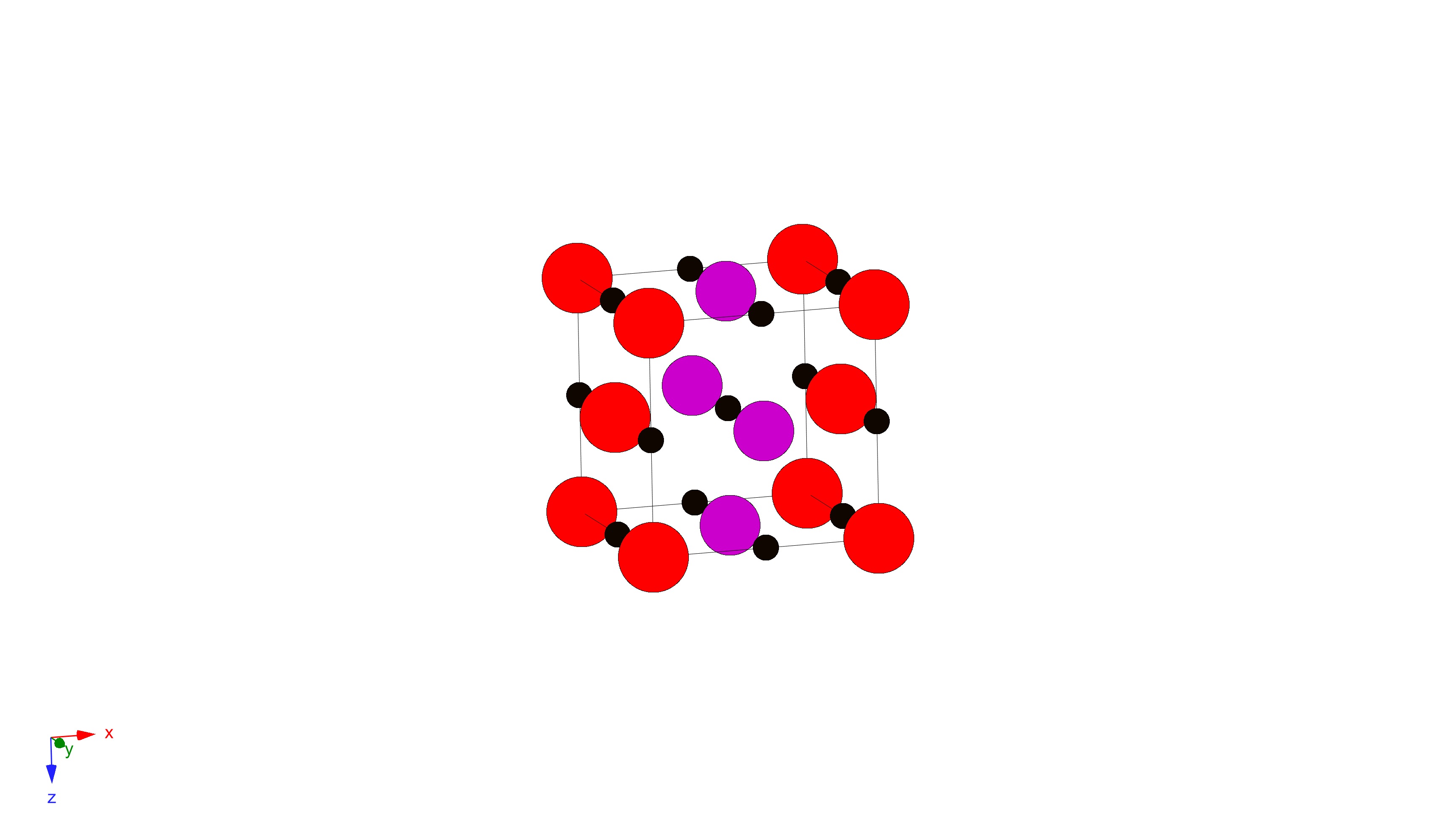

Supplement: Supplementary file 1 [file CP-018-C6CP00802J-s001.zip › mov_alloy_figures/movc/50mo/3d.jpg]

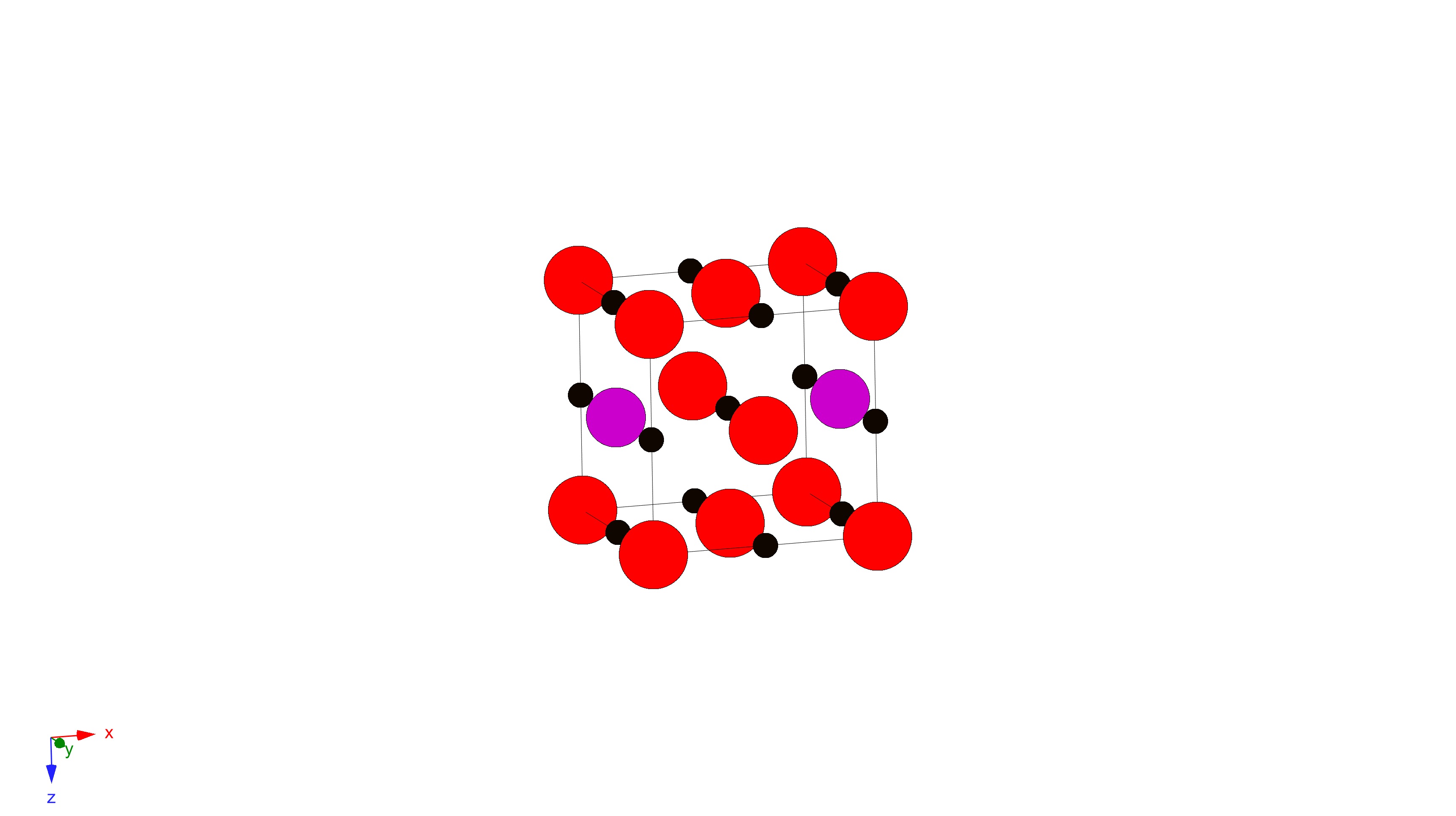

Supplement: Supplementary file 1 [file CP-018-C6CP00802J-s001.zip › mov_alloy_figures/movc/75mo/3e.jpg]

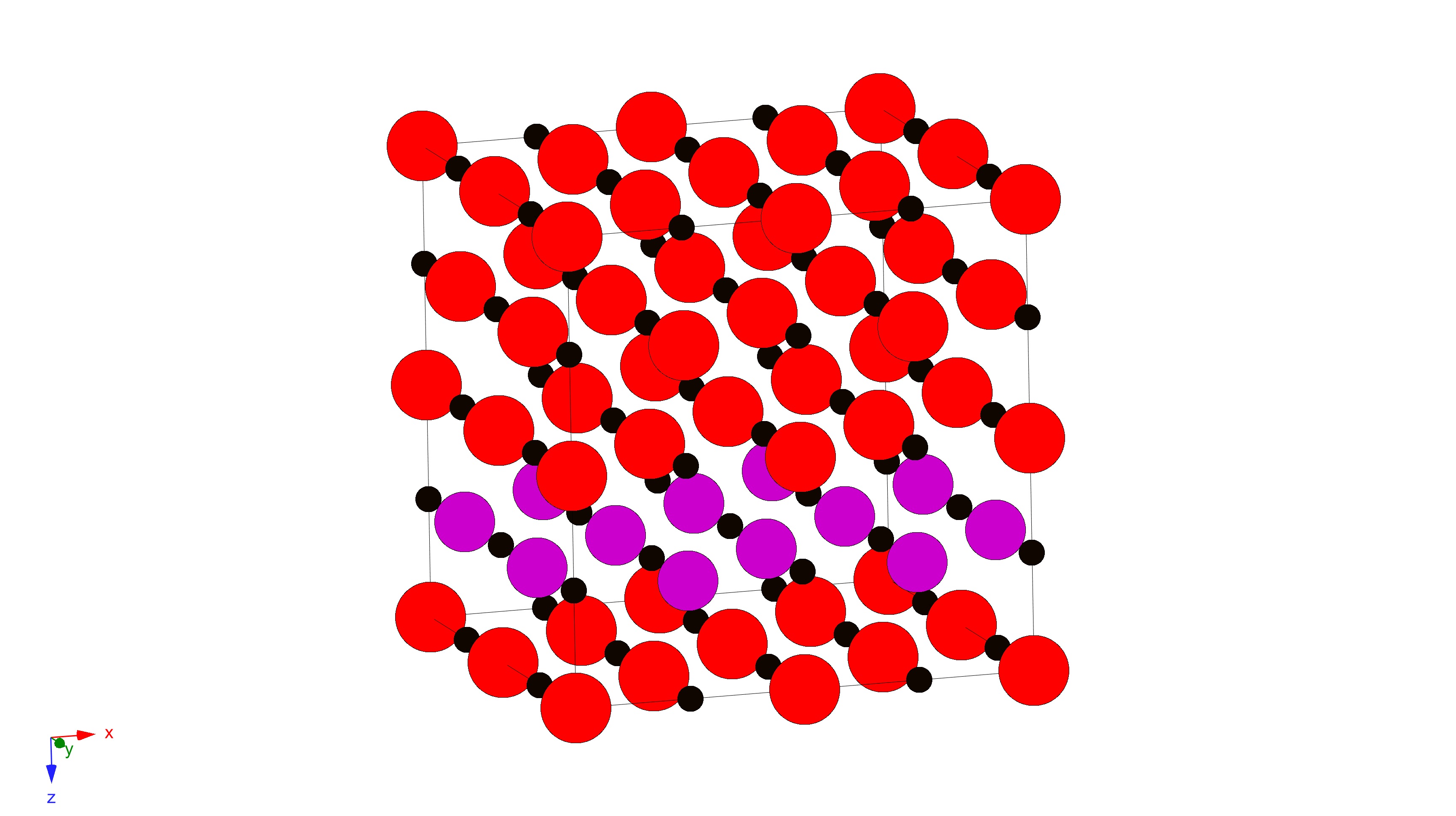

Supplement: Supplementary file 1 [file CP-018-C6CP00802J-s001.zip › mov_alloy_figures/movc/75mo/3f.jpg]
