# Supplementary material for: Phase stability of the nanolaminates V2Ga2C and (Mo1–xVx)2Ga2C from first-principles calculations
Source: Phys Chem Chem Phys. 2016 Apr 20;18(18):12682–8. doi: 10.1039/c6cp00802j (PMC5066482; doi:10.1039/c6cp00802j)
Supplement: Supplementary file 2 [file CP-018-C6CP00802J-s002.pdf]

Electronic Supplementary Material (ESI) for Physical Chemistry Chemical Physics.
This journal is © the Owner Societies 2016

Supplemental Material

Phase stability of the nanonlaminated ternary carbide V_2Ga_2C and the quaternary alloy $(Mo_{1-x}V_x)_2Ga_2C$ from first-principles calculations

A. Thore[a,*], M. Dahlqvist[a], B. Alling[a,b], and J. Rosen[a]

[a] Department of Physics, Chemistry, and Biology, Thin film physics division, Linköping University, SE-581 83 Linköping, Sweden
[b] Department of Computational Materials Design, Max-Planck-Institut für Eisenforschung GmbH, Max-Planck-Straße 1, 40237 Düsseldorf Nordrhein-Westfalen

***Corresponding author. Tel.: +46 70 3212109.**
E-mail address: andth@ifm.liu.se

E-mail addresses to co-authors:
M. Dahlqvist: madah@ifm.liu.se
B. Alling: bjoal@ifm.liu.se
J. Rosén: johro@ifm.liu.se

Table S1. All phases considered in this work, including structural data and enthalpies. For MoV alloys, the first row gives data for a crystal where all sublattices are chemically ordered (unless stated otherwise).

Phase	Prototype structure	Pearson symbol	Space group	a (Å)	b (Å)	c (Å)	H (eV/f.u.)
V	W	cI2	Im-3m (229)	2.994			-9.116
Mo	W	cI2	Im-3m (229)	3.169			-10.850
α-Ga	Ga	oC8	Cmca (64)	4.590	7.753	4.575	-3.030
C	C (graphite)	hP4	$P6_3/mmc$ (194)	2.464		7.250	-9.225
V_3Ga (rt)	Cr_3Si	cP8	Pm-3n (223)	4.791			-31.076
V_6Ga_5 (rt)	α-Ti_6Sn_5	hP22	$P6_3/mmc$ (194)	8.490		5.130	-72.180
V_6Ga_7 (ht)	Cu_5Zn_8	cI52	I-43m (217)	9.171	9.105	9.149	-77.563
V_2Ga_5 (rt)	Mn_2Hg_5	tP14	P4/mbm (127)	9.002		2.684	-34.972
V_8Ga_{41}	V_8Ga_{41}	hR147	R-3 (148)	14.016		14.971	-205.857

Mo_6Ga_{31}	Mo_6Ga_{31}	mP148	$P12_1/c1$ (14)				-163.927
Mo_8Ga_{41}	V_8Ga_{41}	hR147	R-3 h (148)				-217.436
$MoGa_4$	$CrGa_4$	cI10	Im-3m (229)	5.106			-23.818
$MoGa_3$	$TiAl_3$ (NbGa3)	tI8	I4/mmm (139)	5.189		5.428	-18.485
MoGa2	CdI_2 (CrSe2)	hP3	P-3m1 (164)	2.904		6.689	-16.658
Mo_2Ga_3	Ti_2Ga_3	tP10	P4/m (83)	6.515		3.667	-30.851
MoGa	FeSi (CrGe)	cP8	$P2_13$ (198)	5.014			-13.760
Mo_5Ga_4	Ti_5Ga_4 (Nb5Ga4)	hP18	$P6_3/mcm$ (193)	7.817		5.234	-66.314
Mo_3Ga_2	U_3Si_2	tP10	P4/mbm (127)	6.784		3.363	-38.294
Mo_5Ga_3	Cr_5As_3	oP32	Pnma (62)	9.839	7.807	6.639	-62.659
Mo_2Ga	Cu_2Sb	tP6	P4/nmm O2 (129)	3.682		7.062	-24.438
Mo_2Ga	Fe_2P	hP9	P-62m (189)	7.192		3.127	-24.527
Mo_5Ga_2	Mn_5Ge_2	hP42	P3c1 (158)				-59.508
Mo_3Ga	Cr_3Si	cP8	Pm-3n (223)	4.979			-36.200
α-V_2C	PbO_2/Mo_2C	oP12	Pbcn (60)	4.557	5.743	5.037	-28.890
β-V_2C	CdI_2	hP3	$P6_3/mmc$ (194)	2.895		4.532	-28.838
$VC_{0.5}$	NiAs	hP4	$P6_3/mmc$ (194)	2.773		5.194	-13.552
$VC_{0.5}$	NiAs	hP4	$P6_3/mmc$ (194)	2.529		5.067	-13.900
$VC_{0.67}$	$TeSc_{0.67}$	hR24	R-3m (166)	2.918		27.833	-15.951
V_4C_{3-x} (x=0)		hP21	R-3m (166)	2.923		28.017	-67.435
V_6C_5	V_6C_5	hP33	$P3_1 12$ (151)	5.100		15.277	-106.833
V_8C_7	V_8C_7	cP60	P4132 (213)	8.340			-145.426
$VC_{0.875}$	NaCl	cF8	Fm-3m (225)	4.147			-18.035
VC	NiAs	hP4	$P6_3/mmc$ (194)	2.916		5.129	-18.702

VC	NaCl	cF8	Fm-3m (225)	4.164			-19.171
MoC	TiP	hP8	$P6_3/mmc$ (194)	3.016		10.768	-19.821
MoC	NaCl	cF8	Fm-3m (225)	4.383			-19.640
MoC	η-MoC	hp12		3.074		15.401	-19.747
MoC	WC	hp2	P-6m2 (187)	2.928		2.829	-20.241
Mo_3C_2	Cr_3C_2	oP20	Pnma (62)	6.064	2.974	12.654	-50.938
Mo_2C	β''-Mo_2C	hP3	P-3m1 (164)	3.068		4.669	-31.064
Mo_3C	Fe_3C (Cr3C)	oP16	Pnma (62)	5.540	7.559	5.159	-40.423
$Mo_{0.25}V_{0.75}$	W	cl2	Im-3m (229)	3.040	3.040	3.044	-9.615
SQS	W	cl2	Im-3m (229)	3.038	3.044	3.045	-9.613
$Mo_{0.50}V_{0.50}$	W	cl2	Im-3m (229)	3.084	3.084	3.076	-10.082
SQS	W	cl2	Im-3m (229)	3.082	3.087	3.087	-10.076
$Mo_{0.75}V_{0.25}$	W	cl2	Im-3m (229)	3.126	3.126	3.134	-10.509
SQS	W	cl2	Im-3m (229)	3.127	3.129	3.129	-10.499
V_3GaC	$CaTiO_3$	cP5	Pm-3m (221)	3.983			-40.927
V_2GaC	Cr_2AlC	hP8	$P6_3/mmc$ (194)	2.940		12.878	-32.565
V_3GaC_2	Ti_3SiC_2	hP12	$P6_3/mmc$ (194)	2.935		17.744	-51.915
V_4GaC_3	Ti_4AlN_3	hP16	$P6_3/mmc$ (194)	2.932		22.624	-71.244
Mo_3GaC	Mn_3GaC	cP5	Pm-3m (221)	4.152			-44.274
Mo_2GaC	Cr_2AlC	hP8	$P6_3/mmc$ (194)	3.068	4.326	13.272	-34.413
Mo_3GaC_2	Ti_3SiC_2	hP12	$P6_3/mmc$ (194)	3.086		18.358	-54.040
Mo_4GaC_3	Ti_4AlN_3	hP16	$P6_3/mmc$ (194)	3.133		23.116	-73.789

$(Mo_{0.25}V_{0.75})_2GaC$	Cr_2AlC	hP8	$P6_3/mmc$ (194)	2.963		13.018	-33.082
SQS	Cr_2AlC	hP8	$P6_3/mmc$ (194)	2.978		12.945	-33.050
$(Mo_{0.50}V_{0.50})_2GaC$	Cr_2AlC	hP8	$P6_3/mmc$ (194)	2.996		13.124	-33.588
SQS	Cr_2AlC	hP8	$P6_3/mmc$ (194)	3.009		13.030	-33.512
$(Mo_{0.75}V_{0.25})_2GaC$	Cr_2AlC	hP8	$P6_3/mmc$ (194)	3.017		13.349	-33.967
SQS	Cr_2AlC	hP8	$P6_3/mmc$ (194)	3.043		13.108	-33.976
$(Mo_{0.25}V_{0.75})C$	NaCl	cF8	Fm-3m (225)	4.247	4.247	4.227	-19.262
SQS	NaCl	cF8	Fm-3m (225)	4.224	4.230	4.229	-19.296
$(Mo_{0.50}V_{0.50})C$	NaCl	cF8	Fm-3m (225)	4.244	4.287	4.287	-19.384
SQS	NaCl	cF8	Fm-3m (225)	4.285	4.280	4.285	-19.420
$(Mo_{0.75}V_{0.25})C$	NaCl	cF8	Fm-3m (225)	4.349	4.349	4.361	-19.522
SQS	NaCl	cF8	Fm-3m (225)	4.329	4.343	4.321	-19.541
$(Mo_{0.25}V_{0.75})C_{1-x}$ (x=0.25)	NaCl	cF8	Fm-3m (225)	4.129	4.129	4.179	-17.069
SQS	NaCl	cF8	Fm-3m (225)	4.232	4.202	4.217	-17.028
$(Mo_{0.50}V_{0.50})C_{1-x}$ (x=0.25)	NaCl	cF8	Fm-3m (225)	4.266	4.266	4.121	-17.105
SQS on both sublattices	NaCl	cF8	Fm-3m (225)	4.217	4.208	4.227	-17.283
$(Mo_{0.75}V)_{0.25}C_{1-x}$ (x=0.25)	NaCl	cF8	Fm-3m (225)	4.341	4.341	4.179	-17.252
SQS on both sublattices	NaCl	cF8	Fm-3m (225)	4.277	4.257	4.257	-17.456
SQS on *X* sublattice only	NaCl	cF8	Fm-3m (225)	4.261	4.262	4.268	-17.484
$(Mo_{0.25}V_{0.75})_2C$	NiAs	hP4	$P6_3/mmc$ (194)	2.949		4.581	-29.444
SQS	NiAs	hP4	$P6_3/mmc$ (194)	2.946		4.560	-29.042

SQS on *X* sublattice only	NiAs	hP4	$P6_3/mmc$ (194)	2.946		4.560	-29.451
$(Mo_{0.50}V_{0.50})_2C$	NiAs	hP4	$P6_3/mmc$ (194)	2.984		4.588	-30.087
SQS	NiAs	hP4	$P6_3/mmc$ (194)	2.993		4.586	-30.035
$(Mo_{0.75}V_{0.25})_2C$	NiAs	hP4	$P6_3/mmc$ (194)	3.036		4.621	-30.591
SQS	NiAs	hP4	$P6_3/mmc$ (194)	3.035		4.621	-30.559
V_2Ga_2C	221	hP10	$P6_3/mmc$ (194)	2.946		17.861	-35.617
$(Mo_{0.125}V_{0.875})_2Ga_2C$ SQS	221	hP10	$P6_3/mmc$ (194)	2.960		17.917	-35.894
$(Mo_{0.25}V_{0.75})_2Ga_2C$	221	hP10	$P6_3/mmc$ (194)	2.957		18.092	-36.210
SQS	221	hP10	$P6_3/mmc$ (194)	2.975		17.978	-36.158
$(Mo_{0.333}V_{0.667})_2Ga_2C$	221	hP10	$P6_3/mmc$ (194)	2.966		18.102	-36.403
$(Mo_{0.375}V_{0.625})_2Ga_2C$ SQS	221	hP10	$P6_3/mmc$ (194)	2.984		18.006	-36.414
$(Mo_{0.5}V_{0.5})_2Ga_2C$	221	hP10	$P6_3/mmc$ (194)	3.003		18.062	-36.689
SQS	221	hP10	$P6_3/mmc$ (194)	3.001		18.077	-36.668
$(Mo_{0.625}V_{0.375})_2Ga_2C$ SQS	221	hP10	$P6_3/mmc$ (194)	3.012		18.101	-36.920
$(Mo_{0.667}V_{0.333})_2Ga_2C$	221	hP10	$P6_3/mmc$ (194)	3.010		18.201	-37.105
$(Mo_{0.75}V_{0.25})_2Ga_2C$	221	hP10	$P6_3/mmc$ (194)	3.028		18.256	-37.256
SQS	221	hP10	$P6_3/mmc$ (194)	3.034		18.134	-37.171
$(Mo_{0.875}V_{0.125})_2Ga_2C$ SQS	221	hP10	$P6_3/mmc$ (194)	3.052		18.129	-37.424
Mo_2Ga_2C	221	hP10	$P6_3/mmc$ (194)	3.064		18.153	-37.692
$V_3Ga_2C_2$	322	hP14	$P6_3/mmc$ (194)	2.953		22.621	-55.034
$Mo_3Ga_2C_2$	322	hP14	$P6_3/mmc$ (194)	3.085		23.177	-57.306

$VMo_2Ga_2C_2$	322	hP14	$P6_3/mmc$ (194)	3.001		23.251	-57.006
$MoV_2Ga_2C_2$	322	hP14	$P6_3/mmc$ (194)	3.006		22.854	-55.571
$V_4Ga_2C_3$	423	hP18	$P6_3/mmc$ (194)	2.936		27.577	-74.302
$Mo_4Ga_2C_3$	423	hP18	$P6_3/mmc$ (194)	3.145		27.708	-77.008
$(VMo_2)GaC_2$	312 biMAX (type A)	hP12	$P6_3/mmc$ (194)	2.996		18.442	-53.722

Table S2. All different ordered alloys considered in this work, including supercell sizes and enthalpies. The figures can be found in the folder "mov_alloy_figures".

Phase	Figure	Supercell size	H (eV/f.u.)
$Mo_{0.25}V_{0.75}$	1a	2x2x2	-9.615
	1b	4x4x4	-9.597
$Mo_{0.5}V_{0.5}$	1c	2x2x2	-10.082
	1d	2x2x2	-10.077
	1e	2x2x2	-10.077
	1f	4x4x4	-10.028
$Mo_{0.75}V_{0.25}$	1g	2x2x2	-10.509
	1h	4x4x4	-10.473
$(Mo_{0.25}V_{0.75})_2GaC$	2a	1x1x1	-33.082
	2b	1x1x2	-33.074
$(Mo_{0.5}V_{0.5})_2GaC$	2c	1x1x1	-33.576
	2d	1x1x1	-33.588
	2e	1x1x1	-33.455
$(Mo_{0.75}V_{0.25})_2GaC$	2f	1x1x1	-33.967
	2g	1x1x2	-33.964
$Mo_{0.25}V_{0.75}C$	3a	1x1x1	-19.262
	3b	2x2x2	-19.262
$Mo_{0.5}V_{0.5}C$	3c	2x2x2	-19.374

	3d	1x1x1	-19.384
$Mo_{0.75}V_{0.25}C$	3e	1x1x1	-19.521
	3f	2x2x2	-19.522
$(Mo_{0.25}V_{0.75})C_{1-x}$ (x=0.25)	4a	2x2x2	-16.985
	4b	2x2x2	-17.069
$(Mo_{0.5}V_{0.5})C_{1-x}$ (x=0.25)	4c	2x2x2	-17.227
	4d	2x2x2	-17.105
	4e	2x2x2	-17.128
$(Mo_{0.75}V_{0.25})C_{1-x}$ (x=0.25)	4f	2x2x2	-17.484
	4g	2x2x2	-17.252
	4h	2x2x2	-17.415
$(Mo_{0.25}V_{0.75})_2C$	5a	4x4x2	-30.559
	5b	2x2x2	-30.591
	5c	4x4x2	-30.150
$(Mo_{0.5}V_{0.5})_2C$	5d	4x4x2	-30.035
	5e	2x2x2	-30.087
	5f	4x4x2	-29.616
$(Mo_{0.75}V_{0.25})_2C$	5g	4x4x2	-30.559
	5h	2x2x2	-30.591
	5i	4x4x2	-30.150
$(Mo_{0.25}V_{0.75})_2Ga_2C$	6a	1x1x1	-36.146
	6b	1x1x2	-36.210
	6c	2x1x1	-36.133
$(Mo_{0.333}V_{0.667})_2Ga_2C$	6d	1x1x3	-36.403
	6e	$\sqrt{3}$x$\sqrt{3}$x1	-36.115
$(Mo_{0.5}V_{0.5})_2Ga_2C$	6f	1x1x1	-36.682
	6g	1x1x1	-36.689
	6h	1x1x1	-36.620
	6i	1x1x2	-36.616
	6j	2x1x1	-36.648
	6k	2x1x1	-36.658
	6l	4x1x1	-36.687
	6m	4x1x1	-36.648
	6n	4x1x1	-36.662
	6o	4x1x1	-36.682
	6p	4x1x1	-36.661

	6q	4x1x1	-36.682
	6r	1x1x3	-36.687
$(Mo_{0.667}V_{0.333})_2Ga_2C$	6s	1x1x3	-37.105
	6t	$\sqrt{3}$x$\sqrt{3}$x1	-36.821
$(Mo_{0.75}V_{0.25})_2Ga_2C$	6u	1x1x1	-37.181
	6v	1x1x2	-37.256
	6w	1x1x2	-37.169
